# New electrical impedance methods for the *in situ* measurement of the complex permittivity of anisotropic skeletal muscle using multipolar needles

**DOI:** 10.1038/s41598-019-39277-0

**Published:** 2019-02-28

**Authors:** H. Kwon, M. Guasch, J. A. Nagy, S. B. Rutkove, B. Sanchez

**Affiliations:** 1 Department of Neurology, Beth Israel Deaconess Medical Center, Harvard Medical School, Boston, MA 02215-5491 USA; 2 Present Address: College of Science of & Technology, Yonsei University, Wonju, 26493 Republic of Korea

## Abstract

This paper provides a rigorous analysis on the measurement of the permittivity of two-dimensional anisotropic biological tissues such as skeletal muscle using the four-electrode impedance technique. The state-of-the-art technique requires individual electrodes placed at the same depth in contact with the anisotropic material, e.g. using monopolar needles. In this case, the minimum of measurements in different directions needed to estimate the complex permittivity and its anisotropy direction is 3, which translates into 12 monopolar needle insertions (i.e. 3 directions × 4 electrodes in each direction). Here, we extend our previous work and equip the reader with 8 new methods for multipolar needles, where 2 or more electrodes are spaced along the needle’s shaft in contact with the tissue at different depths. Using multipolar needles, the new methods presented reduce the number of needle insertions by a factor of 2 with respect to the available methods. We illustrate the methods with numerical simulations and new experiments on *ex vivo* ovine skeletal muscle (n = 3). Multi-frequency longitudinal and transverse permittivity data from 30 kHz to 1 MHz is made publicly available in the supplementary material. The methods presented here for multipolar needles bring closer the application of needle electrical impedance to patients with neuromuscular diseases.

## Introduction

Electromagnetic fields (EMF) constitute a fundamental physical principle used today for imaging and treating a broad spectrum of conditions including Parkinson disease^1^ and brain tumors^2,3^. Understanding how the human body interacts with EMF through the complex permittivity property –or simply permittivity–^4^, of biological tissues and fluids is paramount to improve the accuracy of existing techniques as well as to develop new diagnostic tools and therapeutic treatments, for example, for assessment of radiation injury^5,6^.

Measuring the permittivity of tissues has been an encompassing effort from many researchers for almost one hundred years^7–11^. Unfortunately, to this day, there are still important gaps in knowledge of the *in situ* values of these properties in many tissues, especially as they relate to anisotropic tissues such as skeletal muscle. Indeed, most of the studies have measured the permittivity of tissues by performing *ex situ* measurements from biopsy samples. Whereas this may be the simplest approach to measure the permittivity of anisotropic tissues, it is technically challenging to perform and requires the investigator to perform a biopsy procedure. More importantly, *ex situ* measurements may not be representative of the permittivity measured *in situ* on alive tissue, as this property is known to change postmortem^12^ and with temperature^10,13^.

However, measuring *in situ* the anisotropic nature of permittivity confers an added difficulty in the measurement itself. Importantly, existing *in situ* methods have important limitations which prevent the user from measuring the anisotropic permittivity accurately^14–16^. Among the limitations, these methods (i) consider a *purely* conductive anisotropic material, (ii) require *exact* knowledge on the anisotropy direction defined by the *unknown* tissue’s permittivity, and (iii) require aligning *perfectly* the four-electrode probe in the anisotropy direction.

In our previous work^17^, we overcame the existing limitations and developed more sophisticated methods to measure the permittivity using *mono*polar needles by placing four needles (i.e. four electrodes) in contact with the anisotropic tissue. Among the results, we found that when the direction of the anisotropic permittivity is unknown (i.e. the most general case), then it is necessary to measure the impedance with the four-electrode probe in three or more different directions; otherwise, the error in positioning the four-electrode probe may jeopardize the validity of the calculated permittivity. A consequence that follows from this observation is that when the four-electrode probe is made from *monopolar* needles, e.g. those used for intramuscular electromyographic recordings, it is necessary to perform at least twelve needle insertions to access inner tissues such as skeletal muscle from the outside. While this may be acceptable in pre-clinical studies, for example to access skeletal muscle tissue through the skin and subcutaneous fat, successful clinical translation of these methods requires the reduction of the number of needle insertions in order to minimize patient discomfort.

The goal of this study is to propel the scientific understanding of anisotropic tissues by enhancing the methods to measure the permittivity of such materials. This article equips the reader with eight new methods, of increasing computational difficulty, to measure the permittivity of anisotropic biological tissues by combining the four-electrode electrical impedance technique with *multipolar* needles^18^. As opposed to the state-of-the-art technique based on *monopolar* needles^17^, the methods presented in this paper, developed for *multipolar* needles, allow for the reduction in the required number of needle insertions from 12 to 6 this approaching practical application in the clinic.

This a methods paper and it is organized as follows. First, we evaluate the accuracy of the new methods developed by performing numerical simulations and *in situ* experiments on *ex vivo* ovine tissue. Next, we discuss the implications of the new methodology. Materials and methods follow thereafter, with particular emphasis on the novel contribution of our work. For completeness and consistency with our previous^17^, we briefly review the mathematical background on which all methods presented are based. The first case studied considers multipolar needles with two electrodes changing the angle of measurement. Then, we extend the results to needle devices integrating more than two electrodes while keeping constant the measurement angle. The two previous approaches are then combined to measure the permittivity when the tissues’ anisotropy direction is unknown. The advanced reader can find a rigorous step-by-step derivation of the methods in the supplementary material, otherwise, the information necessary to use each method is contained in the main document.

## Results

### Simulation

#### Benchmark results: simulation example of methods H1, H2 and H3

The methods developed for multipolar needles are simulated following the same rationale as our previous work to facilitate comparison with the state-of-the-art based on monopolar needles^17^. We used MATLAB software (The Mathworks, Inc., Natick, MA) to generate *M* = 10 noisy apparent resistivity and apparent reactivity values giving a signal-to-noise ratio (SNR) of 30 dB. Further, the needle-distance aspect ratio was considered *p* = 8.6. The true anisotropic permittivity of muscle at 10 kHz was taken from a database^19^, namely the transverse resistivity *ρ*_T_ = 2.93 Ω m and the transverse relative permittivity *ε*_*r*,T_ = 2.59 ⋅ 10^4^ (dimensionless), denoted by the subscript T. We compute the longitudinal (denoted by the subscript L) permittivity from the transverse permittivity values using an anisotropy ratio *α*^2^ = 0.5, i.e. *ρ*_L_ = 1.47 Ω m and *ε*_*r*,L_ = 1.30 ⋅ 10^4^ (dimensionless). We represent the apparent resistivity and apparent reactivity in (3) in polar plots to show its angular dependence.

**Figure 1:**
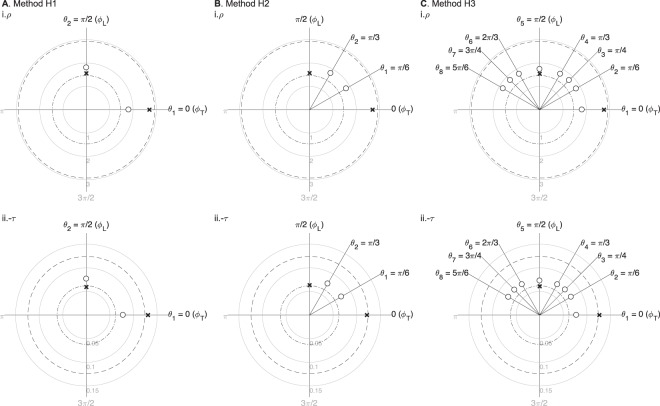
Numerical simulation results using methods H1 (**A**), H2 (**B**), and H3 (**C**). The estimated impedivity in longitudinal (L) and transverse (T) directions ${\hat{\kappa }}_{\{L,{\rm{T}}\}}$ (shown in crosses) were calculated from *M* = 10 averaged apparent impedivity ${\hat{\kappa }}_{{\rm{a}}}({\theta }_{d})$ measurements (shown in circles), with *d* = 1, …, *D* the measurement angles and inter-electrodes’ distance *p* = 8.6. Method H1 (*D* = 2): ${\hat{\rho }}_{{\rm{L}}}=1.56$ Ω m, ${\hat{\rho }}_{{\rm{T}}}=2.71$ Ω m, ${\hat{\tau }}_{{\rm{L}}}=-\,0.06$ Ω m, ${\hat{\tau }}_{{\rm{T}}}=-\,0.13$ Ω m; Method H2 (*D* = 2): ${\hat{\rho }}_{{\rm{L}}}=1.57$ Ω m and ${\hat{\rho }}_{{\rm{T}}}=2.71$ Ω m, ${\hat{\tau }}_{{\rm{L}}}=-\,0.06$ Ω m, ${\hat{\tau }}_{{\rm{T}}}=-\,0.12$ Ω m; Method H3 (*D* = 8): ${\hat{\rho }}_{{\rm{L}}}=1.54$ Ω m, ${\hat{\rho }}_{{\rm{T}}}=2.76$ Ω m, ${\hat{\tau }}_{{\rm{L}}}=-\,0.06$ Ω m, ${\hat{\tau }}_{{\rm{T}}}=-\,0.13$ Ω m. The true longitudinal and transverse resistivity (i) and reactivity (ii) are plotted in dash-dot and dashed circumferences, *ρ*_L_ = 1.46 Ω m, *ρ*_T_ = 2.93 Ω m, *τ*_L_ = −0.06 Ω m and *τ*_T_ = −0.12 Ω m, respectively. The reactivity is shown −*τ* as a convention. The units of impedivity are Ω m.

Figure 1 shows a single-frequency comparison of methods H1 to H3 measuring the anisotropic permittivity of skeletal muscle tissue. The angles considered in method H1 are the longitudinal and transverse directions defined by the anisotropy in the material, i.e. *ϕ*_L_ = *π*/2 and *ϕ*_T_ = 0 respectively (Fig. 1A). Method H1 is extended to method H2 by considering two arbitrary angles *θ*_1_ = *π*/6 and *θ*_2_ = *π*/3 (Fig. 1B). The number of angles measured is then extended to eight angles in method H3, i.e. ${\theta }_{\mathrm{\{1,}\cdots \mathrm{,8\}}}$ from 0 to 5*π*/6 (Fig. 1C).

The estimated (denoted byˆ) longitudinal and transverse resistivity and reactivity ${\hat{\rho }}_{\{{\rm{L}},{\rm{T}}\}}$ and ${\hat{\tau }}_{\{{\rm{L}},{\rm{T}}\}}$ are represented by crosses (×) in the *y*-axis (*ϕ*_L_) and *x*-axis (*ϕ*_T_), respectively. To help the reader interpret the data, the estimated values represented by the crosses should appear as close as possible to the *true* values, i.e. *ρ*_{L,T}_ and *τ*_{L,T}_, the latter represented by dash-dot and dashed circumferences where intersect the longitudinal *ϕ*_L_ and transverse *ϕ*_T_ axes, respectively.

**Figure 2:**
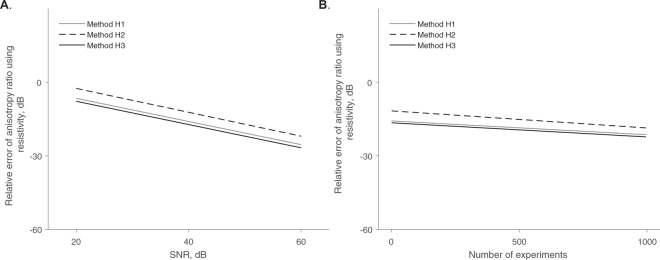
Comparison between methods H1 (solid grey line), H2 (dashed black line), and H3 (solid black line) estimating the resistivity anisotropy ratio ${\hat{\alpha }}^{2}$ while changing the signal-to-noise ratio (SNR) and keeping the number of measurements constant at *M* = 10 (**A**); and then while changing the number of measurements performed and keeping constant the SNR = 40 dB (**B**). Rates of decline of the relative error values between the estimated ${\hat{\alpha }}^{2}$ and true *α*^2^ resistivity anisotropy ratio value are graphically shown, calculated with a linear regression.

Note the accuracy of the aforementioned methods depends on both the SNR and *M*. To study the dependence of the precision of the methods based on these two parameters, we plot in Fig. 2A and B the accuracy of the resistivity anisotropy ratio while varying the SNR and the number of measurements *M*. The most accurate method is H3, then H1, and finally H2.

#### Benchmark results: simulation example of methods K2 and K3

**Figure 3:**
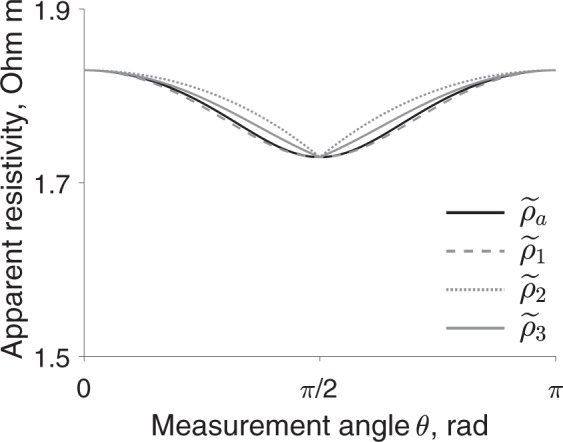
Apparent resistivity *ρ*_a_ = real(*κ*_a_) from (3) (solid black line) and the corresponding approximations ${\hat{\rho }}_{{\rm{a}}\mathrm{,1}}={\rm{real}}({\hat{\kappa }}_{{\rm{a}}\mathrm{,1}})$ in (8) (dashed grey line), ${\hat{\rho }}_{{\rm{a}}\mathrm{,2}}={\rm{real}}({\hat{\kappa }}_{{\rm{a}}\mathrm{,2}})$ in (9) (dotted grey line), ${\hat{\rho }}_{{\rm{a}}\mathrm{,3}}={\rm{real}}({\hat{\kappa }}_{{\rm{a}}\mathrm{,3}})$ in (10) (solid grey line). Simulation settings: aspect ratio *p* = 8.6, anisotropy ratio *α*^2^ = 0.5, measured frequency *f* = 10 kHz, transverse resistivity *ρ*_T_ = 2.93 Ω m.

**Figure 4:**
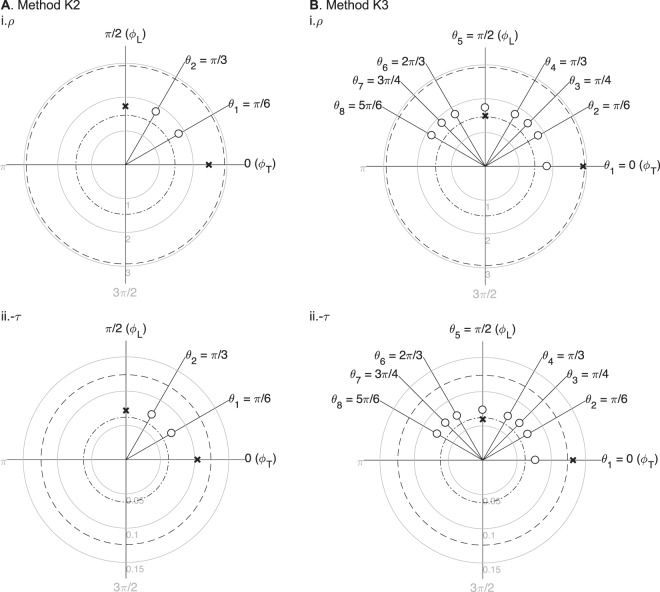
Numerical simulation results using methods K2 (**A**) and K3 (**B**). The estimated impedivity in longitudinal (L) and transverse (T) directions ${\hat{\kappa }}_{\{L,{\rm{T}}\}}$ (shown in crosses) were calculated from *M* = 10 averaged apparent impedivity ${\hat{\kappa }}_{{\rm{a}}}({\theta }_{d})$ measurements (shown in circles), with *d* = 1, …, *D* the measurement angles and interelectrodes’ distance *p* = 8.6. Method K2 (*D* = 2): ${\hat{\rho }}_{{\rm{L}}}=1.73$ Ω m, ${\hat{\rho }}_{{\rm{T}}}=2.47$ Ω m, ${\hat{\tau }}_{{\rm{L}}}=-\,0.07$ Ω m, ${\hat{\tau }}_{{\rm{T}}}=-\,0.10$ Ω m; Method K3 (*D* = 8): ${\hat{\rho }}_{{\rm{L}}}=1.49$ Ω m, ${\hat{\rho }}_{{\rm{T}}}=2.89$ Ω m, ${\hat{\tau }}_{{\rm{L}}}=-\,0.06$ Ω m, ${\hat{\tau }}_{{\rm{T}}}=-\,0.13$ Ω m. The true longitudinal and transverse resistivity (i) and reactivity (ii) are plotted in in dash-dot and dashed circumferences, *ρ*_L_ = 1.46 Ω m, *ρ*_T_ = 2.93 Ω m, *τ*_L_ = −0.06 Ω m and *τ*_T_ = −0.12 Ω m, respectively. The reactivity is shown −*τ* as a convention. The units of impedivity are Ω m.

**Figure 5:**
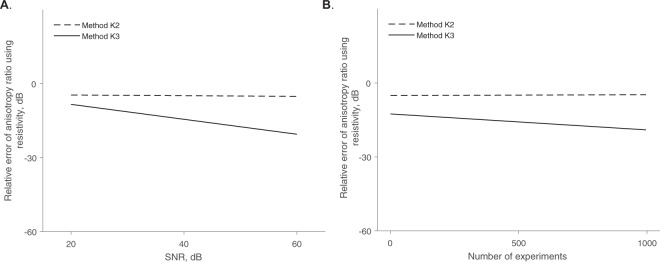
Comparison between methods K2 (dashed black line) and K3 (solid black line) estimating the resistivity anisotropy ratio ${\hat{\alpha }}^{2}$ while changing the signal-to-noise ratio (SNR) and keeping the number of measurements constant at *M* = 10 (**A**); and then while changing the number of measurements performed and keeping constant the SNR = 40 dB (**B**). Rates of decline of the relative error values between the estimated ${\hat{\alpha }}^{2}$ and true *α*^2^ resistivity anisotropy ratio value are graphically shown, calculated with a linear regression.

To evaluate the accuracy of the approximation, for example in (10), we calculate the relative error in ${\hat{\alpha }}^{2}$. The relative error in ${\hat{\alpha }}^{2}$ is 11.5% and 17.0% for methods K2 and K3. Note the accuracy of methods K2 and K3 will largely depend on the measurement angles *θ*_*d*_. When *θ*_*d*_ is close to 0 or *π*/2, the methods will provide more accurate estimates and vice versa (see Fig. 3). The estimated longitudinal and transverse resistivity and reactivity ${\hat{\rho }}_{\{{\rm{L}},{\rm{T}}\}}$ and ${\hat{\tau }}_{\{{\rm{L}},{\rm{T}}\}}$ are represented are shown in Fig. 4. Moreover, Fig. 5 shows that, unlike method K3, method K2 barely improves when the SNR or the number of measures increases.

#### Benchmark results: simulation example of methods B1 and B2

**Figure 6:**
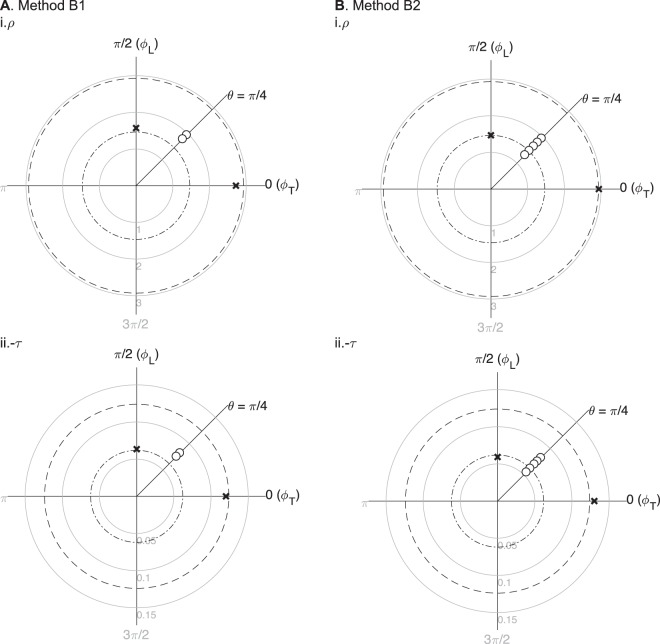
Numerical simulation results using methods B1 (**A**) and B2 (**B**). The estimated impedivity in longitudinal (L) and transverse (T) directions ${\hat{\kappa }}_{\{L,{\rm{T}}\}}$ (shown in crosses) were calculated from *M* = 10 averaged apparent impedivity ${\hat{\kappa }}_{{\rm{a}}}({p}_{n})$ measurements (shown in circles), with *n* = 1, …, *N* the number of inter-electrodes’ distances and *θ* = *π*/4 the measurement angle. Method B1 (*N* = 2): ${\hat{\rho }}_{{\rm{L}}}=1.57$ Ω m, ${\hat{\rho }}_{{\rm{T}}}=2.73$ Ω m, ${\hat{\tau }}_{{\rm{L}}}=-\,0.06$ Ω m, ${\hat{\tau }}_{{\rm{T}}}=-\,0.12$ Ω m; Method B2 (*N* = 5): ${\hat{\rho }}_{{\rm{L}}}=1.45$ Ω m and ${\hat{\rho }}_{{\rm{T}}}=2.94$ Ω m, ${\hat{\tau }}_{{\rm{L}}}=-\,0.06$ Ω m, ${\hat{\tau }}_{{\rm{T}}}=-\,0.13$ Ω m. The true longitudinal and transverse resistivity (i) and reactivity (ii) are plotted in dash-dot and dashed circumferences, *ρ*_L_ = 1.46 Ω m, *ρ*_T_ = 2.93 Ω m, *τ*_L_ = −0.06 Ω m and *τ*_T_ = −0.12 Ω m, respectively. The reactivity is shown −*τ* as a convention. The units of impedivity are Ω m.

**Figure 7:**
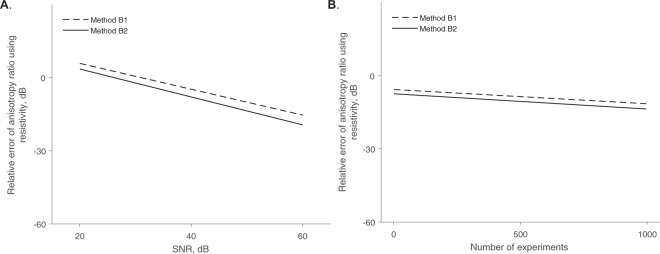
Comparison between methods B1 (dashed black line) and B2 (solid black line) estimating the resistivity anisotropy ratio ${\hat{\alpha }}^{2}$ while changing the signal-to-noise ratio (SNR) and keeping the number of measurements constant at *M* = 10 (**A**); and then while changing the number of measurements performed and keeping constant the SNR = 40 dB (**B**). Rates of decline of the relative error values between the estimated ${\hat{\alpha }}^{2}$ and true *α*^2^ resistivity anisotropy ratio value are graphically shown, calculated with a linear regression.

Figure 6 shows the results of methods B1 and B2 measuring apparent impedivity data with *N* = 2 (*p*_1_ = 20, *p*_2_ = 8.6) and *N* = 5 (*p*_1_ = 20, *p*_2_ = 8.6, *p*_3_ = 5.5, *p*_4_ = 4.0, *p*_5_ = 3.2) inter-electrodes’ distances and one measurement angle, respectively. To avoid confusion, we show the mean apparent resistivity and the mean aparent reactivity values (in circles) for each inter-electrodes’ distances slightly displaced on the same direction; otherwise, the circles would be superimposed one on top of the other, appearing with the naked eye as if there was one data point only. Figure 7 shows method B2 is more accurate than method B1.

### Experiment

**Figure 8:**
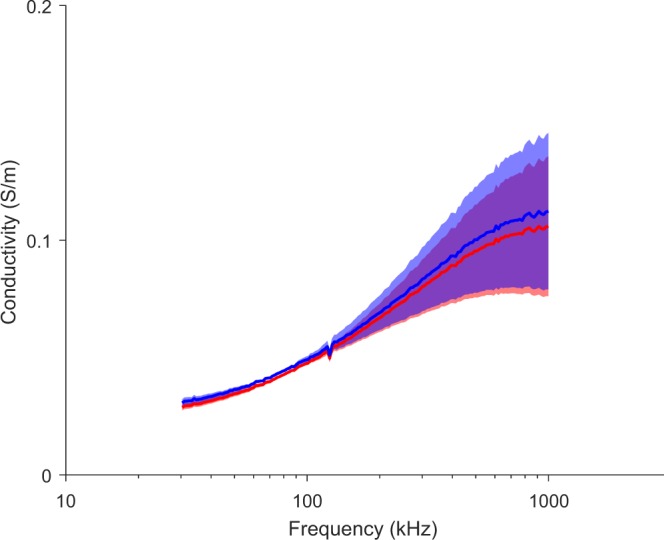
Estimated conductivity in longitudinal (blue) and transverse (red) direction. *In situ* mean ± standard deviation (*M* = 3 measurements).

**Figure 9:**
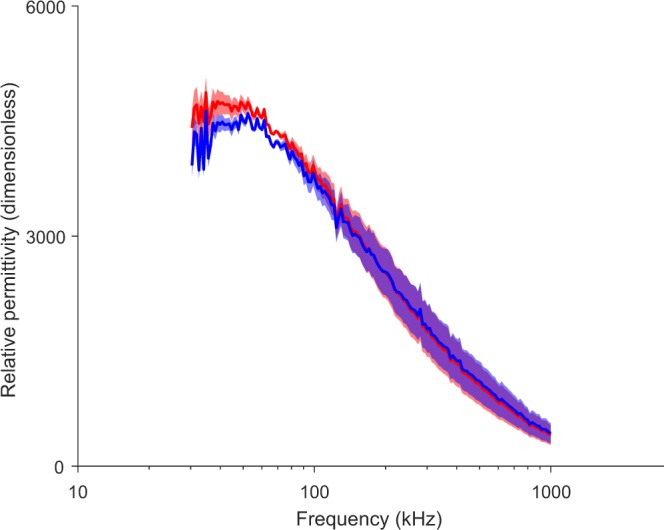
Estimated relative permittivity in longitudinal (blue) and transverse (red) direction. *In situ* mean ± standard deviation (*M* = 3 measurements).

Data measured *in situ* were estimated using method S1, the only method capable of measuring the permittivity regardless of the anisotropy direction. Data shown in Figs 8 and 9 are not intended for analytical purposes. Rather the goal is to show experimental feasibility and proof-of-concept measuring the permittivity of anisotropic muscle tissue *in situ* combining the method developed in this work with multipolar needles.

## Discussion

Numerous studies published over the last decades have reported the permittivity of biological fluids and tissues^20–22^. Having accurate methods to measure this physical property is fundamental to advance the development and research of medical technology^23^.

Despite the importance, even today there are still major gaps in the knowledge of the frequency-dependence of the permittivity of certain tissues. A review of the literature shows that actually only a few studies have been made on human skeletal muscle tissue^24,25^; in a few others, the anisotropic permittivity of muscle was measured in animals^26–28^. However, we are unaware of the existence of any study that has measured *in vivo* the permittivity of healthy or diseased muscle in patients.

Much of the reason for the paucity of studies measuring tissues’ anisotropic permittivity are the limitations of the methods associated with the existing measurement techniques. In the case of using the four-electrode impedance technique, one main limitation of the method reported by Rush is that prior knowledge is required concerning the direction of the tissue’s anisotropy^14^. The solution proposed by Rush has been widely used because of its simplicity; however, it is well-known it has limitations restricting its practical use^29^. For example, in animal experimentation, the direction of anisotropy can be approximated *in vivo* by visual inspection removing the skin and subcutaneous fat overlying the muscle but this is not possible in patients^30^. The second main limitation is that if the anisotropy direction is unknown (e.g. measuring muscle from outside by inserting the a four-electrode probe through the skin and subcutaneous fat tissues), we have shown that the minimum number of needle insertions needed to measure anisotropic permittivity is twelve, i.e. method C4 in Table 1^17^. This approach is valuable in pre-clinical studies; however, its clinical translation is questionable. Changing the experimental measurement from monopolar to multipolar needles and developing new S1 method, our study shows that it is possible to measure anisotropic permittivity while reducing the needle insertions required from twelve to six.

**Table 1:** Comparison of electrical impedance methods for the *in situ* measurement of anisotropic permittivity.

	Method	*D*	*N*	Exact model	Estimate anisotropy direction *ξ*	Unique result	Needle configuration	Number of needle insertions
Rush^14^	R1^†^	2	0	Yes	No	Yes	Monopolar	8
Kwon *et al*.^17^	R1^†^, C1^‡^	2	0	Yes	No	Yes	Monopolar	8
	R2^†^, C2^‡^	2	0	Yes	No	Yes	Monopolar	8
	R3^†^, C3^‡^	≥2	0	Yes	No	Yes	Monopolar	≥8
	C4^‡^	≥3	0	Yes	Yes	Yes	Monopolar	≥12
In this work	H1^‡^	2	1	Yes	No	Yes	Multipolar	4
	H2^‡^	2	1	Yes	No	No	Multipolar	4
	H3^‡^	≥2	1	Yes	No	No	Multipolar	≥4
	K2^‡^	2	1	No	No	No	Multipolar	4
	K3^‡^	≥2	1	No	No	No	Multipolar	≥4
	B1^‡^	1	2	Yes	No	Yes	Multipolar	2
	B2^‡^	1	≥2	Yes	No	Yes	Multipolar	2
	S1^‡^	≥3	≥2	Yes	Yes	Yes	Multipolar	≥6

Nomenclature: *D*, number of measurement angles; *N*, number of inter-electrodes’ distances within the same needle.

^†‡^Outcome: ^†^resistivity only; ^‡^resistivity and reactivity.

Since we are interested in muscle permittivity, the methods presented were developed for the measurement of materials with different anisotropies in two dimensions only. Developing methods accounting for the most general case of anisotropy in three dimensions is beyond the scope of the work presented. As for the dispersion in the experimental data, it can be attributed to several factors including the small sample size, biological variability, and the time elapsed between the death of the animals and the measurements. Still, the results are consistent with data available in the literature. For example, in canine skeletal muscle, the transverse conductivity has been reported from 0.04 to 0.10 S m^−1 ^^26,31^. There are other practical limitations affecting our methods, but these also apply to any other *in situ* method using needles^32,33^. For example, a fluid channel surrounding the electrodes will influence the data offering a less resistive path for the electrical current to flow. If the needles are very closely approximated to each other, the distribution of electrical current and thus the electric potential will be affected. Depending on the tissue being studied, if the electrodes are far apart from each other, the anisotropy direction may change. All these issues are possible sources of errors and they should be considered when performing experimental measurements.

Ultimately, we aim to measure muscles’ anisotropic permittivity *in vivo* to track disease progression and evaluate treatment effect in patients with neuromuscular diseases (NMD)^34,35^. In NMD, the pathophysiology of the disease alters the structure and composition of muscle tissue, which changes the permittivity of muscle and its anisotropy direction. For example, Duchenne muscular dystrophy is characterized by the loss of muscle fibers and their progressive substitution by fat and fibrous tissue. Improving our ability to measure with accuracy muscle permittivity can yield insight into developing new strategies for newer and more accurate diagnostic tools and treatments. This work is a step towards this direction and warrants further research to further reduce the number of needle insertions required to measure muscle permittivity *in vivo*.

## Materials

### Animals

Measurements were performed at the Animal Research Facility of Beth Israel Deaconess Medical Center in Boston, MA. This study did not require Institutional Animal Care and Use Committee approval because the animals were used for another experiment and measured here postmortem. We followed the same experimental procedure as our previous study^17^. *In situ* measurements on healthy skeletal muscle tissue from a freshly killed sheep (*n* = 3) were conducted at room temperature (25 °C) immediately after the animals were euthanized and completed within 1 hour postmortem. The skin and subcutaneous fat tissues were reflected back and the medius gluteus muscle exposed. Then, the multipolar needles were inserted into the muscle.

### Impedance measuring device

Ovine muscle impedance was measured using a commercial impedance analyzer (SFB7. Impedimed, Inc., Brisbane, Australia) using stepped-sine current excitation between 30.4 kHz and 1 MHz (155 frequencies total). For the positioning of multipolar needles we used a custom made matrix device^17^.

### Matrix device and multipolar needle

**Figure 10:**
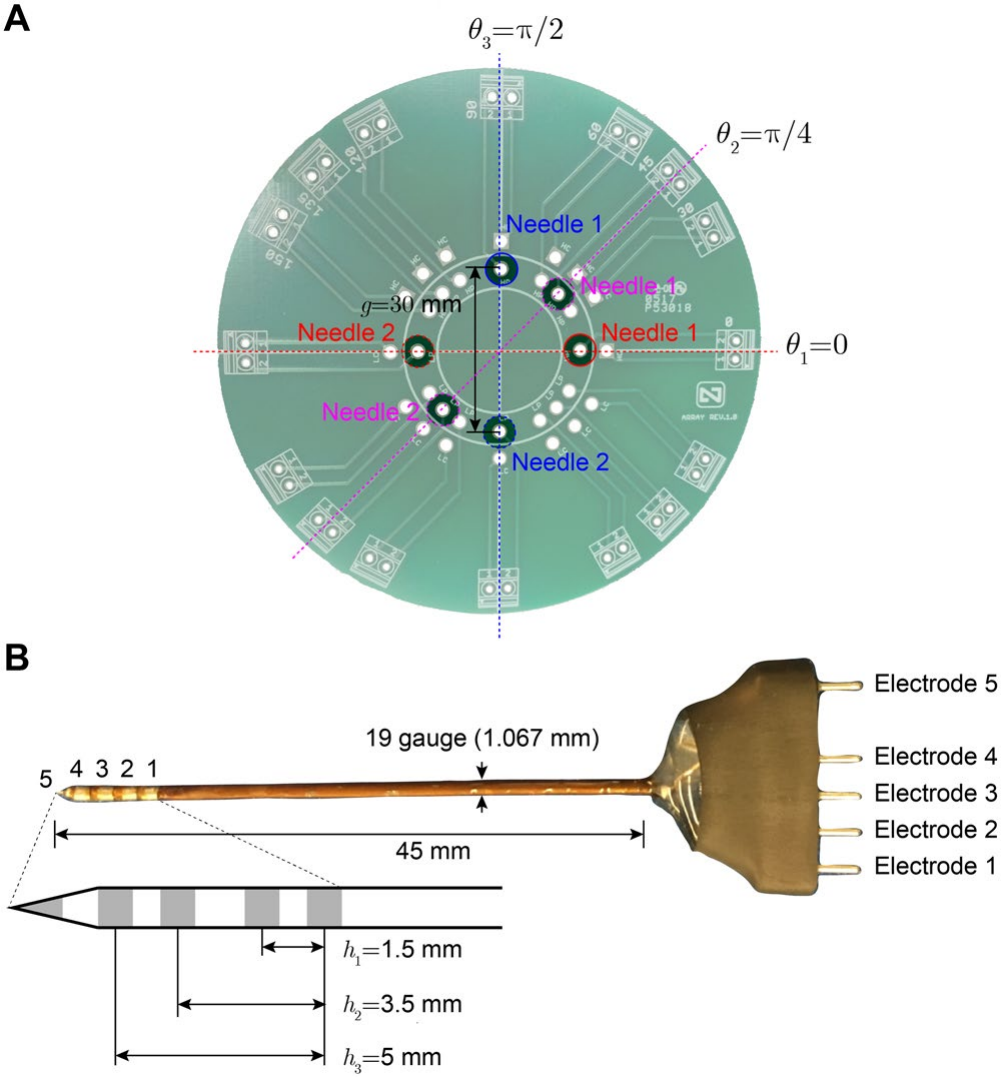
Printed circuit board (**A**)^17^ and multipolar needle (**B**)^36^ used for performing *in situ* measurements of ovine *ex vivo* muscle. The needles integrate a total of five electrodes, four electrodes arranged along the shaft of the needle, and a fifth, at the tip of the needle. Electrode 5 was not used in this study. The reader is referred to Table 2 for the measurement details.

The custom made printed circuit board (PCB) was already described in^17^ (Fig. 10A). Briefly, the PCB was positioned in contact with the surface of the muscle without considering any pre-specified direction. Then, two multipolar needles described in^36^ were inserted into the muscle tissue at a constant depth of 20 mm at predefined positions (Fig. 10B). Multi-frequency impedance data (number of measurements per angle *M* = 3) were then collected sequentially changing the electrodes’ configuration as detailed in Table 2. The aspect factors resulting from the separation between the needles *g* = 30 mm and the inter-electrodes’ distance *h*_1_ = 1.5 mm, *h*_2_ = 3.5 mm, and *h*_3_ = 5 mm, were *p*_1_ = 20, *p*_2_ = 8.6, *p*_3_ = 6. The reader is referred to Supplementary information, Part E for further information regarding the manufacturing of the needles.

**Table 2:** Experimental configuration employed for *in situ* experiments based on method S1.

	Needle	*p*_1_ = *g*/*h*_1_ = 20				*p*_2_ = *g*/*h*_2_ = 8.6				*p*_3_ = *g*/*h*_3_ = 6			
		Electrode				Electrode				Electrode			
		1	2	3	4	1	2	3	4	1	2	3	4
*θ*_1,2,3_ = 0,*π*/4,*π*/2	1	HC	LP	NC	NC	HC	NC	LP	NC	HC	NC	NC	LP
	2	HP	LC	NC	NC	HP	NC	LC	NC	HC	NC	NC	LC

We measured the apparent impedivity in *D* = 3 directions, i.e. *θ*_1,2,3_ = 0, *π*/4, *π*/2. For each direction, *N* = 3 inter-electrodes’ distances, i.e. *h*_1,2,3_ = 1.5, 3.5, 5 mm, were measured with the needle electrodes’ distance center to center being *g* = 30 mm. Electrodes’ abbreviation: HC, high (source) current; HP, high (positive) potential; LP, low (negative) potential; LC, low (sink) current; NC, not connected.

### Data analysis

Multi-frequency muscle impedance data were analyzed using method S1 using MATLAB. We calibrated apparent muscle impedance measured (resistance and reactance in Ω) into impedivity (resistivity and reactivity in Ω m) by performing impedance measurements in saline solution. We used the same experimental setup as in the *in situ* measurements. Calibration measurements were taken at room temperature immediately after immersing the needle electrode matrix into saline solution.

## Methods

### Terminology and definitions

The electric voltage $V\in {\mathbb{C}}$ (V) created by a current point-electrode in a (semi-)infinite, homogenous anisotropic tissue with anisotropicity in the *y*-axis and relative permittivity ${\varepsilon }_{r}\in {{\mathbb{R}}}_{ > 0}$ (dimensionless) is

1 $$V=\frac{{\kappa }_{\alpha }I}{K|{r}_{\alpha }|},$$

where *I* (A) is the applied current; ${\kappa }_{\alpha }\,:\,=\sqrt{{\kappa }_{{\rm{T}}}{\kappa }_{{\rm{L}}}}$ with ${\kappa }_{\{{\rm{L}},{\rm{T}}\}}={\rho }_{\{{\rm{L}},{\rm{T}}\}}+i{\tau }_{\{{\rm{L}},{\rm{T}}\}}$ (Ω m) the impedivity in longitudinal (L) and transverse (T) directions with *ρ* and *τ* the resistivity and the reactivity (Ω m), respectively; $i\,:\,=\sqrt{-1}$ is the imaginary unit (dimensionless); *K* = 2*π*, 4*π* (dimensionless) is a constant factor for semi-infinite and infinite domains, respectively; *r*_*α*_ = (*x*, *αy*, *z*) is the apparent position; the operator |**·**| is the *L*_2_ norm; and *α*^2^: = *ρ*_L_/*ρ*_T_ = *τ*_L_/*τ*_T_ (dimensionless) is the equal anisotropy ratio for the monodomain model. In the specific case in which the anisotropic tissues are considered purely resistive as in^14^ (i.e. *ε*_*r*_ = 0 and so *τ* = 0), (1) still holds with $V\in {\mathbb{R}}$ and where *κ*_*α*_ is replaced by the apparent resistivity ${\rho }_{\alpha }\,:\,=\sqrt{{\rho }_{{\rm{T}}}{\rho }_{{\rm{L}}}}$ (Ω m).

**Figure 11:**
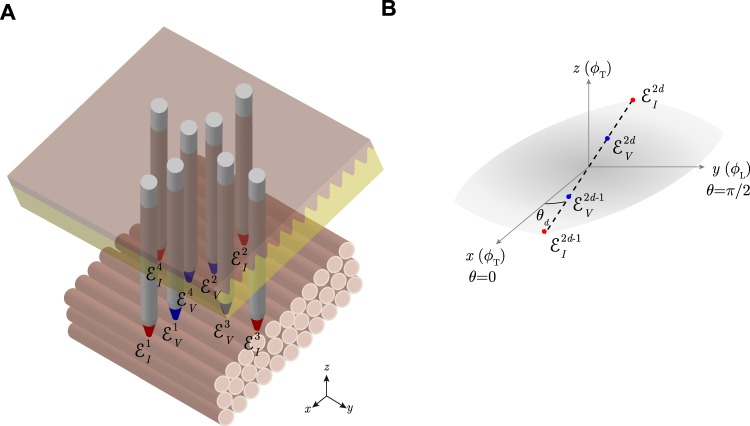
(**A**) Schematic showing the *in situ* measurement of anisotropic permittivity in *D* = 2 angles using *monopolar* needles^14,17^. Current and voltage electrodes are at the *same* depth, shown in red and blue color, respectively. (**B**) Schematic of the *d*-th angle measured in the *xy*-plane (*z* = 0). The measurement angle *θ*_*d*_ is defined with respect to the muscle fibers’ transverse (T) {*x*, *z*} direction *ϕ*_T_ (i.e. *ϕ*_T_ = *θ* when *θ* = 0) and *ϕ*_L_ is the longitudinal *y* direction. Electrode configuration: ${ {\mathcal E} }_{I}^{2d-1}$, source current electrode; ${ {\mathcal E} }_{V}^{2d-1}$, high potential electrode; ${ {\mathcal E} }_{V}^{2d}$, low potential electrode; ${ {\mathcal E} }_{I}^{2d}$, sink current electrode; where $d=\mathrm{1,}\,\mathrm{2,}\,\cdots ,\,D$ and *D* is the number of different angles measured from 0 to *π* with *θ*_*i*_ + *θ*_*j*_ ≠ *π* for $i,j\in \mathrm{\{1,}\,\mathrm{2,}\,\cdots ,\,D\}$ and *i* ≠ *j*.

Rush’s first work and our recent study^17^ are similar in that the methods developed to measure the complex permittivity *ε*: = *ε*′ + *iε*″ (dimensionless, henceforth referred simply as permittivity) of anisotropic tissues are based on (1) considering the *z*-component of the four-electrodes’ probe apparent position *r*_*α*_ is zero. In other words, the existing methods require the placement of the four-electrodes on the *xy*-plane (see Fig. 11). In doing so, the apparent impedivity (Ω m) measured using the principle of superposition in polar coordinates and the four-electrode technique reduces to

2 $${\kappa }_{{\rm{a}}}({\theta }_{d})=\frac{{\kappa }_{\alpha }}{\sqrt{{\cos }^{2}{\theta }_{d}+{\alpha }^{2}{\sin }^{2}{\theta }_{d}}}.$$

Then, from (2), the only possible way of obtaining anisotropy information on *ε* is by measuring the apparent impedivity *κ*_a_ in two or more different directions *θ* and computing *α* and *κ*_*α*_, where *θ* is defined as the measuring angle (in radians) with respect to the transverse direction *ϕ*_T_ determined by the tissues’ anisotropy.

In this paper, we extend our previous study by developing new methods that allow us to place the electrodes for measuring impedance at varying depths in the tissues by changing the *z* coordinate. In the same way as before, the ultimate goal of the methods is to estimate *κ*_{L,T}_; however, for notational convenience, the methods presented below are based on estimating *α*^2^ and *κ*_*α*_ instead. In the end, the reader can compute *κ*_{L,T}_ by simply using the following relationships

$${\kappa }_{{\rm{T}}}=\frac{{\kappa }_{\alpha }}{\alpha }\,{\rm{and}}\,{\kappa }_{{\rm{L}}}=\alpha {\kappa }_{\alpha }$$

(or its resistive analogue when considering *ε*_*r*_ = 0, i.e. replacing *κ* by *ρ*) and subsequently the permittivity

$${\varepsilon }_{\{{\rm{L}},{\rm{T}}\}}=\frac{1}{i\omega {\varepsilon }_{0}{\kappa }_{\{{\rm{L}},{\rm{T}}\}}}={\varepsilon }_{r,\{{\rm{L}},{\rm{T}}\}}-i\frac{{\sigma }_{\{{\rm{L}},{\rm{T}}\}}}{\omega {\varepsilon }_{0}},$$

with *ω* = 2*πf* (rad s^−1^) the (angular) frequency *f* (Hz) measured, *ε*_0_ (F m^−1^) the vacuum permittivity, *ε*_*r*_ the relative permittivity (dimensionless), and *σ* the conductivity (S m^−1^).

Henceforth, the naming convention of the methods presented is H, K, B, and S to distinguish from our previous work^17^.

### One inter-electrodes’ distance within the same needle and multiple measurement angles

**Figure 12:**
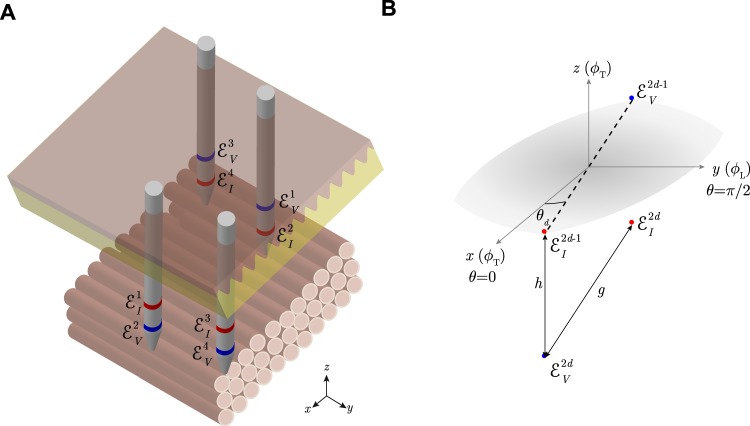
(**A**) Schematic showing the *in situ* measurement of anisotropic permittivity in *D* = 2 angles using *multipolar* needles. N.b. compared to Fig. 11, the pairs of current and voltage electrodes have different coordinates in the *z*-direction (in red and blue color, respectively). (**B**) Schematic of the *d*-th angle measured in the *xy*-plane (*z* = 0). The measurement angle *θ*_*d*_ is defined with respect to the muscle fibers’ transverse (T) {*x*, *z*} direction *ϕ*_T_ (i.e. *ϕ*_T_ = *θ* when *θ* = 0) and *ϕ*_L_ is the longitudinal *y* direction. Electrode configuration: ${ {\mathcal E} }_{I}^{2d-1}$, source current electrode; ${ {\mathcal E} }_{V}^{2d-1}$, high potential electrode; ${ {\mathcal E} }_{V}^{2d}$, low potential electrode; ${ {\mathcal E} }_{I}^{2d}$, sink current electrode; where $d=\mathrm{1,}\,\mathrm{2,}\,\cdots ,\,D$ is the number of different angles measured from 0 to *π*.

Let’s start considering the measurement setting shown in Fig. 12(A). According to Fig. 12(B), the apparent impedivity *κ*_a_ based on (1) is (see Theorem 1 in Supplementary information, Part A)

3 $${\kappa }_{{\rm{a}}}({\theta }_{d})={\kappa }_{\alpha }(1-\frac{1}{p\sqrt{{\cos }^{2}{\theta }_{d}+{\alpha }^{2}{\sin }^{2}{\theta }_{d}}}),$$

where $p\,:\,=g/h\in {{\mathbb{R}}}_{ > 0}$ (dimensionless) is the aspect ratio computed from *h* (m) the distance between electrodes in the *z* axis and *g* (m) the distance between electrodes in the same *xy* plane.

Below we provide three methods, from less to more general, to estimate the apparent impedivity ${\hat{\kappa }}_{\alpha }$ and the anisotropy ratio ${\hat{\alpha }}^{2}$ at a given measured frequency. First, the methods H1, H2 and H3 are developed based on (3). We then develop methods K2 and K3 after approximating (3). In both cases, the frequency dependence of the anisotropic impedivity can be computed iterating the methods presented at each particular frequency measured.

For consistency, we use the same nomenclature convention as in our previous work^17^. Noisy apparent impedivity data is denoted by the *m* superscript notation, i.e. ${\kappa }_{{\rm{a}}}^{[m]}({\theta }_{d})={\rho }_{{\rm{a}}}^{[m]}({\theta }_{d})+i{\tau }_{{\rm{a}}}^{[m]}({\theta }_{d})$. The measurement index is *m* and $M\in {\mathbb{N}}$ is the total number of measurements considered. Multiple measurements are performed sequentially for each measurement angle *θ*_*d*_ angles, where $d=\mathrm{1,}\cdots ,D$ and $D\in {{\mathbb{N}}}_{\ge 2}$ is the total number of angles measured. The averaged values within the experiment index *m* are denoted with the ˆ notation, i.e. ${\hat{\kappa }}_{{\rm{a}}}({\theta }_{d})\,:\,={\hat{\rho }}_{{\rm{a}}}({\theta }_{d})+i{\hat{\tau }}_{{\rm{a}}}({\theta }_{d})$.

#### Method H1: The apparent impedivity is measured in $D=2$ angles, i.e. ${\kappa }_{{\rm{a}}}^{[m]}({\theta }_{1})$ and ${\kappa }_{{\rm{a}}}^{[m]}({\theta }_{2})$, with ${\theta }_{1}\perp {\theta }_{2}$ and ${\theta }_{\{1,2\}}\in [0,\pi )$

Placing the electrodes in the anisotropic directions defined by the properties of the material is the simplest approach to estimate the anisotropy ratio ${\hat{\alpha }}^{2}$ and the geometric mean impedivity *κ*_*α*_ from (3) (see Theorem 2 in Supplementary information, Part B). Then,

4 $$\{\begin{array}{lll}{\hat{\kappa }}_{\alpha } & := & p{\hat{\kappa }}_{{\rm{a}}}\mathrm{(0)(}p-{\mathrm{1)}}^{-1}\\ {\hat{\alpha }}^{2} & := & {\hat{\rho }}_{{\rm{a}}}^{2}\mathrm{(0)(}p({\hat{\rho }}_{{\rm{a}}}\mathrm{(0)}-{\hat{\rho }}_{{\rm{a}}}(\pi \mathrm{/2))}+{\hat{\rho }}_{{\rm{a}}}(\pi {\mathrm{/2))}}^{-2}\\  & {\rm{or}} & {\hat{\tau }}_{{\rm{a}}}^{2}\mathrm{(0)(}p({\hat{\tau }}_{{\rm{a}}}\mathrm{(0)}-{\hat{\tau }}_{{\rm{a}}}(\pi \mathrm{/2))}+{\hat{\tau }}_{{\rm{a}}}(\pi {\mathrm{/2))}}^{-2}.\end{array}$$

The readers’ choice of using the apparent resistivity or the apparent reactivity to calculate ${\hat{\alpha }}^{2}$ in (4) should be based on the measurement noise. Note that Assumption 2 guarantees that the potential difference ${\rm{\Delta }}V\,:\,=V({ {\mathcal E} }_{V}^{2d})-V({ {\mathcal E} }_{V}^{2d-1})\ne 0$, where *d* ∈ {1, 2} corresponds to the measurement angle *θ*_*d*_ in the transverse direction *ϕ*_T_ determined by the tissues’ anisotropy. In other words, the apparent positions of the voltage measuring electrodes ${r}_{\alpha }({ {\mathcal E} }_{V}^{2d})$ and ${r}_{\alpha }({ {\mathcal E} }_{V}^{2d-1})$ are not on the same equipotential surface.

#### Method H2: The apparent impedivity is measured in $D=2$ angles, i.e. ${\kappa }_{{\rm{a}}}^{[m]}({\theta }_{1})$ and ${\kappa }_{{\rm{a}}}^{[m]}({\theta }_{2})$, and *θd* ∈ (0, π)

Method H1 can be generalized for any pair of angles. In this case, the geometric mean impedivity ${\hat{\kappa }}_{\alpha }$ can be estimated from the apparent impedivity values found after solving a 4-th degree polynomial ${P}_{4}({\hat{\kappa }}_{\alpha })\,:\,={\sum }_{n=0}^{4}{\lambda }_{n}{\hat{\kappa }}_{\alpha }^{n}$, where the coefficients are (see Theorem 3 in Supplementary information, Part B)

$$\begin{array}{lll}{\lambda }_{4} & := & {a}_{11}{a}_{22}-{a}_{12}{a}_{21},\\ {\lambda }_{3} & := & {a}_{11}{b}_{21}-{a}_{12}{a}_{23}+{a}_{13}{a}_{22}-{b}_{11}{a}_{21},\\ {\lambda }_{2} & := & -{a}_{11}{c}_{2}-{a}_{12}{b}_{22}+{a}_{13}{b}_{21}-{b}_{11}{a}_{23}+{b}_{12}{a}_{22}+{c}_{1}{a}_{21},\\ {\lambda }_{1} & := & -{a}_{13}{c}_{2}-{b}_{11}{b}_{22}+{b}_{12}{b}_{21}+{c}_{1}{a}_{23},\\ {\lambda }_{0} & := & {c}_{1}{b}_{22}-{b}_{12}{c}_{2},\end{array}$$

and

5 $$\begin{array}{c}{a}_{d1}\,:\,={p}^{2}{\sin }^{2}{\theta }_{d},\,{a}_{d2}\,:\,={p}^{2}{\cos }^{2}{\theta }_{d}-1,\,{a}_{d3}\,:\,=-\,2{p}^{2}{\sin }^{2}{\theta }_{d}{\hat{\kappa }}_{a}({\theta }_{d}),\\ {b}_{d1}\,:\,=-\,2{p}^{2}{\cos }^{2}{\theta }_{d}{\hat{\kappa }}_{a}({\theta }_{d}),\,{b}_{d2}\,:\,={p}^{2}{\sin }^{2}{\theta }_{d}{\hat{\kappa }}_{a}^{2}({\theta }_{d}),\,{c}_{d}\,:\,=-\,{p}^{2}{\cos }^{2}{\theta }_{d}{\hat{\kappa }}_{a}^{2}({\theta }_{d})\end{array}$$

for *d* = 1, 2. The analytic solution ${\hat{\kappa }}_{\alpha }$ to a 4-th degree equation exists. Then, we can estimate the anisotropy ratio ${\hat{\alpha }}^{2}$ as follows

6 $${\hat{\alpha }}^{2}\,:\,=-\,\frac{{a}_{22}{\hat{\kappa }}_{\alpha }^{2}+{b}_{21}{\hat{\kappa }}_{\alpha }-{c}_{2}}{{a}_{21}{\hat{\kappa }}_{\alpha }^{2}+{a}_{23}{\hat{\kappa }}_{\alpha }+{b}_{22}}.$$

Care should be taken when using the method above with *p* < 1. Note there exist some combination of *θ*_*d*_ and *α*^2^ that makes *κ*_a_ = 0 in (3), and *α*^2^ is *unknown*.

#### Method H3: The apparent impedivity is measured in *D* ≥ 2 angles, i.e. ${\kappa }_{{\rm{a}}}^{[m]}({\theta }_{d})$, and ${\theta }_{d}\in [\mathrm{0,}\,\pi )$

In general, for any number of measured angles *D* ≥ 2, the geometric mean impedivity ${\hat{\kappa }}_{\alpha }$ can be estimated by finding the roots of an 11-th degree polynomial ${P}_{11}({\hat{\kappa }}_{\alpha }):={\sum }_{n=0}^{11}{\mu }_{n}{\hat{\kappa }}_{\alpha }^{n}$. The coefficients *μ*_*n*_ do not have a compact expression and are therefore detailed in Theorem 4 in Supplementary information, Part B. Then, we can estimate anisotropy ratio ${\hat{\alpha }}^{2}$ as follows

7 $${\hat{\alpha }}^{2}\,:\,=-\,\frac{{{\bf{a}}}_{1}^{\top }{{\bf{a}}}_{2}{\hat{\kappa }}_{\alpha }^{4}+({{\bf{a}}}_{2}^{\top }{{\bf{a}}}_{3}+{{\bf{a}}}_{1}^{\top }{{\bf{b}}}_{1}){\hat{\kappa }}_{\alpha }^{3}+({{\bf{a}}}_{2}^{\top }{{\bf{b}}}_{2}+{{\bf{a}}}_{3}^{\top }{{\bf{b}}}_{1}-{{\bf{a}}}_{1}^{\top }{\bf{c}}){\hat{\kappa }}_{\alpha }^{2}+({{\bf{b}}}_{2}^{\top }{{\bf{b}}}_{1}-{{\bf{a}}}_{3}^{\top }{\bf{c}}){\hat{\kappa }}_{\alpha }-{{\bf{b}}}_{2}^{\top }{\bf{c}}}{{{\bf{a}}}_{1}^{\top }{{\bf{a}}}_{1}{\hat{\kappa }}_{\alpha }^{4}+2{{\bf{a}}}_{1}^{\top }{{\bf{a}}}_{3}{\hat{\kappa }}_{\alpha }^{3}+({{\bf{a}}}_{3}^{\top }{{\bf{a}}}_{3}+2{{\bf{a}}}_{1}^{\top }{{\bf{b}}}_{2}){\hat{\kappa }}_{\alpha }^{2}+2{{\bf{a}}}_{3}^{\top }{{\bf{b}}}_{2}{\hat{\kappa }}_{\alpha }+{{\bf{b}}}_{2}^{\top }{{\bf{b}}}_{2}}$$

where ${{\bf{a}}}_{1}\,:\,={[{a}_{11},{a}_{21},\cdots ,{a}_{D1}]}^{\top }$, ${{\bf{a}}}_{2}\,:\,={[{a}_{12},{a}_{22},\cdots ,{a}_{D2}]}^{\top }$, ${{\bf{a}}}_{3}\,:\,={[{a}_{13},{a}_{23},\cdots ,{a}_{D3}]}^{\top }$, ${{\bf{b}}}_{1}\,:\,={[{b}_{11},{b}_{21},\cdots ,{b}_{D1}]}^{\top }$, ${{\bf{b}}}_{2}\,:\,={[{b}_{12},{b}_{22},\cdots ,{b}_{D2}]}^{\top }$, ${\bf{c}}\,:\,={[{c}_{1},{c}_{2},\cdots ,{c}_{D}]}^{\top }$, where *a*_*d*1_, *a*_*d*2_, *a*_*d*3_, *b*_*d*1_, *b*_*d*2_, *c*_*d*_ are defined in (5) for $d=\mathrm{\{1,}\,\mathrm{2,}\,\cdots ,\,D\}$, and where $\top$ denotes the transpose operator.

#### Approximation

Applying methods H2 and H3 require finding the roots of a 4-th and 11-th degree polynomial, respectively. Therefore, there can be one or more physically meaningful solutions ${\hat{\kappa }}_{\alpha }$ within the 4 and 11 respective complex solutions. If so, choosing the right ${\hat{\kappa }}_{\alpha }$ may be challenging or impossible without a priori information. To decrease the order of the polynomial and therefore the number of possible solutions, we approximate square root in (3) as follows

$$\sqrt{{\cos }^{2}\theta +{\alpha }^{2}{\sin }^{2}\theta }\approx \phi (\theta )+\alpha \mathrm{(1}-\phi (\theta )),$$

where *φ*(*θ*) is a user-defined suitable function that does not depend on *α*, for example

8 $${\hat{\kappa }}_{{\rm{a}},1}\,:\,={\kappa }_{\alpha }(1-\frac{1}{p\mathrm{((1}-\alpha ){\cos }^{2}\theta +\alpha )})\,{\rm{with}}\,\phi (\theta )={\cos }^{2}\theta ,$$

9 $${\hat{\kappa }}_{{\rm{a}},2}\,:={\kappa }_{\alpha }(1-\frac{1}{p((1-\alpha )|\cos \theta |+\alpha )})\,{\rm{w}}{\rm{i}}{\rm{t}}{\rm{h}}\,\phi (\theta )=|\cos \theta |,$$

10 $${\hat{\kappa }}_{{\rm{a}},3}\,:={\kappa }_{\alpha }(1-\frac{1}{p((1-\alpha )({\cos }^{2}\theta +|\cos \theta |)/2+\alpha )})\,{\rm{w}}{\rm{i}}{\rm{t}}{\rm{h}}\,\phi (\theta )=\frac{1}{2}({\cos }^{2}\theta +|\cos \theta |).$$

and ${\hat{\kappa }}_{{\rm{a}},j}$ for *j* = 1, 2, 3 are suitable approximations (see Fig. 3) of the apparent impedivity in (3) (denoted by the notation~). The new approximated methods H2 and H3 based on (8–10) are named K2 and K3 (note method H1 does not require to be approximated since the estimation of ${\hat{\kappa }}_{\alpha }$ is unique).

#### Method K2: The apparent impedivity is measured in $D=2$ angles, i.e. ${\kappa }_{{\rm{a}}}^{[m]}({\theta }_{1})$ and ${\kappa }_{{\rm{a}}}^{[m]}({\theta }_{2})$

The anisotropy ratio ${\hat{\alpha }}^{2}$ can be estimated as from the apparent impedivity values measured in any combination of two angles (see Theorem 5 in Supplementary information, Part B) as follows

11 $${\hat{\alpha }}^{2}\,:\,={(\frac{{\mu }_{1}^{2}-2{\mu }_{0}{\mu }_{2}\mp {\mu }_{1}\sqrt{{\mu }_{1}^{2}-4{\mu }_{2}{\mu }_{0}}}{2{\mu }_{2}^{2}})}^{2}\,{\rm{or}}\,{(\frac{{\nu }_{1}^{2}-2{\nu }_{0}{\nu }_{2}\mp {\nu }_{1}\sqrt{{\nu }_{1}^{2}-4{\nu }_{2}{\nu }_{0}}}{2{\nu }_{2}^{2}})}^{2},$$

where *μ*_0_, *μ*_1_, *μ*_2_, *ν*_0_, *ν*_1_, *ν*_2_ are defined as

$$\{\begin{array}{lll}{\mu }_{2} & := & p\mathrm{(1}-\phi ({\theta }_{1}\mathrm{))(1}-\phi ({\theta }_{2}))({\hat{\rho }}_{{\rm{a}}}({\theta }_{1})-{\hat{\rho }}_{{\rm{a}}}({\theta }_{2}))\\ {\mu }_{1} & := & p({\hat{\rho }}_{{\rm{a}}}({\theta }_{1})-{\hat{\rho }}_{{\rm{a}}}({\theta }_{2}))(\phi ({\theta }_{1})+\phi ({\theta }_{2})-2\phi ({\theta }_{1})\phi ({\theta }_{2}))\\  &  & -({\hat{\rho }}_{{\rm{a}}}({\theta }_{1})-{\hat{\rho }}_{{\rm{a}}}({\theta }_{2}))+({\hat{\rho }}_{{\rm{a}}}({\theta }_{1})\phi ({\theta }_{1})-{\hat{\rho }}_{{\rm{a}}}({\theta }_{2})\phi ({\theta }_{2}))\\ {\mu }_{0} & := & p\phi ({\theta }_{1})\phi ({\theta }_{2})({\hat{\rho }}_{{\rm{a}}}({\theta }_{1})-{\hat{\rho }}_{{\rm{a}}}({\theta }_{2}))-({\hat{\rho }}_{{\rm{a}}}({\theta }_{1})\phi ({\theta }_{1})-{\hat{\rho }}_{{\rm{a}}}({\theta }_{2})\phi ({\theta }_{2}))\\ {\nu }_{2} & := & p\mathrm{(1}-\phi ({\theta }_{1}\mathrm{))(1}-\phi ({\theta }_{2}))({\hat{\tau }}_{{\rm{a}}}({\theta }_{1})-{\hat{\tau }}_{{\rm{a}}}({\theta }_{2}))\\ {\nu }_{1} & := & p({\hat{\tau }}_{{\rm{a}}}({\theta }_{1})-{\hat{\tau }}_{{\rm{a}}}({\theta }_{2}))(\phi ({\theta }_{1})+\phi ({\theta }_{2})-2\phi ({\theta }_{1})\phi ({\theta }_{2}))\\  &  & -({\hat{\tau }}_{{\rm{a}}}({\theta }_{1})-{\hat{\tau }}_{{\rm{a}}}({\theta }_{2}))+({\hat{\tau }}_{{\rm{a}}}({\theta }_{1})\phi ({\theta }_{1})-{\hat{\tau }}_{{\rm{a}}}({\theta }_{2})\phi ({\theta }_{2}))\\ {\nu }_{0} & := & p\phi ({\theta }_{1})\phi ({\theta }_{2})({\hat{\tau }}_{{\rm{a}}}({\theta }_{1})-{\hat{\tau }}_{{\rm{a}}}({\theta }_{2}))-({\hat{\tau }}_{{\rm{a}}}({\theta }_{1})\phi ({\theta }_{1})-{\hat{\tau }}_{{\rm{a}}}({\theta }_{2})\phi ({\theta }_{2}\mathrm{)).}\end{array}$$

The reader is referred to Theorem 5 in Supplementary information, Part B for the calculation of the geometric mean impedivity ${\hat{\kappa }}_{\alpha }$.

It is worth mentioning the above method can be less restrictive. For brevity, the details are available in Theorem 5 in Supplementary information, Part B.

#### Method K3: The apparent impedivity is measured in *D* ≥ 2 angles, i.e. ${\kappa }_{{\rm{a}}}^{[m]}({\theta }_{d})$ and ${\kappa }_{{\rm{a}}}^{[m]}({\theta }_{2})$, and ${\theta }_{d}\in [\mathrm{0,}\,\pi )$

There are three different scenarios to estimate the anisotropy ratio ${\hat{\alpha }}^{2}$ and the geometric mean impedivity ${\hat{\kappa }}_{\alpha }$ (see Theorem 6 in Supplementary information, Part B), enumerated below:

1. $${{\bf{a}}}_{{3}^{\perp }}\cdot {{\bf{a}}}_{1}\ne 0$$ and $${{\bf{a}}}_{{3}^{\perp }}\cdot {{\bf{a}}}_{2}\ne 0$$:
  $${\hat{\alpha }}^{2}\,:\,={(\frac{({{\bf{a}}}_{{3}^{\perp }}\cdot {{\bf{a}}}_{4})({{\bf{a}}}_{3}\cdot {{\bf{a}}}_{3})-({{\bf{a}}}_{{3}^{\perp }}\cdot {{\bf{a}}}_{1})({{\bf{a}}}_{3}\cdot {{\bf{a}}}_{2})+({{\bf{a}}}_{{3}^{\perp }}\cdot {{\bf{a}}}_{2})({{\bf{a}}}_{3}\cdot {{\bf{a}}}_{1})\pm \sqrt{T}}{2({{\bf{a}}}_{{3}^{\perp }}\cdot {{\bf{a}}}_{1})({{\bf{a}}}_{3}\cdot {{\bf{a}}}_{3})})}^{2}$$
  and
  12 $${\hat{\kappa }}_{\alpha }\,:\,=\frac{({{\bf{a}}}_{{3}^{\perp }}\cdot {{\bf{a}}}_{4})({{\bf{a}}}_{3}\cdot {{\bf{a}}}_{3})+({{\bf{a}}}_{{3}^{\perp }}\cdot {{\bf{a}}}_{1})({{\bf{a}}}_{3}\cdot {{\bf{a}}}_{2})-({{\bf{a}}}_{{3}^{\perp }}\cdot {{\bf{a}}}_{2})({{\bf{a}}}_{3}\cdot {{\bf{a}}}_{1})\mp \sqrt{T}}{\mathrm{2(}{{\bf{a}}}_{{3}^{\perp }}\cdot {{\bf{a}}}_{2})({{\bf{a}}}_{3}\cdot {{\bf{a}}}_{3})},$$
2. $${{\bf{a}}}_{{3}^{\perp }}\cdot {{\bf{a}}}_{1}=0$$ and $${{\bf{a}}}_{{3}^{\perp }}\cdot {{\bf{a}}}_{2}\ne 0$$:
  13 $${\hat{\alpha }}^{2}\,:\,={(\frac{({{\bf{a}}}_{{3}^{\perp }}\cdot {{\bf{a}}}_{2})({{\bf{a}}}_{3}\cdot {{\bf{a}}}_{4})-({{\bf{a}}}_{{3}^{\perp }}\cdot {{\bf{a}}}_{4})({{\bf{a}}}_{3}\cdot {{\bf{a}}}_{2})}{({{\bf{a}}}_{{3}^{\perp }}\cdot {{\bf{a}}}_{2})({{\bf{a}}}_{3}\cdot {{\bf{a}}}_{1})+({{\bf{a}}}_{{3}^{\perp }}\cdot {{\bf{a}}}_{4})({{\bf{a}}}_{3}\cdot {{\bf{a}}}_{3})})}^{2}\,{\rm{and}}\,{\hat{\kappa }}_{\alpha }\,:\,=\frac{{{\bf{a}}}_{{3}^{\perp }}\cdot {{\bf{a}}}_{4}}{{{\bf{a}}}_{{3}^{\perp }}\cdot {{\bf{a}}}_{2}},$$
3. $${{\bf{a}}}_{{3}^{\perp }}\cdot {{\bf{a}}}_{1}\ne 0$$, $${{\bf{a}}}_{{3}^{\perp }}\cdot {{\bf{a}}}_{2}=0$$:

14 $${\hat{\alpha }}^{2}\,:\,={(\frac{{{\bf{a}}}_{{3}^{\perp }}\cdot {{\bf{a}}}_{4}}{{{\bf{a}}}_{{3}^{\perp }}\cdot {{\bf{a}}}_{1}})}^{2}\,{\rm{and}}\,{\hat{\kappa }}_{\alpha }\,:\,=\frac{({{\bf{a}}}_{{3}^{\perp }}\cdot {{\bf{a}}}_{1})({{\bf{a}}}_{3}\cdot {{\bf{a}}}_{4})-({{\bf{a}}}_{{3}^{\perp }}\cdot {{\bf{a}}}_{4})({{\bf{a}}}_{3}\cdot {{\bf{a}}}_{1})}{({{\bf{a}}}_{{3}^{\perp }}\cdot {{\bf{a}}}_{1})({{\bf{a}}}_{3}\cdot {{\bf{a}}}_{2})+({{\bf{a}}}_{{3}^{\perp }}\cdot {{\bf{a}}}_{4})({{\bf{a}}}_{3}\cdot {{\bf{a}}}_{3})},$$

and ${{\bf{a}}}_{1}\,:\,={[{a}_{11},{a}_{21},\cdots ,{a}_{D1}]}^{\top }$, ${{\bf{a}}}_{2}\,:\,={[{a}_{12},{a}_{22},\cdots ,{a}_{D2}]}^{\top }$, ${{\bf{a}}}_{3}\,:\,={[{a}_{13},{a}_{23},\cdots ,{a}_{D3}]}^{\top }$, ${{\bf{a}}}_{4}\,:\,={[{a}_{14},{a}_{24},\cdots ,{a}_{D4}]}^{\top }$, ${a}_{d1}\,:\,=p{\hat{\kappa }}_{{\rm{a}}}({\theta }_{d})(\phi ({\theta }_{d})-1)$, *a*_*d*2_: = *pφ*(*θ*_*d*_) − 1, *a*_*d*3_: = −*p*(*φ*(*θ*_*d*_) − 1), ${a}_{d4}\,:\,=p{\hat{\kappa }}_{{\rm{a}}}({\theta }_{d})\phi ({\theta }_{d})$, for $d=\mathrm{1,}\,\mathrm{2,}\,\cdots ,\,D$, and $T={(({{\bf{a}}}_{{3}^{\perp }}\cdot {{\bf{a}}}_{4})({{\bf{a}}}_{3}\cdot {{\bf{a}}}_{3})+({{\bf{a}}}_{{3}^{\perp }}\cdot {{\bf{a}}}_{2})({{\bf{a}}}_{3}\cdot {{\bf{a}}}_{1})+({{\bf{a}}}_{{3}^{\perp }}\cdot {{\bf{a}}}_{1})({{\bf{a}}}_{3}\cdot {{\bf{a}}}_{2}))}^{2}\,-\,\mathrm{4(}{{\bf{a}}}_{{3}^{\perp }}\,\cdot \,{{\bf{a}}}_{1})({{\bf{a}}}_{{3}^{\perp }}\,\cdot \,{{\bf{a}}}_{2})(({{\bf{a}}}_{3}\,\cdot \,{{\bf{a}}}_{1})({{\bf{a}}}_{3}\,\cdot \,{{\bf{a}}}_{2})\,+\,({{\bf{a}}}_{3}\,\cdot \,{{\bf{a}}}_{3})({{\bf{a}}}_{3}\,\cdot \,{{\bf{a}}}_{4})),$ and ${{\bf{a}}}_{{3}^{\perp }}$ is perpendicular to **a**_3_.

### Multiple inter-electrodes’ distances within the same needle and one measurement angle

**Figure 13:**
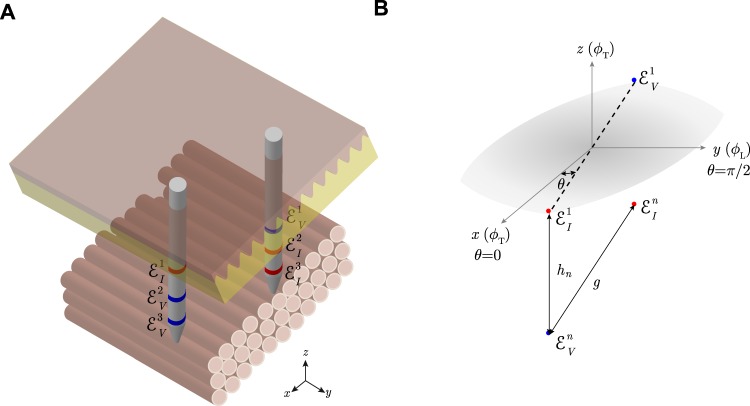
(**A**) Schematic showing the *in situ* measurement of anisotropic permittivity in *N* = 2 inter-electrodes’ distances using *multipolar* needles. N.b. compared to Fig. 12, there are more than one current and voltage electrodes in the *z*-direction within the same needle (in red and blue color, respectively). (**B**) Schematic of the *n*-th inter-electrodes’ distance measured in the *z*-direction. The measurement angle *θ* is defined with respect to the muscle fibers’ transverse (T) {*x*, *z*} direction *ϕ*_T_ (i.e. *ϕ*_T_ = *θ* when *θ* = 0) and *ϕ*_L_ is the longitudinal *y* direction. Electrode configuration: ${ {\mathcal E} }_{I}^{1}$, source current electrode; ${ {\mathcal E} }_{V}^{1}$, high potential electrode; ${ {\mathcal E} }_{V}^{n}$, low potential electrode; ${ {\mathcal E} }_{I}^{n}$, sink current electrode; where $n=\mathrm{2,}\,\mathrm{3,}\,\cdots ,\,N+1$ is the number of different inter-electrodes’ distances measured.

Now, instead of keeping the inter-electrodes’ distances fixed while changing the measurement orientation (Fig. 12), we consider the case where the measurement angle is unique *θ* and the inter-electrodes’ distances change *p*_*n*_ (Fig. 13A). According to Fig. 13(B), the apparent impedivity *κ*_a_ based on (1) is

15 $${\kappa }_{{\rm{a}}}({p}_{n})={\kappa }_{\alpha }(1-\frac{1}{{p}_{n}\sqrt{{\cos }^{2}\theta +{\alpha }^{2}{\sin }^{2}\theta }}).$$

#### Method B1: The apparent impedivity is measured in $N=2$ inter-electrodes’ distances, i.e. ${\kappa }_{{\rm{a}}}^{[m]}({p}_{1})$ and ${\kappa }_{{\rm{a}}}^{[m]}({p}_{2})$

The simplest case to estimate the geometric mean impedivity ${\hat{\kappa }}_{\alpha }$ and the anisotropy ratio ${\hat{\alpha }}^{2}$ is to measure the apparent impedivity with two different inter-electrodes’ distances (see Theorem 8 in Supplementary information, Part C), namely

16 $${\hat{\alpha }}^{2}\,:\,={({{\bf{q}}}^{\top }{\bf{q}})}^{-1}{{\bf{q}}}^{\top }{\bf{d}}\,{\rm{and}}\,{\hat{\kappa }}_{\alpha }\,:\,=\frac{{\hat{\kappa }}_{{\rm{a}}}({p}_{1}){p}_{1}-{\hat{\kappa }}_{{\rm{a}}}({p}_{2}){p}_{2}}{{p}_{1}-{p}_{2}},$$

where $${\bf{q}}\,:\,={[{p}_{1}^{2}{({\hat{\kappa }}_{\alpha }-{\hat{\kappa }}_{{\rm{a}}}({p}_{1}))}^{2}{\sin }^{2}\theta ,{p}_{2}^{2}{({\hat{\kappa }}_{\alpha }-{\hat{\kappa }}_{{\rm{a}}}({p}_{2}))}^{2}{\sin }^{2}\theta ]}^{\top }$$ and $${\bf{d}}\,:\,=[{\hat{\kappa }}_{\alpha }^{2}-{p}_{1}^{2}{({\hat{\kappa }}_{\alpha }-{\hat{\kappa }}_{{\rm{a}}}({p}_{1}))}^{2}{\cos }^{2}\theta ,\,{\hat{\kappa }}_{\alpha }^{2}-\,{p}_{2}^{2}$$$${({\hat{\kappa }}_{\alpha }-{\hat{\kappa }}_{{\rm{a}}}({p}_{2}))}^{2}{\cos }^{2}\theta ]\top $$, *p*_1_: = *g*/*h*_1_, and *p*_2_: = *g*/*h*_2_.

#### Method B2: The apparent impedivity is measured in *N* ≥ 2 inter-electrodes’ distances, i.e. ${\kappa }_{{\rm{a}}}^{[m]}({p}_{n})$

Method B2 is the generalization of method B1 when *N* ≥ 2 inter-electrodes’ distances (see Theorem 9 in Supplementary information, Part C). The geometric mean impedivity ${\hat{\kappa }}_{\alpha }$ and the anisotropy ratio ${\hat{\alpha }}^{2}$ can be estimated as follows

17 $${\hat{\alpha }}^{2}\,:\,=\frac{{x}^{2}-{y}^{2}{\cos }^{2}\theta }{{y}^{2}{\sin }^{2}\theta }\,{\rm{and}}\,{\hat{\kappa }}_{\alpha }:\,=x,$$

where $${[xy]}^{\top }\,:\,={({{\bf{B}}}^{\top }{\bf{B}})}^{-1}{{\bf{B}}}^{\top }{\bf{b}}$$, $${\bf{b}}\,:\,={[{\hat{\kappa }}_{{\rm{a}}}({p}_{1}){\hat{\kappa }}_{{\rm{a}}}({p}_{2})\cdots {\hat{\kappa }}_{{\rm{a}}}({p}_{N})]}^{\top }$$, and

$${\bf{B}}\,:\,=[\begin{array}{cc}1 & -\mathrm{1/}{p}_{1}\\ 1 & -\mathrm{1/}{p}_{2}\\ \vdots  & \vdots \\ 1 & -\mathrm{1/}{p}_{N}\end{array}].$$

### Multiple inter-electrodes’ distances within the same needle and multiple measurement angles

**Figure 14:**
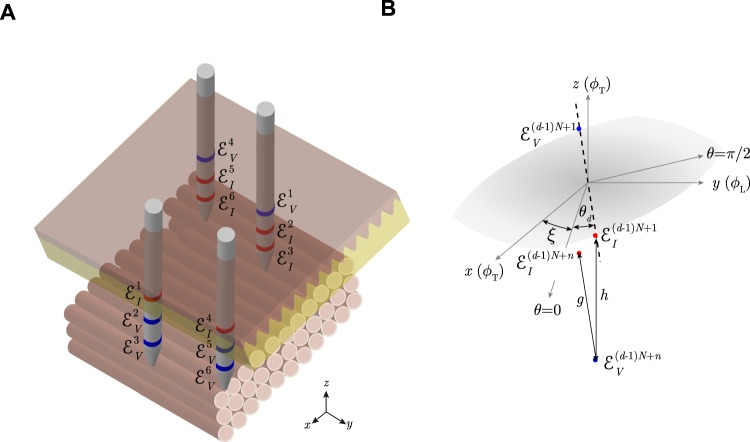
(**A**) Schematic showing the *in situ* measurement of anisotropic permittivity in *N* ≥ 2 inter-electrodes’ distances and *D* ≥ 3 measurement angles using *multipolar* needles. (**B**) Schematic of the *n*-th depth and *d*-th angle measured. The measurement angle *θ*_*d*_ is defined with respect to the *unknown* muscle fibers’ transverse (T) {*x*, *z*} direction *ϕ*_T_ modeled by *ξ* (*ϕ*_L_ is the longitudinal *y* direction). Electrode configuration: ${ {\mathcal E} }_{I}^{(d-\mathrm{1)}\cdot N+1}$, source current electrode; ${ {\mathcal E} }_{V}^{(d-\mathrm{1)}\cdot N+1}$, high potential electrode; ${ {\mathcal E} }_{V}^{(d-\mathrm{1)}\cdot N+n}$, low potential electrode; ${ {\mathcal E} }_{I}^{(d-\mathrm{1)}\cdot N+n}$, sink current electrode; where $n=\mathrm{2,}\,\mathrm{3,}\,\cdots ,\,N$ and $d=\mathrm{1,}\,\mathrm{2,}\,\cdots ,\,D$ are the number of different electrodes’ depth and measurement angles measured, respectively.

The reader may have noted that all previous methods require knowledge on the tissue’s anisotropic direction so that when *θ* = *π*/2 and *θ* = 0, the apparent impedivity measured is that corresponding to the anisotropic direction defined by the tissue, i.e. *θ* = *ϕ*_T_ when *θ* = 0 and consequently *θ* = *ϕ*_L_ when *θ* = *π*/2. Subsequently, experimental errors positioning the needle electrodes with respect the anisotropy in the permittivity of the tissue will give inaccurate estimates. We overcome this limitation by measuring the apparent impedivity with multiple electrodes’ depths and multiple measurement angles (see a sketch in Fig. 14A), namely

18 $${\kappa }_{{\rm{a}}}({\theta }_{d},{p}_{n})={\kappa }_{\alpha }(1-\frac{1}{{p}_{n}\sqrt{{\cos }^{2}({\theta }_{d}-\xi )+{\alpha }^{2}{\sin }^{2}({\theta }_{d}-\xi )}}),$$

where *ξ* is defined as the angle between the measurement axis and the true (unknown) anisotropy in the longitudinal and transverse permittivity of the tissue (see Fig. 14B).

#### Method S1: The apparent impedivity is measured in $N\ge 2$ inter-electrodes’ distances and $D\ge 3$ measurement angles, i.e. ${\kappa }_{{\rm{a}}}^{[m]}({\theta }_{d},{p}_{n})$

In this case, the geometric mean impedivity ${\hat{\kappa }}_{\alpha }$ can be estimated as follows (see Theorem 10 in Supplementary information, Part D),

19 $${\hat{\kappa }}_{\alpha }\,:\,=[1\,0\,0\,\cdots \,0]{({{\bf{S}}}^{\top }{\bf{S}})}^{-1}{{\bf{S}}}^{\top }{\bf{s}},$$

where ${\bf{s}}\,:\,={[{{\bf{s}}}_{1}{{\bf{s}}}_{2}\cdots {{\bf{s}}}_{D}]}^{\top }$ and ${{\bf{s}}}_{d}\,:\,=[{\hat{\kappa }}_{{\rm{a}}}({\theta }_{d},{p}_{1}\mathrm{)\ }{\hat{\kappa }}_{{\rm{a}}}({\theta }_{d},{p}_{2})\,\cdots \,{\hat{\kappa }}_{{\rm{a}}}({\theta }_{d},{p}_{N})]$ for $d=\mathrm{1,}\,\mathrm{2,}\,\cdots ,\,D$ and **S** is an augmented matrix of size *ND* × (*D* + 1) defined as

$${\bf{S}}\,:\,=[\begin{array}{c}{{\bf{S}}}_{1}\\ {{\bf{S}}}_{2}\\ \vdots \\ {{\bf{S}}}_{D}\end{array}]\,{\rm{with}}\,{{\bf{S}}}_{d}\,:\,=[{\bf{1}}\,{{\bf{p}}}_{d1}\,{{\bf{p}}}_{d2}\,\cdots \,{{\bf{p}}}_{dt}\,\cdots \,{{\bf{p}}}_{dD}]$$

a block matrix of size *N* × (*D* + 1) made of column vectors ${\bf{1}}\,:\,={[11\cdots 1]}^{\top }$ and ${{\bf{p}}}_{dt}\,:\,={\delta }_{d,t}{[-1/{p}_{1}-1/{p}_{2}\cdots -1/{p}_{N}]}^{\top }$ with *δ*_*d*,*t*_: = 1 if *t* = *d* else 0.

The estimated anisotropy ratio ${\hat{\alpha }}^{2}$ and anisotropy direction $\hat{\xi }$ can be estimated as follows

20 $${\hat{\alpha }}^{2}\,:\,=1-{\varpi }_{1}\,{\rm{or}}\,1-\sqrt{{\varpi }_{2}^{2}+{\varpi }_{3}^{2}}$$

and

21 $$\hat{\xi }\,:\,=\frac{1}{2}{\sin }^{-1}\frac{{\varpi }_{2}}{{\varpi }_{1}}\,{\rm{or}}\,\frac{1}{2}{\tan }^{-1}\frac{{\varpi }_{2}}{{\varpi }_{3}}\,{\rm{or}}\,\frac{1}{2}{\cos }^{-1}\frac{{\varpi }_{3}}{{\varpi }_{1}},$$

where $${[{\varpi }_{1}{\varpi }_{2}{\varpi }_{3}]}^{\top }\,:\,={({{\boldsymbol{\Xi }}}^{\top }{\boldsymbol{\Xi }})}^{-1}{{\boldsymbol{\Xi }}}^{\top }{\boldsymbol{\eta }}$$,

$${\boldsymbol{\Xi }}\,:\,=[\begin{array}{ccc}1 & -\,\sin \,2{\theta }_{1} & -\,\cos \,2{\theta }_{1}\\ 1 & -\,\sin \,2{\theta }_{2} & -\,\cos \,2{\theta }_{2}\\ \vdots  & \vdots  & \vdots \\ 1 & -\,\sin \,2{\theta }_{D} & -\,\cos \,2{\theta }_{D}\end{array}],\,{\boldsymbol{\eta }}\,:\,=[\begin{array}{c}2-\mathrm{2/}{q}_{1}\\ 2-\mathrm{2/}{q}_{2}\\ \vdots \\ 2-\mathrm{2/}{q}_{D}\end{array}],$$

and

$${q}_{d}\,:\,=\frac{1}{N}{\sum }_{n=1}^{N}{p}_{n}^{2}{(\frac{{\hat{\kappa }}_{{\rm{a}}}({\theta }_{d},{p}_{n})}{{\hat{\kappa }}_{\alpha }}-1)}^{2}.$$

## Supplementary information

Supplementary information

## Data Availability

All experimental data is available in the Supplementary Material.
