# Supplementary material for: New electrical impedance methods for the in situ measurement of the complex permittivity of anisotropic skeletal muscle using multipolar needles
Source: Sci Rep. 2019 Feb 28;9:3145. doi: 10.1038/s41598-019-39277-0 (PMC6395651; doi:10.1038/s41598-019-39277-0)
Supplement: Supplementary file 1 — Supplementary information [file 41598_2019_39277_MOESM1_ESM.pdf]

# Supplementary information for

## New electrical impedance methods for the in situ measurement of the complex permittivity of anisotropic skeletal muscle using multipolar needles

**H Kwon, M Guasch, J Nagy, S B Rutkove and B Sanchez**

Department of Neurology, Beth Israel Deaconess Medical Center, Harvard Medical School, Boston, MA 02215-5491, USA.

E-mail: `bsanchez@bidmc.harvard.edu` and `hyeuknamkwon@gmail.com`

This PDF file includes Supplementary Parts A, B, C, D and E

## Part A. Mathematical background

**Theorem 1.** *We assume an infinite homogeneous anisotropic material with impedivity* $\kappa = \rho + i\tau$ *and same anisotropy ratio* $\alpha^2$ *on the resistivity* $\rho$ *and the reactivity* $\tau$*, i.e.* $\alpha^2 := \rho_\mathrm{L}/\rho_\mathrm{T} = \tau_\mathrm{L}/\tau_\mathrm{T} < 1$*, where the subscript* $T$ *denotes the transverse direction on the* $x$*- and* $z$*-axis and the subscript* $L$ *denotes the longitudinal direction on the* $y$*-axis. Consider the positive (source)* $\mathcal{E}_{I+}$ *and negative (sink)* $\mathcal{E}_{I-}$ *current electrodes are placed in* $(g/2\cos\theta, g/2\sin\theta, 0)$ *and* $(-g/2\cos\theta, -g/2\sin\theta, -h)$*, respectively, while the positive (high)* $\mathcal{E}_{V+}$ *and negative (low)* $\mathcal{E}_{V-}$ *voltage electrodes are placed in* $(g/2\cos\theta, g/2\sin\theta, -h)$ *and* $(-g/2\cos\theta, -g/2\sin\theta, 0)$*, respectively, with* $\theta \in [0, \pi)$*. Here,* $g$ *is defined as the distance between electrodes on the same* $xy$*-plane whereas* $h$ *is defined as the distance between the electrodes with same* $xy$*-position. Then, the apparent impedivity* $\kappa_\mathrm{a}$ *is*

$$\kappa_\mathrm{a} = \kappa_\alpha \left(1 - \frac{1}{p\sqrt{\cos^2\theta + \alpha^2 \sin^2\theta}}\right), \tag{1}$$

*where* $p := g/h$ *is the aspect ratio and* $\kappa_\alpha := \sqrt{\kappa_\mathrm{T}\kappa_\mathrm{L}}$ *is the geometric mean impedivity.*

*Proof.* The electric potential $V$ (V) generated by point current source with amplitude $I$ (A) is (Kwon et al. 2017)

$$V = \frac{\kappa_\alpha I}{K|r_\alpha|},$$

where $K = 4\pi$ (dimensionless) and $r_\alpha = (x, \alpha y, z)$ is the apparent position. Then the measured electric potential $V_+$ at $\mathcal{E}_{V+}$ induced by the current electrodes $\mathcal{E}_{I+}$ and $\mathcal{E}_{I-}$ is

$$V_+ = \frac{\kappa_\alpha I}{Kh} - \frac{\kappa_\alpha I}{Kg\sqrt{\cos^2\theta + \alpha^2\sin^2\theta}}.$$

In the same way, the electric potential $V_-$ at $\mathcal{E}_{V-}$ induced by the current electrodes $\mathcal{E}_{I+}$ and $\mathcal{E}_{I-}$ is $V_- = -V_+$. Therefore, the measured impedance $Z$ is

$$Z = \frac{V_+ - V_-}{I} = \frac{2\kappa_\alpha}{hK}\left(1 - \frac{1}{p\sqrt{\cos^2\theta + \alpha^2\sin^2\theta}}\right),$$

where $p := g/h$ (dimensionless). Finally, we define the apparent impedivity $\kappa_\mathrm{a}$ as follows

$$\kappa_\mathrm{a} := \frac{hKZ}{2} = \kappa_\alpha\left(1 - \frac{1}{p\sqrt{\cos^2\theta + \alpha^2\sin^2\theta}}\right).$$

□

## Part B. One inter-electrodes' distance within the same needle and multiple measurement angles

**Theorem 2** (Method H1)**.** *Let* $\phi_\mathrm{L} = \pi/2$ *and* $\phi_\mathrm{T} = 0$ *be the angles of the longitudinal (L) and transverse (T) of the anisotropic complex permittivity, respectively. Then, the*

*geometric mean impedivity* $\kappa_\alpha$ *and the square root of the anisotropy ratio* $\alpha$ *can be calculated from the apparent impedivity* $\kappa_{\rm a}(\theta_1)$ *and* $\kappa_{\rm a}(\theta_2)$ *for* $\theta_1 = 0$ *and* $\theta_2 = \pi/2$ *and* $p \in \mathbb{R}_{>0}$ *as follows*

$$\begin{cases} \kappa_\alpha &= p\kappa_{\rm a}(0)(p-1)^{-1} \\ \alpha^2 &= \kappa_{\rm a}^2(0)(p(\kappa_{\rm a}(0)-\kappa_{\rm a}(\pi/2))+\kappa_{\rm a}(\pi/2))^{-2}. \end{cases}$$

*Proof.* We have from (1) when $\theta = 0, \pi/2$

$$\begin{cases} \kappa_{\rm a}(0) &= \kappa_\alpha\left(1-\frac{1}{p}\right) \\ \kappa_{\rm a}(\pi/2) &= \kappa_\alpha\left(1-\frac{1}{p\alpha}\right). \end{cases} \tag{2}$$

From the first equation above, one can find that

$$\kappa_\alpha = \frac{p\kappa_{\rm a}(0)}{p-1}.$$

To calculate $\alpha$, we first separate the resistivity and reactivity parts of (2),

$$\begin{cases} \rho_{\rm a}(0) &= \rho_\alpha\left(1-\frac{1}{p}\right) \\ \rho_{\rm a}(\pi/2) &= \rho_\alpha\left(1-\frac{1}{p\alpha}\right) \end{cases} \quad \text{and} \quad \begin{cases} \tau_{\rm a}(0) &= \tau_\alpha\left(1-\frac{1}{p}\right) \\ \tau_{\rm a}(\pi/2) &= \tau_\alpha\left(1-\frac{1}{p\alpha}\right). \end{cases}$$

Dividing the first and second equation gives

$$\frac{\rho_{\rm a}(0)}{\rho_{\rm a}(\pi/2)} = \frac{p\alpha-\alpha}{p\alpha-1} \quad \text{and} \quad \frac{\tau_{\rm a}(0)}{\tau_{\rm a}(\pi/2)} = \frac{p\alpha-\alpha}{p\alpha-1}.$$

Rearranging the previous equation one can find the anisotropy ratio, namely

$$\alpha^2 = \left(\frac{\rho_{\rm a}(0)}{p(\rho_{\rm a}(0)-\rho_{\rm a}(\pi/2))+\rho_{\rm a}(\pi/2)}\right)^2 = \left(\frac{\tau_{\rm a}(0)}{p(\tau_{\rm a}(0)-\tau_{\rm a}(\pi/2))+\tau_{\rm a}(\pi/2)}\right)^2.$$

□

**Theorem 3** (Method H2)**.** *Let* $\theta_1,\ \theta_2 \in [0,\pi)$ *two measurement angles that satisfy* $\cos^2\theta_1 \neq \cos^2\theta_2$ *and* $p \in \mathbb{R}_{>0}$*. Then, the geometric mean impedivity* $\kappa_\alpha$ *satisfies the following 4th degree polynomial*

$$\begin{aligned} &(a_{11}a_{22}-a_{12}a_{21})x^4 + (a_{11}b_{21}-a_{12}a_{23}+a_{13}a_{22}-b_{11}a_{21})x^3 \\ &-(a_{11}c_2+a_{12}b_{22}-a_{13}b_{21}+b_{11}a_{23}-b_{12}a_{22}-c_1a_{21})x^2 \\ &-(a_{13}c_2+b_{11}b_{22}-b_{12}b_{21}-c_1a_{23})x-(b_{12}c_2-c_1b_{22}) = 0, \end{aligned} \tag{3}$$

*where*

$$\begin{aligned} a_{d1} &:= p^2\sin^2\theta_d, & a_{d2} &:= p^2\cos^2\theta_d - 1, & a_{d3} &:= -2p^2\sin^2\theta_d\kappa_{\rm a}(\theta_d), \\ b_{d1} &:= -2p^2\cos^2\theta_d\kappa_{\rm a}(\theta_d), & b_{d2} &:= p^2\sin^2\theta_d\kappa_{\rm a}^2(\theta_d), & c_d &:= -p^2\cos^2\theta_d\kappa_{\rm a}^2(\theta_d) \end{aligned} \tag{4}$$

*for* $d = 1, 2$*. Then, the anisotropy ratio* $\alpha^2$ *is*

$$\alpha^2 = -\frac{a_{22}\kappa_\alpha^2 + b_{21}\kappa_\alpha - c_2}{a_{21}\kappa_\alpha^2 + a_{23}\kappa_\alpha + b_{22}}.$$

*Proof.* From (1), we have

$$\begin{aligned} a_{11}x^2y + a_{12}x^2 + a_{13}xy + b_{11}x + b_{12}y &= c_1 \\ a_{21}x^2y + a_{22}x^2 + a_{23}xy + b_{21}x + b_{22}y &= c_2. \end{aligned} \tag{5}$$

From the second equation in (5),

$$y = -\frac{a_{22}x^2 + b_{21}x - c_2}{a_{21}x^2 + a_{23}x + b_{22}}. \tag{6}$$

Inserting (6) into the first equation of (5) gives

$$\begin{aligned} &(a_{11}a_{22} - a_{12}a_{21})x^4 + (a_{11}b_{21} - a_{12}a_{23} + a_{13}a_{22} - b_{11}a_{21})x^3 \\ &-(a_{11}c_2 + a_{12}b_{22} - a_{13}b_{21} + b_{11}a_{23} - b_{12}a_{22} - c_1a_{21})x^2 \\ &-(a_{13}c_2 + b_{11}b_{22} - b_{12}b_{21} - c_1a_{23})x - (b_{12}c_2 - c_1b_{22}) = 0. \end{aligned}$$

which equals (3). Then, the mean geometric impedivity $\kappa_\alpha$ satisfies the equation above. The anisotropy ratio $\alpha^2$ follows from (6) where $y := \alpha^2$ and $x := \kappa_\alpha$.

Note that in the case when $\cos^2\theta_1 = \cos^2\theta_2$ for $\theta_1, \theta_2 \in [0, \pi)$, then $\sin^2\theta_1 = \sin^2\theta_2$, which implies

$$\begin{aligned} a_{11} = a_{21}, \quad a_{12} = a_{22}, \quad a_{13} = a_{23}, \\ b_{11} = b_{21}, \quad b_{12} = b_{22}, \quad c_1 = c_2 \end{aligned}$$

and so all the coefficients in (3) become zero giving a trivial equation. □

**Theorem 4** (Method H3). *Let $\theta_d \in [0,\ \pi)$ for $d = 1, \ldots, D$, $D \in \mathbb{N}_{\geq 2}$ and $p \in \mathbb{R}_{>0}$. Then, the geometric mean impedivity $\kappa_\alpha$ satisfies the following 11th degree polynomial*

$$\sum_{n=0}^{11} \lambda_n x^n, \tag{7}$$

*where the coefficients $\lambda_n$ for $n = 0, 1, \ldots, 11$ are*

$$
\begin{aligned}
\lambda_{11} &= 2(\mathbf{a}_1^\top\mathbf{a}_1)((\mathbf{a}_1^\top\mathbf{a}_1)(\mathbf{a}_2^\top\mathbf{a}_2) - (\mathbf{a}_1^\top\mathbf{a}_2)^2),\\
\lambda_{10} &= 3(\mathbf{a}_1^\top\mathbf{a}_1)^2(\mathbf{a}_2^\top\mathbf{b}_1) - 5(\mathbf{a}_1^\top\mathbf{a}_2)^2(\mathbf{a}_1^\top\mathbf{a}_3) - 3(\mathbf{a}_1^\top\mathbf{a}_1)(\mathbf{a}_1^\top\mathbf{a}_2)(\mathbf{a}_2^\top\mathbf{a}_3) + 8(\mathbf{a}_1^\top\mathbf{a}_1)(\mathbf{a}_1^\top\mathbf{a}_3)(\mathbf{a}_2^\top\mathbf{a}_2) - 3(\mathbf{a}_1^\top\mathbf{a}_1)(\mathbf{a}_1^\top\mathbf{a}_2)(\mathbf{a}_1^\top\mathbf{b}_1),\\
\lambda_9 &= 8(\mathbf{a}_1^\top\mathbf{a}_3)^2(\mathbf{a}_2^\top\mathbf{a}_2) - (\mathbf{a}_1^\top\mathbf{a}_1)(\mathbf{a}_2^\top\mathbf{a}_3)^2 - 3(\mathbf{a}_1^\top\mathbf{a}_2)^2(\mathbf{a}_3^\top\mathbf{a}_3) - (\mathbf{a}_1^\top\mathbf{a}_1)(\mathbf{a}_1^\top\mathbf{b}_1)^2 - 6(\mathbf{a}_1^\top\mathbf{a}_2)^2(\mathbf{a}_1^\top\mathbf{b}_2) - 2(\mathbf{a}_1^\top\mathbf{a}_1)^2(\mathbf{a}_2^\top\mathbf{c}) + (\mathbf{a}_1^\top\mathbf{a}_1)^2(\mathbf{b}_1^\top\mathbf{b}_1)\\
&\quad -8(\mathbf{a}_1^\top\mathbf{a}_2)(\mathbf{a}_1^\top\mathbf{a}_3)(\mathbf{a}_2^\top\mathbf{a}_3) + 4(\mathbf{a}_1^\top\mathbf{a}_1)(\mathbf{a}_2^\top\mathbf{a}_2)(\mathbf{a}_3^\top\mathbf{a}_3) - 8(\mathbf{a}_1^\top\mathbf{a}_2)(\mathbf{a}_1^\top\mathbf{a}_3)(\mathbf{a}_1^\top\mathbf{b}_1) - 2(\mathbf{a}_1^\top\mathbf{a}_1)(\mathbf{a}_1^\top\mathbf{a}_2)(\mathbf{a}_2^\top\mathbf{b}_2) + 12(\mathbf{a}_1^\top\mathbf{a}_1)(\mathbf{a}_1^\top\mathbf{a}_3)(\mathbf{a}_2^\top\mathbf{b}_1)\\
&\quad +8(\mathbf{a}_1^\top\mathbf{a}_1)(\mathbf{a}_2^\top\mathbf{a}_2)(\mathbf{a}_1^\top\mathbf{b}_2) - 2(\mathbf{a}_1^\top\mathbf{a}_1)(\mathbf{a}_2^\top\mathbf{a}_3)(\mathbf{a}_1^\top\mathbf{b}_1) - 2(\mathbf{a}_1^\top\mathbf{a}_1)(\mathbf{a}_1^\top\mathbf{a}_2)(\mathbf{a}_3^\top\mathbf{b}_1) + 2(\mathbf{a}_1^\top\mathbf{a}_1)(\mathbf{a}_1^\top\mathbf{a}_2)(\mathbf{a}_1^\top\mathbf{c}),\\
\lambda_8 &= 12(\mathbf{a}_1^\top\mathbf{a}_3)^2(\mathbf{a}_2^\top\mathbf{b}_1) - 3(\mathbf{a}_1^\top\mathbf{a}_3)(\mathbf{a}_1^\top\mathbf{b}_1)^2 - 3(\mathbf{a}_1^\top\mathbf{a}_3)(\mathbf{a}_2^\top\mathbf{a}_3)^2 - 7(\mathbf{a}_1^\top\mathbf{a}_2)^2(\mathbf{a}_3^\top\mathbf{b}_2) - (\mathbf{a}_1^\top\mathbf{a}_1)^2(\mathbf{b}_1^\top\mathbf{c}) - 5(\mathbf{a}_1^\top\mathbf{a}_2)(\mathbf{a}_2^\top\mathbf{a}_3)(\mathbf{a}_3^\top\mathbf{a}_3) + 8(\mathbf{a}_1^\top\mathbf{a}_3)(\mathbf{a}_2^\top\mathbf{a}_2)(\mathbf{a}_3^\top\mathbf{a}_3)\\
&\quad -6(\mathbf{a}_1^\top\mathbf{a}_2)(\mathbf{a}_1^\top\mathbf{a}_3)(\mathbf{a}_2^\top\mathbf{b}_2) - 10(\mathbf{a}_1^\top\mathbf{a}_2)(\mathbf{a}_2^\top\mathbf{a}_3)(\mathbf{a}_1^\top\mathbf{b}_2) + 16(\mathbf{a}_1^\top\mathbf{a}_3)(\mathbf{a}_2^\top\mathbf{a}_2)(\mathbf{a}_1^\top\mathbf{b}_2) - 6(\mathbf{a}_1^\top\mathbf{a}_3)(\mathbf{a}_2^\top\mathbf{a}_3)(\mathbf{a}_1^\top\mathbf{b}_1) - (\mathbf{a}_1^\top\mathbf{a}_1)(\mathbf{a}_2^\top\mathbf{a}_3)(\mathbf{a}_2^\top\mathbf{b}_2)\\
&\quad -6(\mathbf{a}_1^\top\mathbf{a}_2)(\mathbf{a}_1^\top\mathbf{a}_3)(\mathbf{a}_3^\top\mathbf{b}_1) - 5(\mathbf{a}_1^\top\mathbf{a}_2)(\mathbf{a}_3^\top\mathbf{a}_3)(\mathbf{a}_1^\top\mathbf{b}_1) + 8(\mathbf{a}_1^\top\mathbf{a}_1)(\mathbf{a}_2^\top\mathbf{a}_2)(\mathbf{a}_3^\top\mathbf{b}_2) - (\mathbf{a}_1^\top\mathbf{a}_1)(\mathbf{a}_2^\top\mathbf{a}_3)(\mathbf{a}_3^\top\mathbf{b}_1) + 6(\mathbf{a}_1^\top\mathbf{a}_1)(\mathbf{a}_3^\top\mathbf{a}_3)(\mathbf{a}_2^\top\mathbf{b}_1)\\
&\quad -10(\mathbf{a}_1^\top\mathbf{a}_2)(\mathbf{a}_1^\top\mathbf{b}_1)(\mathbf{a}_1^\top\mathbf{b}_2) + 6(\mathbf{a}_1^\top\mathbf{a}_2)(\mathbf{a}_1^\top\mathbf{a}_3)(\mathbf{a}_1^\top\mathbf{c}) - (\mathbf{a}_1^\top\mathbf{a}_1)(\mathbf{a}_1^\top\mathbf{b}_1)(\mathbf{a}_2^\top\mathbf{b}_2) + 12(\mathbf{a}_1^\top\mathbf{a}_1)(\mathbf{a}_1^\top\mathbf{b}_2)(\mathbf{a}_2^\top\mathbf{b}_1) - 8(\mathbf{a}_1^\top\mathbf{a}_1)(\mathbf{a}_1^\top\mathbf{a}_3)(\mathbf{a}_2^\top\mathbf{c})\\
&\quad +(\mathbf{a}_1^\top\mathbf{a}_1)(\mathbf{a}_2^\top\mathbf{a}_3)(\mathbf{a}_1^\top\mathbf{c}) - (\mathbf{a}_1^\top\mathbf{a}_1)(\mathbf{a}_1^\top\mathbf{b}_1)(\mathbf{a}_3^\top\mathbf{b}_1) + (\mathbf{a}_1^\top\mathbf{a}_1)(\mathbf{a}_1^\top\mathbf{a}_2)(\mathbf{a}_3^\top\mathbf{c}) + (\mathbf{a}_1^\top\mathbf{a}_1)(\mathbf{a}_1^\top\mathbf{b}_1)(\mathbf{a}_1^\top\mathbf{c}) - (\mathbf{a}_1^\top\mathbf{a}_1)(\mathbf{a}_1^\top\mathbf{a}_2)(\mathbf{b}_1^\top\mathbf{b}_2) + 4(\mathbf{a}_1^\top\mathbf{a}_1)(\mathbf{a}_1^\top\mathbf{a}_3)(\mathbf{b}_1^\top\mathbf{b}_1),\\
\lambda_7 &= 2(\mathbf{a}_2^\top\mathbf{a}_2)(\mathbf{a}_3^\top\mathbf{a}_3)^2 - 2(\mathbf{a}_2^\top\mathbf{a}_3)^2(\mathbf{a}_3^\top\mathbf{a}_3) + 8(\mathbf{a}_2^\top\mathbf{a}_2)(\mathbf{a}_1^\top\mathbf{b}_2)^2 - 4(\mathbf{a}_2^\top\mathbf{a}_3)^2(\mathbf{a}_1^\top\mathbf{b}_2) - 2(\mathbf{a}_3^\top\mathbf{a}_3)(\mathbf{a}_1^\top\mathbf{b}_1)^2 - 4(\mathbf{a}_1^\top\mathbf{b}_1)^2(\mathbf{a}_1^\top\mathbf{b}_2) - 8(\mathbf{a}_1^\top\mathbf{a}_3)^2(\mathbf{a}_2^\top\mathbf{c})\\
&\quad +4(\mathbf{a}_1^\top\mathbf{a}_3)^2(\mathbf{b}_1^\top\mathbf{b}_1) - 4(\mathbf{a}_1^\top\mathbf{a}_2)^2(\mathbf{b}_2^\top\mathbf{b}_2) - 4(\mathbf{a}_1^\top\mathbf{a}_3)(\mathbf{a}_2^\top\mathbf{a}_3)(\mathbf{a}_2^\top\mathbf{b}_2) - 12(\mathbf{a}_1^\top\mathbf{a}_2)(\mathbf{a}_2^\top\mathbf{a}_3)(\mathbf{a}_3^\top\mathbf{b}_2) - 4(\mathbf{a}_1^\top\mathbf{a}_2)(\mathbf{a}_3^\top\mathbf{a}_3)(\mathbf{a}_2^\top\mathbf{b}_2) + 16(\mathbf{a}_1^\top\mathbf{a}_3)(\mathbf{a}_2^\top\mathbf{a}_2)(\mathbf{a}_3^\top\mathbf{b}_2)\\
&\quad -4(\mathbf{a}_1^\top\mathbf{a}_3)(\mathbf{a}_2^\top\mathbf{a}_3)(\mathbf{a}_3^\top\mathbf{b}_1) + 12(\mathbf{a}_1^\top\mathbf{a}_3)(\mathbf{a}_3^\top\mathbf{a}_3)(\mathbf{a}_2^\top\mathbf{b}_1) + 8(\mathbf{a}_2^\top\mathbf{a}_2)(\mathbf{a}_3^\top\mathbf{a}_3)(\mathbf{a}_1^\top\mathbf{b}_2) - 4(\mathbf{a}_2^\top\mathbf{a}_3)(\mathbf{a}_3^\top\mathbf{a}_3)(\mathbf{a}_1^\top\mathbf{b}_1) - 4(\mathbf{a}_1^\top\mathbf{a}_2)(\mathbf{a}_3^\top\mathbf{a}_3)(\mathbf{a}_3^\top\mathbf{b}_1)\\
&\quad -8(\mathbf{a}_1^\top\mathbf{a}_2)(\mathbf{a}_1^\top\mathbf{b}_2)(\mathbf{a}_2^\top\mathbf{b}_2) - 4(\mathbf{a}_1^\top\mathbf{a}_3)(\mathbf{a}_1^\top\mathbf{b}_1)(\mathbf{a}_2^\top\mathbf{b}_2) + 24(\mathbf{a}_1^\top\mathbf{a}_3)(\mathbf{a}_1^\top\mathbf{b}_2)(\mathbf{a}_2^\top\mathbf{b}_1) - 8(\mathbf{a}_2^\top\mathbf{a}_3)(\mathbf{a}_1^\top\mathbf{b}_1)(\mathbf{a}_1^\top\mathbf{b}_2) + 4(\mathbf{a}_1^\top\mathbf{a}_3)(\mathbf{a}_2^\top\mathbf{a}_3)(\mathbf{a}_1^\top\mathbf{c})\\
&\quad -12(\mathbf{a}_1^\top\mathbf{a}_2)(\mathbf{a}_1^\top\mathbf{b}_1)(\mathbf{a}_3^\top\mathbf{b}_2) - 8(\mathbf{a}_1^\top\mathbf{a}_2)(\mathbf{a}_1^\top\mathbf{b}_2)(\mathbf{a}_3^\top\mathbf{b}_1) - 4(\mathbf{a}_1^\top\mathbf{a}_3)(\mathbf{a}_1^\top\mathbf{b}_1)(\mathbf{a}_3^\top\mathbf{b}_1) + 4(\mathbf{a}_1^\top\mathbf{a}_2)(\mathbf{a}_1^\top\mathbf{a}_3)(\mathbf{a}_3^\top\mathbf{c}) + 4(\mathbf{a}_1^\top\mathbf{a}_2)(\mathbf{a}_3^\top\mathbf{a}_3)(\mathbf{a}_1^\top\mathbf{c})\\
&\quad +12(\mathbf{a}_1^\top\mathbf{a}_1)(\mathbf{a}_2^\top\mathbf{b}_1)(\mathbf{a}_3^\top\mathbf{b}_2) - 4(\mathbf{a}_1^\top\mathbf{a}_1)(\mathbf{a}_3^\top\mathbf{a}_3)(\mathbf{a}_2^\top\mathbf{c}) + 8(\mathbf{a}_1^\top\mathbf{a}_2)(\mathbf{a}_1^\top\mathbf{b}_2)(\mathbf{a}_1^\top\mathbf{c}) + 4(\mathbf{a}_1^\top\mathbf{a}_3)(\mathbf{a}_1^\top\mathbf{b}_1)(\mathbf{a}_1^\top\mathbf{c}) - 8(\mathbf{a}_1^\top\mathbf{a}_1)(\mathbf{a}_1^\top\mathbf{b}_2)(\mathbf{a}_2^\top\mathbf{c})\\
&\quad -4(\mathbf{a}_1^\top\mathbf{a}_2)(\mathbf{a}_1^\top\mathbf{a}_3)(\mathbf{b}_1^\top\mathbf{b}_2) + 4(\mathbf{a}_1^\top\mathbf{a}_1)(\mathbf{a}_2^\top\mathbf{a}_2)(\mathbf{b}_2^\top\mathbf{b}_2) + 2(\mathbf{a}_1^\top\mathbf{a}_1)(\mathbf{a}_3^\top\mathbf{a}_3)(\mathbf{b}_1^\top\mathbf{b}_1) + 4(\mathbf{a}_1^\top\mathbf{a}_1)(\mathbf{a}_1^\top\mathbf{b}_2)(\mathbf{b}_1^\top\mathbf{b}_1) - 4(\mathbf{a}_1^\top\mathbf{a}_1)(\mathbf{a}_1^\top\mathbf{a}_3)(\mathbf{b}_1^\top\mathbf{c}),\\
\lambda_6 &= (\mathbf{a}_3^\top\mathbf{b}_2)[(\mathbf{a}_2^\top\mathbf{a}_3 + \mathbf{a}_1^\top\mathbf{b}_1)^2 + 2(\mathbf{a}_1^\top\mathbf{a}_2)(\mathbf{a}_2^\top\mathbf{b}_2 + \mathbf{a}_3^\top\mathbf{b}_1 - \mathbf{a}_1^\top\mathbf{c})]\\
&\quad -2(\mathbf{a}_2^\top\mathbf{b}_2 + \mathbf{a}_3^\top\mathbf{b}_1 - \mathbf{a}_1^\top\mathbf{c})[(\mathbf{a}_2^\top\mathbf{a}_3 + \mathbf{a}_1^\top\mathbf{b}_1)(\mathbf{a}_3^\top\mathbf{a}_3 + 2\mathbf{a}_1^\top\mathbf{b}_2) + 2(\mathbf{a}_1^\top\mathbf{a}_2)(\mathbf{a}_3^\top\mathbf{b}_2) - (\mathbf{a}_1^\top\mathbf{a}_1)(\mathbf{a}_3^\top\mathbf{c} - \mathbf{b}_1^\top\mathbf{b}_2) + 2(\mathbf{a}_1^\top\mathbf{a}_3)(\mathbf{a}_2^\top\mathbf{b}_2 + \mathbf{a}_3^\top\mathbf{b}_1 - \mathbf{a}_1^\top\mathbf{c})]\\
&\quad -4[(\mathbf{a}_1^\top\mathbf{a}_1)(\mathbf{a}_3^\top\mathbf{b}_2) + (\mathbf{a}_1^\top\mathbf{a}_3)(\mathbf{a}_3^\top\mathbf{a}_3 + 2\mathbf{a}_1^\top\mathbf{b}_2)](2\mathbf{a}_2^\top\mathbf{c} - \mathbf{b}_1^\top\mathbf{b}_1) + 3(\mathbf{a}_2^\top\mathbf{b}_1)[8(\mathbf{a}_1^\top\mathbf{a}_3)(\mathbf{a}_3^\top\mathbf{b}_2) + 2(\mathbf{a}_1^\top\mathbf{a}_1)(\mathbf{b}_2^\top\mathbf{b}_2) + (\mathbf{a}_3^\top\mathbf{a}_3 + 2\mathbf{a}_1^\top\mathbf{b}_2)^2]\\
&\quad -2[(\mathbf{a}_1^\top\mathbf{a}_2)(\mathbf{a}_3^\top\mathbf{c} - \mathbf{b}_1^\top\mathbf{b}_2) - (\mathbf{a}_2^\top\mathbf{a}_3 + \mathbf{a}_1^\top\mathbf{b}_1)(\mathbf{a}_2^\top\mathbf{b}_2 + \mathbf{a}_3^\top\mathbf{b}_1 - \mathbf{a}_1^\top\mathbf{c})](\mathbf{a}_3^\top\mathbf{a}_3 + 2\mathbf{a}_1^\top\mathbf{b}_2) - 2(\mathbf{b}_1^\top\mathbf{c})((\mathbf{a}_1^\top\mathbf{a}_1)((\mathbf{a}_3^\top\mathbf{a}_3) + 2(\mathbf{a}_1^\top\mathbf{b}_2)) + 2(\mathbf{a}_1^\top\mathbf{a}_3)^2)\\
&\quad -3(\mathbf{a}_1^\top\mathbf{a}_3)[2(\mathbf{a}_1^\top\mathbf{a}_2)(\mathbf{b}_2^\top\mathbf{c}) - (\mathbf{a}_2^\top\mathbf{b}_2 + \mathbf{a}_3^\top\mathbf{b}_1 - \mathbf{a}_1^\top\mathbf{c})^2 + 2(\mathbf{a}_3^\top\mathbf{c} - \mathbf{b}_1^\top\mathbf{b}_2)(\mathbf{a}_2^\top\mathbf{a}_3 + \mathbf{a}_1^\top\mathbf{b}_1)]\\
&\quad +(\mathbf{a}_3^\top\mathbf{c} - \mathbf{b}_1^\top\mathbf{b}_2)[(\mathbf{a}_1^\top\mathbf{a}_2)(\mathbf{a}_3^\top\mathbf{a}_3 + 2\mathbf{a}_1^\top\mathbf{b}_2) + 2(\mathbf{a}_1^\top\mathbf{a}_3)(\mathbf{a}_2^\top\mathbf{a}_3 + \mathbf{a}_1^\top\mathbf{b}_1) + (\mathbf{a}_1^\top\mathbf{a}_1)(\mathbf{a}_2^\top\mathbf{b}_2 + \mathbf{a}_3^\top\mathbf{b}_1 - \mathbf{a}_1^\top\mathbf{c})]\\
&\quad -4(\mathbf{a}_1^\top\mathbf{a}_1)[(\mathbf{a}_3^\top\mathbf{c} - \mathbf{b}_1^\top\mathbf{b}_2)(\mathbf{a}_2^\top\mathbf{b}_2 + \mathbf{a}_3^\top\mathbf{b}_1 - \mathbf{a}_1^\top\mathbf{c}) + (\mathbf{b}_2^\top\mathbf{c})(\mathbf{a}_2^\top\mathbf{a}_3 + \mathbf{a}_1^\top\mathbf{b}_1)] + 8(\mathbf{a}_2^\top\mathbf{a}_2)[(\mathbf{a}_1^\top\mathbf{a}_3)(\mathbf{b}_2^\top\mathbf{b}_2) + (\mathbf{a}_3^\top\mathbf{b}_2)(\mathbf{a}_3^\top\mathbf{a}_3 + 2\mathbf{a}_1^\top\mathbf{b}_2)]\\
&\quad +4(\mathbf{a}_1^\top\mathbf{a}_2)[2(\mathbf{a}_1^\top\mathbf{a}_3)(\mathbf{b}_2^\top\mathbf{c}) - 2(\mathbf{a}_3^\top\mathbf{b}_2)(\mathbf{a}_2^\top\mathbf{b}_2 + \mathbf{a}_3^\top\mathbf{b}_1 - \mathbf{a}_1^\top\mathbf{c}) + (\mathbf{a}_3^\top\mathbf{a}_3 + 2\mathbf{a}_1^\top\mathbf{b}_2)(\mathbf{a}_3^\top\mathbf{c} - \mathbf{b}_1^\top\mathbf{b}_2) - (\mathbf{b}_2^\top\mathbf{b}_2)(\mathbf{a}_2^\top\mathbf{a}_3 + \mathbf{a}_1^\top\mathbf{b}_1)]\\
&\quad -3(\mathbf{a}_2^\top\mathbf{a}_3 + \mathbf{a}_1^\top\mathbf{b}_1)[(\mathbf{a}_1^\top\mathbf{a}_2)(\mathbf{b}_2^\top\mathbf{b}_2) - (\mathbf{a}_1^\top\mathbf{a}_1)(\mathbf{b}_2^\top\mathbf{c}) + 2(\mathbf{a}_3^\top\mathbf{b}_2)(\mathbf{a}_2^\top\mathbf{a}_3 + \mathbf{a}_1^\top\mathbf{b}_1) - 2(\mathbf{a}_1^\top\mathbf{a}_3)(\mathbf{a}_3^\top\mathbf{c} - \mathbf{b}_1^\top\mathbf{b}_2) + (\mathbf{a}_3^\top\mathbf{a}_3 + 2\mathbf{a}_1^\top\mathbf{b}_2)(\mathbf{a}_2^\top\mathbf{b}_2 + \mathbf{a}_3^\top\mathbf{b}_1 - \mathbf{a}_1^\top\mathbf{c})],\\
\lambda_5 &= (\mathbf{a}_3^\top\mathbf{c} - \mathbf{b}_1^\top\mathbf{b}_2)[(\mathbf{a}_2^\top\mathbf{a}_3 + \mathbf{a}_1^\top\mathbf{b}_1)(\mathbf{a}_3^\top\mathbf{a}_3 + 2\mathbf{a}_1^\top\mathbf{b}_2) + 2(\mathbf{a}_1^\top\mathbf{a}_2)(\mathbf{a}_3^\top\mathbf{b}_2) - (\mathbf{a}_1^\top\mathbf{a}_1)(\mathbf{a}_3^\top\mathbf{c} - \mathbf{b}_1^\top\mathbf{b}_2) + 2(\mathbf{a}_1^\top\mathbf{a}_3)(\mathbf{a}_2^\top\mathbf{b}_2 + \mathbf{a}_3^\top\mathbf{b}_1 - \mathbf{a}_1^\top\mathbf{c})]\\
&\quad +3(\mathbf{a}_2^\top\mathbf{a}_3 + \mathbf{a}_1^\top\mathbf{b}_1)[2(\mathbf{a}_1^\top\mathbf{a}_3)(\mathbf{b}_2^\top\mathbf{c}) - 2(\mathbf{a}_3^\top\mathbf{b}_2)(\mathbf{a}_2^\top\mathbf{b}_2 + \mathbf{a}_3^\top\mathbf{b}_1 - \mathbf{a}_1^\top\mathbf{c}) + (\mathbf{a}_3^\top\mathbf{a}_3 + 2\mathbf{a}_1^\top\mathbf{b}_2)(\mathbf{a}_3^\top\mathbf{c} - \mathbf{b}_1^\top\mathbf{b}_2) - (\mathbf{b}_2^\top\mathbf{b}_2)(\mathbf{a}_2^\top\mathbf{a}_3 + \mathbf{a}_1^\top\mathbf{b}_1)]\\
&\quad +4(\mathbf{a}_2^\top\mathbf{a}_2)[(\mathbf{b}_2^\top\mathbf{b}_2)(\mathbf{a}_3^\top\mathbf{a}_3 + 2\mathbf{a}_1^\top\mathbf{b}_2) + 2(\mathbf{a}_3^\top\mathbf{b}_2)^2] - 4(\mathbf{b}_1^\top\mathbf{c})[(\mathbf{a}_1^\top\mathbf{a}_1)(\mathbf{a}_3^\top\mathbf{b}_2) + (\mathbf{a}_1^\top\mathbf{a}_3)(\mathbf{a}_3^\top\mathbf{a}_3 + 2\mathbf{a}_1^\top\mathbf{b}_2)]\\
&\quad +4(\mathbf{a}_1^\top\mathbf{a}_2)[(\mathbf{b}_2^\top\mathbf{c})(\mathbf{a}_3^\top\mathbf{a}_3 + 2\mathbf{a}_1^\top\mathbf{b}_2) + 2(\mathbf{a}_3^\top\mathbf{b}_2)(\mathbf{a}_3^\top\mathbf{c} - \mathbf{b}_1^\top\mathbf{b}_2) - (\mathbf{b}_2^\top\mathbf{b}_2)(\mathbf{a}_2^\top\mathbf{b}_2 + \mathbf{a}_3^\top\mathbf{b}_1 - \mathbf{a}_1^\top\mathbf{c})]\\
&\quad -2(\mathbf{a}_2^\top\mathbf{b}_2 + \mathbf{a}_3^\top\mathbf{b}_1 - \mathbf{a}_1^\top\mathbf{c})[(\mathbf{a}_1^\top\mathbf{a}_2)(\mathbf{b}_2^\top\mathbf{b}_2) - (\mathbf{a}_1^\top\mathbf{a}_1)(\mathbf{b}_2^\top\mathbf{c}) + 2(\mathbf{a}_3^\top\mathbf{b}_2)(\mathbf{a}_2^\top\mathbf{a}_3 + \mathbf{a}_1^\top\mathbf{b}_1) - 2(\mathbf{a}_1^\top\mathbf{a}_3)(\mathbf{a}_3^\top\mathbf{c} - \mathbf{b}_1^\top\mathbf{b}_2) + (\mathbf{a}_3^\top\mathbf{a}_3 + 2\mathbf{a}_1^\top\mathbf{b}_2)(\mathbf{a}_2^\top\mathbf{b}_2 + \mathbf{a}_3^\top\mathbf{b}_1 - \mathbf{a}_1^\top\mathbf{c})]\\
&\quad -2(\mathbf{a}_1^\top\mathbf{a}_1)[2(\mathbf{b}_2^\top\mathbf{c})(\mathbf{a}_2^\top\mathbf{b}_2 + \mathbf{a}_3^\top\mathbf{b}_1 - \mathbf{a}_1^\top\mathbf{c}) - (\mathbf{a}_3^\top\mathbf{c} - \mathbf{b}_1^\top\mathbf{b}_2)^2] - (2\mathbf{a}_2^\top\mathbf{c} - \mathbf{b}_1^\top\mathbf{b}_1)[8(\mathbf{a}_1^\top\mathbf{a}_3)(\mathbf{a}_3^\top\mathbf{b}_2) + 2(\mathbf{a}_1^\top\mathbf{a}_1)(\mathbf{b}_2^\top\mathbf{b}_2) + (\mathbf{a}_3^\top\mathbf{a}_3 + 2\mathbf{a}_1^\top\mathbf{b}_2)^2]\\
&\quad -(\mathbf{a}_3^\top\mathbf{a}_3 + 2\mathbf{a}_1^\top\mathbf{b}_2)[2(\mathbf{a}_1^\top\mathbf{a}_2)(\mathbf{b}_2^\top\mathbf{c}) - (\mathbf{a}_2^\top\mathbf{b}_2 + \mathbf{a}_3^\top\mathbf{b}_1 - \mathbf{a}_1^\top\mathbf{c})^2 + 2(\mathbf{a}_3^\top\mathbf{c} - \mathbf{b}_1^\top\mathbf{b}_2)(\mathbf{a}_2^\top\mathbf{a}_3 + \mathbf{a}_1^\top\mathbf{b}_1)]\\
&\quad -2(\mathbf{a}_3^\top\mathbf{b}_2)[(\mathbf{a}_1^\top\mathbf{a}_2)(\mathbf{a}_3^\top\mathbf{c} - \mathbf{b}_1^\top\mathbf{b}_2) - (\mathbf{a}_2^\top\mathbf{a}_3 + \mathbf{a}_1^\top\mathbf{b}_1)(\mathbf{a}_2^\top\mathbf{b}_2 + \mathbf{a}_3^\top\mathbf{b}_1 - \mathbf{a}_1^\top\mathbf{c})]\\
&\quad -6(\mathbf{a}_1^\top\mathbf{a}_3)[(\mathbf{a}_3^\top\mathbf{c} - \mathbf{b}_1^\top\mathbf{b}_2)(\mathbf{a}_2^\top\mathbf{b}_2 + \mathbf{a}_3^\top\mathbf{b}_1 - \mathbf{a}_1^\top\mathbf{c}) + (\mathbf{b}_2^\top\mathbf{c})(\mathbf{a}_2^\top\mathbf{a}_3 + \mathbf{a}_1^\top\mathbf{b}_1)] + 12(\mathbf{a}_2^\top\mathbf{b}_1)[(\mathbf{a}_1^\top\mathbf{a}_3)(\mathbf{b}_2^\top\mathbf{b}_2) + (\mathbf{a}_3^\top\mathbf{b}_2)(\mathbf{a}_3^\top\mathbf{a}_3 + 2\mathbf{a}_1^\top\mathbf{b}_2)],\\
\lambda_4 &= 3(\mathbf{a}_2^\top\mathbf{a}_3 + \mathbf{a}_1^\top\mathbf{b}_1)[(\mathbf{b}_2^\top\mathbf{c})(\mathbf{a}_3^\top\mathbf{a}_3 + 2\mathbf{a}_1^\top\mathbf{b}_2) + 2(\mathbf{a}_3^\top\mathbf{b}_2)(\mathbf{a}_3^\top\mathbf{c} - \mathbf{b}_1^\top\mathbf{b}_2) - (\mathbf{b}_2^\top\mathbf{b}_2)(\mathbf{a}_2^\top\mathbf{b}_2 + \mathbf{a}_3^\top\mathbf{b}_1 - \mathbf{a}_1^\top\mathbf{c})]\\
&\quad +2(\mathbf{a}_2^\top\mathbf{b}_2 + \mathbf{a}_3^\top\mathbf{b}_1 - \mathbf{a}_1^\top\mathbf{c})[2(\mathbf{a}_1^\top\mathbf{a}_3)(\mathbf{b}_2^\top\mathbf{c}) - 2(\mathbf{a}_3^\top\mathbf{b}_2)(\mathbf{a}_2^\top\mathbf{b}_2 + \mathbf{a}_3^\top\mathbf{b}_1 - \mathbf{a}_1^\top\mathbf{c}) + (\mathbf{a}_3^\top\mathbf{a}_3 + 2\mathbf{a}_1^\top\mathbf{b}_2)(\mathbf{a}_3^\top\mathbf{c} - \mathbf{b}_1^\top\mathbf{b}_2) - (\mathbf{b}_2^\top\mathbf{b}_2)(\mathbf{a}_2^\top\mathbf{a}_3 + \mathbf{a}_1^\top\mathbf{b}_1)]\\
&\quad -(\mathbf{b}_1^\top\mathbf{c})[8(\mathbf{a}_1^\top\mathbf{a}_3)(\mathbf{a}_3^\top\mathbf{b}_2) + 2(\mathbf{a}_1^\top\mathbf{a}_1)(\mathbf{b}_2^\top\mathbf{b}_2) + (\mathbf{a}_3^\top\mathbf{a}_3 + 2\mathbf{a}_1^\top\mathbf{b}_2)^2]\\
&\quad -2(\mathbf{a}_3^\top\mathbf{a}_3 + 2\mathbf{a}_1^\top\mathbf{b}_2)[(\mathbf{a}_3^\top\mathbf{c} - \mathbf{b}_1^\top\mathbf{b}_2)(\mathbf{a}_2^\top\mathbf{b}_2 + \mathbf{a}_3^\top\mathbf{b}_1 - \mathbf{a}_1^\top\mathbf{c}) + (\mathbf{b}_2^\top\mathbf{c})(\mathbf{a}_2^\top\mathbf{a}_3 + \mathbf{a}_1^\top\mathbf{b}_1)] - 4(2\mathbf{a}_2^\top\mathbf{c} - \mathbf{b}_1^\top\mathbf{b}_1)[(\mathbf{a}_1^\top\mathbf{a}_3)(\mathbf{b}_2^\top\mathbf{b}_2) + (\mathbf{a}_3^\top\mathbf{b}_2)(\mathbf{a}_3^\top\mathbf{a}_3 + 2\mathbf{a}_1^\top\mathbf{b}_2)]\\
&\quad +6(\mathbf{a}_2^\top\mathbf{b}_1)[(\mathbf{b}_2^\top\mathbf{b}_2)(\mathbf{a}_3^\top\mathbf{a}_3 + 2\mathbf{a}_1^\top\mathbf{b}_2) + 2(\mathbf{a}_3^\top\mathbf{b}_2)^2] + 4(\mathbf{a}_1^\top\mathbf{a}_2)[2(\mathbf{a}_3^\top\mathbf{b}_2)(\mathbf{b}_2^\top\mathbf{c}) + (\mathbf{b}_2^\top\mathbf{b}_2)(\mathbf{a}_3^\top\mathbf{c} - \mathbf{b}_1^\top\mathbf{b}_2)]\\
&\quad +(\mathbf{a}_3^\top\mathbf{c} - \mathbf{b}_1^\top\mathbf{b}_2)[(\mathbf{a}_1^\top\mathbf{a}_2)(\mathbf{b}_2^\top\mathbf{b}_2) - (\mathbf{a}_1^\top\mathbf{a}_1)(\mathbf{b}_2^\top\mathbf{c}) + 2(\mathbf{a}_3^\top\mathbf{b}_2)(\mathbf{a}_2^\top\mathbf{a}_3 + \mathbf{a}_1^\top\mathbf{b}_1) - 2(\mathbf{a}_1^\top\mathbf{a}_3)(\mathbf{a}_3^\top\mathbf{c} - \mathbf{b}_1^\top\mathbf{b}_2) + (\mathbf{a}_3^\top\mathbf{a}_3 + 2\mathbf{a}_1^\top\mathbf{b}_2)(\mathbf{a}_2^\top\mathbf{b}_2 + \mathbf{a}_3^\top\mathbf{b}_1 - \mathbf{a}_1^\top\mathbf{c})]\\
&\quad -(\mathbf{a}_3^\top\mathbf{b}_2)[2(\mathbf{a}_1^\top\mathbf{a}_2)(\mathbf{b}_2^\top\mathbf{c}) - (\mathbf{a}_2^\top\mathbf{b}_2 + \mathbf{a}_3^\top\mathbf{b}_1 - \mathbf{a}_1^\top\mathbf{c})^2 + 2(\mathbf{a}_3^\top\mathbf{c} - \mathbf{b}_1^\top\mathbf{b}_2)(\mathbf{a}_2^\top\mathbf{a}_3 + \mathbf{a}_1^\top\mathbf{b}_1)]\\
&\quad -3(\mathbf{a}_1^\top\mathbf{a}_3)[2(\mathbf{b}_2^\top\mathbf{c})(\mathbf{a}_2^\top\mathbf{b}_2 + \mathbf{a}_3^\top\mathbf{b}_1 - \mathbf{a}_1^\top\mathbf{c}) - (\mathbf{a}_3^\top\mathbf{c} - \mathbf{b}_1^\top\mathbf{b}_2)^2] + 8(\mathbf{a}_2^\top\mathbf{a}_2)(\mathbf{a}_3^\top\mathbf{b}_2)(\mathbf{b}_2^\top\mathbf{b}_2) + 4(\mathbf{a}_1^\top\mathbf{a}_1)(\mathbf{b}_2^\top\mathbf{c})(\mathbf{a}_3^\top\mathbf{c} - \mathbf{b}_1^\top\mathbf{b}_2),\\
\lambda_3 &= 3(\mathbf{a}_2^\top\mathbf{a}_3 + \mathbf{a}_1^\top\mathbf{b}_1)[2(\mathbf{a}_3^\top\mathbf{b}_2)(\mathbf{b}_2^\top\mathbf{c}) + (\mathbf{b}_2^\top\mathbf{b}_2)(\mathbf{a}_3^\top\mathbf{c} - \mathbf{b}_1^\top\mathbf{b}_2)] - 2(2\mathbf{a}_2^\top\mathbf{c} - \mathbf{b}_1^\top\mathbf{b}_1)[(\mathbf{b}_2^\top\mathbf{b}_2)(\mathbf{a}_3^\top\mathbf{a}_3 + 2\mathbf{a}_1^\top\mathbf{b}_2) + 2(\mathbf{a}_3^\top\mathbf{b}_2)^2]\\
&\quad -(\mathbf{a}_3^\top\mathbf{c} - \mathbf{b}_1^\top\mathbf{b}_2)[2(\mathbf{a}_1^\top\mathbf{a}_3)(\mathbf{b}_2^\top\mathbf{c}) - 2(\mathbf{a}_3^\top\mathbf{b}_2)(\mathbf{a}_2^\top\mathbf{b}_2 + \mathbf{a}_3^\top\mathbf{b}_1 - \mathbf{a}_1^\top\mathbf{c}) + (\mathbf{a}_3^\top\mathbf{a}_3 + 2\mathbf{a}_1^\top\mathbf{b}_2)(\mathbf{a}_3^\top\mathbf{c} - \mathbf{b}_1^\top\mathbf{b}_2) - (\mathbf{b}_2^\top\mathbf{b}_2)(\mathbf{a}_2^\top\mathbf{a}_3 + \mathbf{a}_1^\top\mathbf{b}_1)]\\
&\quad +2(\mathbf{a}_2^\top\mathbf{a}_2)(\mathbf{b}_2^\top\mathbf{b}_2)^2 + 2(\mathbf{a}_1^\top\mathbf{a}_1)(\mathbf{b}_2^\top\mathbf{c})^2 - 2(\mathbf{a}_3^\top\mathbf{b}_2)[(\mathbf{a}_3^\top\mathbf{c} - \mathbf{b}_1^\top\mathbf{b}_2)(\mathbf{a}_2^\top\mathbf{b}_2 + \mathbf{a}_3^\top\mathbf{b}_1 - \mathbf{a}_1^\top\mathbf{c}) + (\mathbf{b}_2^\top\mathbf{c})(\mathbf{a}_2^\top\mathbf{a}_3 + \mathbf{a}_1^\top\mathbf{b}_1)]\\
&\quad -(\mathbf{a}_3^\top\mathbf{a}_3 + 2\mathbf{a}_1^\top\mathbf{b}_2)[2(\mathbf{b}_2^\top\mathbf{c})(\mathbf{a}_2^\top\mathbf{b}_2 + \mathbf{a}_3^\top\mathbf{b}_1 - \mathbf{a}_1^\top\mathbf{c}) - (\mathbf{a}_3^\top\mathbf{c} - \mathbf{b}_1^\top\mathbf{b}_2)^2] - 4(\mathbf{b}_1^\top\mathbf{c})[(\mathbf{a}_1^\top\mathbf{a}_3)(\mathbf{b}_2^\top\mathbf{b}_2) + (\mathbf{a}_3^\top\mathbf{b}_2)(\mathbf{a}_3^\top\mathbf{a}_3 + 2\mathbf{a}_1^\top\mathbf{b}_2)]\\
&\quad +2(\mathbf{a}_2^\top\mathbf{b}_2 + \mathbf{a}_3^\top\mathbf{b}_1 - \mathbf{a}_1^\top\mathbf{c})[(\mathbf{b}_2^\top\mathbf{c})(\mathbf{a}_3^\top\mathbf{a}_3 + 2\mathbf{a}_1^\top\mathbf{b}_2) + 2(\mathbf{a}_3^\top\mathbf{b}_2)(\mathbf{a}_3^\top\mathbf{c} - \mathbf{b}_1^\top\mathbf{b}_2) - (\mathbf{b}_2^\top\mathbf{b}_2)(\mathbf{a}_2^\top\mathbf{b}_2 + \mathbf{a}_3^\top\mathbf{b}_1 - \mathbf{a}_1^\top\mathbf{c})]\\
&\quad +12(\mathbf{a}_2^\top\mathbf{b}_1)(\mathbf{a}_3^\top\mathbf{b}_2)(\mathbf{b}_2^\top\mathbf{b}_2) + 4(\mathbf{a}_1^\top\mathbf{a}_2)(\mathbf{b}_2^\top\mathbf{b}_2)(\mathbf{b}_2^\top\mathbf{c}) + 6(\mathbf{a}_1^\top\mathbf{a}_3)(\mathbf{b}_2^\top\mathbf{c})(\mathbf{a}_3^\top\mathbf{c} - \mathbf{b}_1^\top\mathbf{b}_2),\\
\lambda_2 &= 3(\mathbf{a}_1^\top\mathbf{a}_3)(\mathbf{b}_2^\top\mathbf{c})^2 + 3(\mathbf{a}_2^\top\mathbf{b}_1)(\mathbf{b}_2^\top\mathbf{b}_2)^2 - 2(\mathbf{b}_1^\top\mathbf{c})[(\mathbf{b}_2^\top\mathbf{b}_2)(\mathbf{a}_3^\top\mathbf{a}_3 + 2\mathbf{a}_1^\top\mathbf{b}_2) + 2(\mathbf{a}_3^\top\mathbf{b}_2)^2]\\
&\quad -(\mathbf{a}_3^\top\mathbf{b}_2)[2(\mathbf{b}_2^\top\mathbf{c})(\mathbf{a}_2^\top\mathbf{b}_2 + \mathbf{a}_3^\top\mathbf{b}_1 - \mathbf{a}_1^\top\mathbf{c}) - (\mathbf{a}_3^\top\mathbf{c} - \mathbf{b}_1^\top\mathbf{b}_2)^2] + 2(\mathbf{a}_2^\top\mathbf{b}_2 + \mathbf{a}_3^\top\mathbf{b}_1 - \mathbf{a}_1^\top\mathbf{c})[2(\mathbf{a}_3^\top\mathbf{b}_2)(\mathbf{b}_2^\top\mathbf{c}) + (\mathbf{b}_2^\top\mathbf{b}_2)(\mathbf{a}_3^\top\mathbf{c} - \mathbf{b}_1^\top\mathbf{b}_2)]\\
&\quad -(\mathbf{a}_3^\top\mathbf{c} - \mathbf{b}_1^\top\mathbf{b}_2)[(\mathbf{b}_2^\top\mathbf{c})(\mathbf{a}_3^\top\mathbf{a}_3 + 2\mathbf{a}_1^\top\mathbf{b}_2) + 2(\mathbf{a}_3^\top\mathbf{b}_2)(\mathbf{a}_3^\top\mathbf{c} - \mathbf{b}_1^\top\mathbf{b}_2) - (\mathbf{b}_2^\top\mathbf{b}_2)(\mathbf{a}_2^\top\mathbf{b}_2 + \mathbf{a}_3^\top\mathbf{b}_1 - \mathbf{a}_1^\top\mathbf{c})]\\
&\quad +3(\mathbf{b}_2^\top\mathbf{b}_2)(\mathbf{b}_2^\top\mathbf{c})(\mathbf{a}_2^\top\mathbf{a}_3 + \mathbf{a}_1^\top\mathbf{b}_1) - 4(\mathbf{a}_3^\top\mathbf{b}_2)(\mathbf{b}_2^\top\mathbf{b}_2)(2\mathbf{a}_2^\top\mathbf{c} - \mathbf{b}_1^\top\mathbf{b}_1) + 2(\mathbf{b}_2^\top\mathbf{c})(\mathbf{a}_3^\top\mathbf{c} - \mathbf{b}_1^\top\mathbf{b}_2)(\mathbf{a}_3^\top\mathbf{a}_3 + 2\mathbf{a}_1^\top\mathbf{b}_2),\\
\lambda_1 &= (\mathbf{b}_2^\top\mathbf{c})^2((\mathbf{a}_3^\top\mathbf{a}_3) + 2(\mathbf{a}_1^\top\mathbf{b}_2)) - (\mathbf{b}_2^\top\mathbf{b}_2)^2(2(\mathbf{a}_2^\top\mathbf{c}) - (\mathbf{b}_1^\top\mathbf{b}_1)) - ((\mathbf{a}_3^\top\mathbf{c}) - (\mathbf{b}_1^\top\mathbf{b}_2))(2(\mathbf{a}_3^\top\mathbf{b}_2)(\mathbf{b}_2^\top\mathbf{c}) + (\mathbf{b}_2^\top\mathbf{b}_2)((\mathbf{a}_3^\top\mathbf{c}) - (\mathbf{b}_1^\top\mathbf{b}_2)))\\
&\quad -4(\mathbf{a}_3^\top\mathbf{b}_2)(\mathbf{b}_2^\top\mathbf{b}_2)(\mathbf{b}_1^\top\mathbf{c}) + 2(\mathbf{a}_3^\top\mathbf{b}_2)(\mathbf{b}_2^\top\mathbf{c})(\mathbf{a}_3^\top\mathbf{c} - \mathbf{b}_1^\top\mathbf{b}_2) + 2(\mathbf{b}_2^\top\mathbf{b}_2)(\mathbf{b}_2^\top\mathbf{c})(\mathbf{a}_2^\top\mathbf{b}_2 + \mathbf{a}_3^\top\mathbf{b}_1 - \mathbf{a}_1^\top\mathbf{c}),\\
\lambda_0 &= (\mathbf{a}_3^\top\mathbf{b}_2)(\mathbf{b}_2^\top\mathbf{c})^2 - (\mathbf{b}_2^\top\mathbf{b}_2)^2(\mathbf{b}_1^\top\mathbf{c}) - (\mathbf{b}_2^\top\mathbf{b}_2)(\mathbf{b}_2^\top\mathbf{c})(\mathbf{a}_3^\top\mathbf{c} - \mathbf{b}_1^\top\mathbf{b}_2),
\end{aligned}
\tag{8}
$$

*with* $\mathbf{a}_1 = [a_{11},\ a_{21}, \ldots,\ a_{D1}]^\top$, $\mathbf{a}_2 = [a_{12},\ a_{22}, \ldots,\ a_{D2}]^\top$, $\mathbf{a}_3 = [a_{13},\ a_{23}, \ldots,\ a_{D3}]^\top$, $\mathbf{b}_1 = [b_{11},\ b_{21}, \ldots,\ b_{D1}]^\top$, $\mathbf{b}_2 = [b_{12},\ b_{22}, \ldots,\ b_{D2}]^\top$, $\mathbf{c} = [c_1,\ c_2, \ldots,\ c_D]^\top$ *given* $a_{d1}$, $a_{d2}$, $a_{d3}$, $b_{d1}$, $b_{d2}$, $c_d$ *in (4) for* $d = 1, \ldots, D$, *and where* $^\top$ *denotes the transpose operator. The anisotropy ratio* $\alpha^2$ *can be calculated as follows*

$$
\alpha^2 = -\frac{\mathbf{a}_1^\top\mathbf{a}_2\kappa_\alpha^4 + (\mathbf{a}_2^\top\mathbf{a}_3 + \mathbf{a}_1^\top\mathbf{b}_1)\kappa_\alpha^3 + (\mathbf{a}_2^\top\mathbf{b}_2 + \mathbf{a}_3^\top\mathbf{b}_1 - \mathbf{a}_1^\top\mathbf{c})\kappa_\alpha^2 + (\mathbf{b}_2^\top\mathbf{b}_1 - \mathbf{a}_3^\top\mathbf{c})\kappa_\alpha - \mathbf{b}_2^\top\mathbf{c}}{\mathbf{a}_1^\top\mathbf{a}_1\kappa_\alpha^4 + 2\mathbf{a}_1^\top\mathbf{a}_3\kappa_\alpha^3 + (\mathbf{a}_3^\top\mathbf{a}_3 + 2\mathbf{a}_1^\top\mathbf{b}_2)\kappa_\alpha^2 + 2\mathbf{a}_3^\top\mathbf{b}_2\kappa_\alpha + \mathbf{b}_2^\top\mathbf{b}_2}. \tag{9}
$$

*Proof.* The following system of equations can be driven from (1)

$$\mathbf{A}\mathbf{y} + \mathbf{B}\mathbf{x} = \mathbf{c},$$

where $\mathbf{A} := [\mathbf{a}_1^\top\ \mathbf{a}_2^\top\ \mathbf{a}_3^\top]$, $\mathbf{B} := [\mathbf{b}_1^\top\ \mathbf{b}_2^\top]$, $\mathbf{x} := [x\ y]^\top$, $\mathbf{y} := [x^2y\ x^2\ xy]^\top$. The least square solution is therefore

$$\min\ \|\mathbf{A}\mathbf{y} + \mathbf{B}\mathbf{x} - \mathbf{c}\|^2,$$

where $\|\bullet\|$ is the L2-norm.

The procedure for finding the minimum consists of calculating the derivative with respect $x$ and $y$ and equating to zero, namely

$$\begin{aligned}
\frac{\partial}{\partial x}\|\mathbf{Ay}+\mathbf{Bx}-\mathbf{c}\|^2 &= 4\mathbf{a}_1^\top\mathbf{a}_1x^3y^2+8\mathbf{a}_1^\top\mathbf{a}_2x^3y+4\mathbf{a}_2^\top\mathbf{a}_2x^3+6\mathbf{a}_1^\top\mathbf{a}_3x^2y^2\\
&+6(\mathbf{a}_2^\top\mathbf{a}_3+\mathbf{a}_1^\top\mathbf{b}_1)x^2y+2(\mathbf{a}_3^\top\mathbf{a}_3+2\mathbf{a}_1^\top\mathbf{b}_2)xy^2+6\mathbf{a}_2^\top\mathbf{b}_1x^2\\
&+2\mathbf{a}_3^\top\mathbf{b}_2y^2+4(\mathbf{a}_2^\top\mathbf{b}_2+\mathbf{a}_3^\top\mathbf{b}_1-\mathbf{a}_1^\top\mathbf{c})xy\\
&+2(\mathbf{b}_1^\top\mathbf{b}_1-2\mathbf{a}_2^\top\mathbf{c})x+2(\mathbf{b}_1^\top\mathbf{b}_2-\mathbf{a}_3^\top\mathbf{c})y-2\mathbf{b}_1^\top\mathbf{c}\\
\frac{\partial}{\partial y}\|\mathbf{Ay}+\mathbf{Bx}-\mathbf{c}\|^2 &= 2\mathbf{a}_1^\top\mathbf{a}_1x^4y+(\mathbf{a}_1^\top\mathbf{a}_2+\mathbf{a}_2^\top\mathbf{a}_1)x^4+2(\mathbf{a}_1^\top\mathbf{a}_3+\mathbf{a}_3^\top\mathbf{a}_1)x^3y\\
&+(\mathbf{a}_2^\top\mathbf{a}_3+\mathbf{a}_3^\top\mathbf{a}_2+2\mathbf{a}_1^\top\mathbf{b}_1)x^3+2(\mathbf{a}_3^\top\mathbf{a}_3+2\mathbf{a}_1^\top\mathbf{b}_2)x^2y\\
&+2(\mathbf{a}_2^\top\mathbf{b}_2+\mathbf{a}_3^\top\mathbf{b}_1-\mathbf{a}_1^\top\mathbf{c})x^2+4\mathbf{a}_3^\top\mathbf{b}_2xy\\
&+2(\mathbf{b}_2^\top\mathbf{b}_1-\mathbf{a}_3^\top\mathbf{c})x+2\mathbf{b}_2^\top\mathbf{b}_2y-2\mathbf{b}_2^\top\mathbf{c}.
\end{aligned}$$

The derivatives above are zero when

$$\left\{\begin{aligned}
\mathbf{b}_1^\top\mathbf{c} &= 2\mathbf{a}_1^\top\mathbf{a}_1x^3y^2+4(\mathbf{a}_1^\top\mathbf{a}_2)x^3y+2\mathbf{a}_2^\top\mathbf{a}_2x^3+3(\mathbf{a}_1^\top\mathbf{a}_3)x^2y^2\\
&+3(\mathbf{a}_2^\top\mathbf{a}_3+\mathbf{a}_1^\top\mathbf{b}_1)x^2y+(\mathbf{a}_3^\top\mathbf{a}_3+2\mathbf{a}_1^\top\mathbf{b}_2)xy^2+3\mathbf{a}_2^\top\mathbf{b}_1x^2+\mathbf{a}_3^\top\mathbf{b}_2y^2\\
&+2(\mathbf{a}_2^\top\mathbf{b}_2+\mathbf{a}_3^\top\mathbf{b}_1-\mathbf{a}_1^\top\mathbf{c})xy+(\mathbf{b}_1^\top\mathbf{b}_1-2\mathbf{a}_2^\top\mathbf{c})x+(\mathbf{b}_1^\top\mathbf{b}_2-\mathbf{a}_3^\top\mathbf{c})y\\
\mathbf{b}_2^\top\mathbf{c} &= \mathbf{a}_1^\top\mathbf{a}_1x^4y+(\mathbf{a}_1^\top\mathbf{a}_2)x^4+2(\mathbf{a}_1^\top\mathbf{a}_3)x^3y+(\mathbf{a}_2^\top\mathbf{a}_3+\mathbf{a}_1^\top\mathbf{b}_1)x^3\\
&+(\mathbf{a}_3^\top\mathbf{a}_3+2\mathbf{a}_1^\top\mathbf{b}_2)x^2y+(\mathbf{a}_2^\top\mathbf{b}_2+\mathbf{a}_3^\top\mathbf{b}_1-\mathbf{a}_1^\top\mathbf{c})x^2+2\mathbf{a}_3^\top\mathbf{b}_2xy\\
&+(\mathbf{b}_2^\top\mathbf{b}_1-\mathbf{a}_3^\top\mathbf{c})x+\mathbf{b}_2^\top\mathbf{b}_2y.
\end{aligned}\right. \tag{10}$$

From the second equation in (10), one can find $y$ as function of $x$

$$y=-\frac{\mathbf{a}_1^\top\mathbf{a}_2x^4+(\mathbf{a}_2^\top\mathbf{a}_3+\mathbf{a}_1^\top\mathbf{b}_1)x^3+(\mathbf{a}_2^\top\mathbf{b}_2+\mathbf{a}_3^\top\mathbf{b}_1-\mathbf{a}_1^\top\mathbf{c})x^2+(\mathbf{b}_2^\top\mathbf{b}_1-\mathbf{a}_3^\top\mathbf{c})x-\mathbf{b}_2^\top\mathbf{c}}{\mathbf{a}_1^\top\mathbf{a}_1x^4+2\mathbf{a}_1^\top\mathbf{a}_3x^3+(\mathbf{a}_3^\top\mathbf{a}_3+2\mathbf{a}_1^\top\mathbf{b}_2)x^2+2\mathbf{a}_3^\top\mathbf{b}_2x+\mathbf{b}_2^\top\mathbf{b}_2}.$$

Inserting (9) into the first equation in (10) gives

$$\begin{aligned}
\mathbf{b}_1^\top\mathbf{c}F_1(x) &= 2\mathbf{a}_2^\top\mathbf{a}_2x^3F_1(x)+2\mathbf{a}_1^\top\mathbf{a}_1x^3F_2(x)-4(\mathbf{a}_1^\top\mathbf{a}_2)x^3F_3(x)\\
&+3\mathbf{a}_2^\top\mathbf{b}_1x^2F_1(x)+3(\mathbf{a}_1^\top\mathbf{a}_3)x^2F_2(x)-3(\mathbf{a}_2^\top\mathbf{a}_3+\mathbf{a}_1^\top\mathbf{b}_1)x^2F_3(x)\\
&+(\mathbf{b}_1^\top\mathbf{b}_1-2\mathbf{a}_2^\top\mathbf{c})xF_1(x)+(\mathbf{a}_3^\top\mathbf{a}_3+2\mathbf{a}_1^\top\mathbf{b}_2)xF_2(x)\\
&-2(\mathbf{a}_2^\top\mathbf{b}_2+\mathbf{a}_3^\top\mathbf{b}_1-\mathbf{a}_1^\top\mathbf{c})xF_3(x)+\mathbf{a}_3^\top\mathbf{b}_2F_2(x)-(\mathbf{b}_1^\top\mathbf{b}_2-\mathbf{a}_3^\top\mathbf{c})F_3(x),
\end{aligned}$$

where

$$\begin{aligned}
F_1(x) &:= \left(\mathbf{a}_1^\top\mathbf{a}_1x^4+2\mathbf{a}_1^\top\mathbf{a}_3x^3+(\mathbf{a}_3^\top\mathbf{a}_3+2\mathbf{a}_1^\top\mathbf{b}_2)x^2+2\mathbf{a}_3^\top\mathbf{b}_2x+\mathbf{b}_2^\top\mathbf{b}_2\right)^2\\
F_2(x) &:= \left(\mathbf{a}_1^\top\mathbf{a}_2x^4+(\mathbf{a}_2^\top\mathbf{a}_3+\mathbf{a}_1^\top\mathbf{b}_1)x^3+(\mathbf{a}_2^\top\mathbf{b}_2+\mathbf{a}_3^\top\mathbf{b}_1-\mathbf{a}_1^\top\mathbf{c})x^2+(\mathbf{b}_2^\top\mathbf{b}_1-\mathbf{a}_3^\top\mathbf{c})x-\mathbf{b}_2^\top\mathbf{c}\right)^2\\
F_3(x) &:= \left(\mathbf{a}_1^\top\mathbf{a}_2x^4+(\mathbf{a}_2^\top\mathbf{a}_3+\mathbf{a}_1^\top\mathbf{b}_1)x^3+(\mathbf{a}_2^\top\mathbf{b}_2+\mathbf{a}_3^\top\mathbf{b}_1-\mathbf{a}_1^\top\mathbf{c})x^2+(\mathbf{b}_2^\top\mathbf{b}_1-\mathbf{a}_3^\top\mathbf{c})x-\mathbf{b}_2^\top\mathbf{c}\right)\cdot\\
&\quad\left(\mathbf{a}_1^\top\mathbf{a}_1x^4+2\mathbf{a}_1^\top\mathbf{a}_3x^3+(\mathbf{a}_3^\top\mathbf{a}_3+2\mathbf{a}_1^\top\mathbf{b}_2)x^2+2\mathbf{a}_3^\top\mathbf{b}_2x+\mathbf{b}_2^\top\mathbf{b}_2\right).
\end{aligned}$$

The coefficients in (8) can be found expanding the equation above. □

**Theorem 5** (Method K2)**.** *Let $\theta_1,\ \theta_2\in[0,\pi)$ and $\theta_1\neq\theta_2$ and $p\in\mathbb{R}_{>0}$. Assume that the apparent impedivity $\widetilde{\kappa}_\mathrm{a}$ can be represented as*

$$\widetilde{\kappa}_\mathrm{a}(\theta):=\kappa_\alpha\left(1-\frac{1}{p((1-\alpha)\varphi(\theta)+\alpha)}\right), \tag{11}$$

*where $\varphi(\theta)$ is a function of $\theta$. Then, the anisotropy ratio $\alpha^2$ satisfies a second degree polynomial*

$$\mu_2\alpha^2 + \mu_1\alpha + \mu_0 = 0 \quad \text{or} \quad \nu_2\alpha^2 + \nu_1\alpha + \nu_0 = 0, \tag{12}$$

*where $\mu_0$, $\mu_1$, $\mu_2$, $\nu_0$, $\nu_1$, $\nu_2$ are defined as*

$$\left\{\begin{array}{lcl} \mu_2 & := & p(1-\varphi(\theta_1))(1-\varphi(\theta_2))(\widetilde{\rho}_{\rm a}(\theta_1)-\widetilde{\rho}_{\rm a}(\theta_2)) \\ \mu_1 & := & p(\widetilde{\rho}_{\rm a}(\theta_1)-\widetilde{\rho}_{\rm a}(\theta_2))(\varphi(\theta_1)+\varphi(\theta_2)-2\varphi(\theta_1)\varphi(\theta_2)) \\ & & -(\widetilde{\rho}_{\rm a}(\theta_1)-\widetilde{\rho}_{\rm a}(\theta_2))+(\widetilde{\rho}_{\rm a}(\theta_1)\varphi(\theta_1)-\widetilde{\rho}_{\rm a}(\theta_2)\varphi(\theta_2)) \\ \mu_0 & := & p\varphi(\theta_1)\varphi(\theta_2)(\widetilde{\rho}_{\rm a}(\theta_1)-\widetilde{\rho}_{\rm a}(\theta_2))-(\widetilde{\rho}_{\rm a}(\theta_1)\varphi(\theta_1)-\widetilde{\rho}_{\rm a}(\theta_2)\varphi(\theta_2)) \\ \nu_2 & := & p(1-\varphi(\theta_1))(1-\varphi(\theta_2))(\widetilde{\tau}_{\rm a}(\theta_1)-\widetilde{\tau}_{\rm a}(\theta_2)) \\ \nu_1 & := & p(\widetilde{\tau}_{\rm a}(\theta_1)-\widetilde{\tau}_{\rm a}(\theta_2))(\varphi(\theta_1)+\varphi(\theta_2)-2\varphi(\theta_1)\varphi(\theta_2)) \\ & & -(\widetilde{\tau}_{\rm a}(\theta_1)-\widetilde{\tau}_{\rm a}(\theta_2))+(\widetilde{\tau}_{\rm a}(\theta_1)\varphi(\theta_1)-\widetilde{\tau}_{\rm a}(\theta_2)\varphi(\theta_2)) \\ \nu_0 & := & p\varphi(\theta_1)\varphi(\theta_2)(\widetilde{\tau}_{\rm a}(\theta_1)-\widetilde{\tau}_{\rm a}(\theta_2))-(\widetilde{\tau}_{\rm a}(\theta_1)\varphi(\theta_1)-\widetilde{\tau}_{\rm a}(\theta_2)\varphi(\theta_2)). \end{array}\right. \tag{13}$$

*Proof.* Splitting the real and imaginary parts of (11), we can have

$$\widetilde{\rho}_{\rm a}(\theta_1)\ p[(1-\alpha)\varphi(\theta_1)+\alpha] = \rho_\alpha\ (p[(1-\alpha)\varphi(\theta_1)+\alpha]-1) \tag{14}$$

$$\widetilde{\rho}_{\rm a}(\theta_2)\ p[(1-\alpha)\varphi(\theta_2)+\alpha] = \rho_\alpha\ (p[(1-\alpha)\varphi(\theta_2)+\alpha]-1) \tag{15}$$

$$\widetilde{\tau}_{\rm a}(\theta_1)\ p[(1-\alpha)\varphi(\theta_1)+\alpha] = \tau_\alpha\ (p[(1-\alpha)\varphi(\theta_1)+\alpha]-1) \tag{16}$$

$$\widetilde{\tau}_{\rm a}(\theta_2)\ p[(1-\alpha)\varphi(\theta_2)+\alpha] = \tau_\alpha\ (p[(1-\alpha)\varphi(\theta_2)+\alpha]-1). \tag{17}$$

The division of (14) by (15) gives

$$\frac{\widetilde{\rho}_{\rm a}(\theta_1)\ [(1-\alpha)\varphi(\theta_1)+\alpha]}{\widetilde{\rho}_{\rm a}(\theta_2)\ [(1-\alpha)\varphi(\theta_2)+\alpha]} = \frac{p[(1-\alpha)\varphi(\theta_1)+\alpha]-1}{p[(1-\alpha)\varphi(\theta_2)+\alpha]-1}.$$

Rearranging the equation above one finds

$$\mu_2\alpha^2 + \mu_1\alpha + \mu_0 = 0.$$

Following the same procedure dividing (16) by (17), we can have

$$\nu_2\alpha^2 + \nu_1\alpha + \nu_0 = 0.$$

□

*Comments on Method K2*

The anisotropy ratio $\alpha^2$ can be computed solving the resistivity or reactivity polynomial in (12). For briefness, we consider below the resistivity polynomial. Note the observations can be applied for the reactivity polynomial replacing $\mu_j$ by $\nu_j$ and $\widetilde{\rho}_{\rm a}(\theta_k)$ by $\widetilde{\tau}_{\rm a}(\theta_k)$ for $j = 0, 1, 2$ and $k = 1, 2$. Based on the degree of the polynomial, the following two cases are possible:

(i) $\mu_2 \neq 0$ (i.e. 2nd degree polynomial). Then, it can be easily shown the following condition must be fulfilled

$$\varphi(\theta_1) \neq 1 \quad \text{and} \quad \varphi(\theta_2) \neq 1.$$

Note that we choose $p > 0$ and $\theta_{\{1,2\}}$ such that $\widetilde{\rho}_{\rm a}(\theta_1) \neq \widetilde{\rho}_{\rm a}(\theta_2)$.

(ii) $\mu_2 = 0$, $\mu_1 \neq 0$ (i.e. 1st degree polynomial). $\mu_2 = 0$ when either $\varphi(\theta_1) = 1$ or $\varphi(\theta_2) = 1$. Without loss of generality, we assume $\varphi(\theta_1) = 1$. Then, to make $\mu_1 \neq 0$ (see Remark 1), we have

$$\widetilde{\rho}_{\mathrm{a}}(\theta_2) \neq \rho_\alpha \quad \text{and} \quad p \neq 1 \quad \text{and} \quad \varphi(\theta_2) \neq 1.$$

Note $\varphi(\theta_2) \neq 1$ because $\varphi(\theta_1) = 1$. Then, choosing $\theta_2$ so that $\widetilde{\rho}_{\mathrm{a}}(\theta_2) = \rho_\alpha$ is unlikely to happen because $\rho_\alpha$ is unknown. Therefore, it needs to be satisfied $p \neq 1$.

**Remark 1.** *The coefficient $a_1$ with $\varphi(\theta_1) = 1$ is*

$$\begin{aligned} a_1 &= p(\widetilde{\rho}_{\mathrm{a}}(\theta_1) - \widetilde{\rho}_{\mathrm{a}}(\theta_2))(1-\varphi_2) - (\widetilde{\rho}_{\mathrm{a}}(\theta_1) - \widetilde{\rho}_{\mathrm{a}}(\theta_2)) + (\widetilde{\rho}_{\mathrm{a}}(\theta_1) - \widetilde{\rho}_{\mathrm{a}}(\theta_2)\varphi_2) \\ &= (p(\rho_{\mathrm{a}}(\theta_1) - \rho_{\mathrm{a}}(\theta_2)) + \rho_{\mathrm{a}}(\theta_2))(1-\varphi_2). \end{aligned}$$

*Note that $\varphi(\theta_1) = 1$ only when $\theta_1 = 0$, thus we have $\widetilde{\rho}_{\mathrm{a}}(\theta_1) = \rho_\alpha(1 - 1/p)$. So, we have $p(\widetilde{\rho}_{\mathrm{a}}(\theta_1) - \widetilde{\rho}_{\mathrm{a}}(\theta_2)) + \widetilde{\rho}_{\mathrm{a}}(\theta_2) = (\rho_\alpha - \widetilde{\rho}_{\mathrm{a}}(\theta_2))(p-1)$. Finally, $a_1$ can be represented as*

$$a_1 = (\rho_\alpha - \widetilde{\rho}_{\mathrm{a}}(\theta_2))(p-1)(1-\varphi_2).$$

*Hence, the following condition needs to fulfilled to make non-zero first degree coefficient, i.e. $a_1 \neq 0$,*

$$\widetilde{\rho}_{\mathrm{a}}(\theta_2) \neq \rho_\alpha \quad \textit{and} \quad p \neq 1 \quad \textit{and} \quad \varphi(\theta_2) \neq 1.$$

**Theorem 6** (Method K3)**.** *Let $\theta_d \in [0, \pi)$ for $d = 1, \ldots, D$ and $D \in \mathbb{N}_{\geq 2}$ the number of angles measured and $p \in \mathbb{R}_{>0}$. Assume that the apparent impedivity $\widetilde{\kappa}_{\mathrm{a}}$ can be represented as*

$$\widetilde{\kappa}_{\mathrm{a}}(\theta) := \kappa_\alpha \left(1 - \frac{1}{p((1-\alpha)\varphi(\theta) + \alpha)}\right), \tag{18}$$

*for $\theta = \theta_1, \theta_2, \ldots, \theta_D$ with a function $\varphi(\theta)$. Then the square root of the anisotropy ratio $\alpha$ and the geometric mean impedivity $\kappa_\alpha$ satisfy the following cases*

*If $\mathbf{a}_{3^\perp} \cdot \mathbf{a}_1 = 0$, $\mathbf{a}_{3^\perp} \cdot \mathbf{a}_2 \neq 0$, then*

$$\alpha^2 = \left(\frac{(\mathbf{a}_{3^\perp} \cdot \mathbf{a}_2)(\mathbf{a}_3 \cdot \mathbf{a}_4) - (\mathbf{a}_{3^\perp} \cdot \mathbf{a}_4)(\mathbf{a}_3 \cdot \mathbf{a}_2)}{(\mathbf{a}_{3^\perp} \cdot \mathbf{a}_2)(\mathbf{a}_3 \cdot \mathbf{a}_1) + (\mathbf{a}_{3^\perp} \cdot \mathbf{a}_4)(\mathbf{a}_3 \cdot \mathbf{a}_3)}\right)^2 \quad \textit{and} \quad \kappa_\alpha = \frac{\mathbf{a}_{3^\perp} \cdot \mathbf{a}_4}{\mathbf{a}_{3^\perp} \cdot \mathbf{a}_2}. \tag{19}$$

*If $\mathbf{a}_{3^\perp} \cdot \mathbf{a}_1 \neq 0$, $\mathbf{a}_{3^\perp} \cdot \mathbf{a}_2 = 0$, then*

$$\alpha^2 = \left(\frac{\mathbf{a}_{3^\perp} \cdot \mathbf{a}_4}{\mathbf{a}_{3^\perp} \cdot \mathbf{a}_1}\right)^2 \quad \textit{and} \quad \kappa_\alpha = \frac{(\mathbf{a}_{3^\perp} \cdot \mathbf{a}_1)(\mathbf{a}_3 \cdot \mathbf{a}_4) - (\mathbf{a}_{3^\perp} \cdot \mathbf{a}_4)(\mathbf{a}_3 \cdot \mathbf{a}_1)}{(\mathbf{a}_{3^\perp} \cdot \mathbf{a}_1)(\mathbf{a}_3 \cdot \mathbf{a}_2) + (\mathbf{a}_{3^\perp} \cdot \mathbf{a}_4)(\mathbf{a}_3 \cdot \mathbf{a}_3)}. \tag{20}$$

*If $\mathbf{a}_{3^\perp} \cdot \mathbf{a}_1 \neq 0$, $\mathbf{a}_{3^\perp} \cdot \mathbf{a}_2 \neq 0$, then*

$$\alpha^2 \in \left\{ \left( \frac{(\mathbf{a}_{3^\perp} \cdot \mathbf{a}_4)(\mathbf{a}_3 \cdot \mathbf{a}_3) - (\mathbf{a}_{3^\perp} \cdot \mathbf{a}_1)(\mathbf{a}_3 \cdot \mathbf{a}_2) + (\mathbf{a}_{3^\perp} \cdot \mathbf{a}_2)(\mathbf{a}_3 \cdot \mathbf{a}_1) \pm \sqrt{T}}{2(\mathbf{a}_{3^\perp} \cdot \mathbf{a}_1)(\mathbf{a}_3 \cdot \mathbf{a}_3)} \right)^2 \right\}$$

*and*

$$\kappa_\alpha \in \left\{ \frac{(\mathbf{a}_{3^\perp} \cdot \mathbf{a}_4)(\mathbf{a}_3 \cdot \mathbf{a}_3) + (\mathbf{a}_{3^\perp} \cdot \mathbf{a}_1)(\mathbf{a}_3 \cdot \mathbf{a}_2) - (\mathbf{a}_{3^\perp} \cdot \mathbf{a}_2)(\mathbf{a}_3 \cdot \mathbf{a}_1) \mp \sqrt{T}}{2(\mathbf{a}_{3^\perp} \cdot \mathbf{a}_2)(\mathbf{a}_3 \cdot \mathbf{a}_3)} \right\}, \tag{21}$$

*where* $\mathbf{a}_1 := [a_{11}, a_{21}, \ldots, a_{D1}]^\top$, $\mathbf{a}_2 := [a_{12}, a_{22}, \ldots, a_{D2}]^\top$, $\mathbf{a}_3 := [a_{13}, a_{23}, \ldots, a_{D3}]^\top$, $\mathbf{a}_4 := [a_{14}, a_{24}, \ldots, a_{D4}]^\top$, $a_{d1} := p\widetilde{\kappa}_{\mathrm{a}}(\theta)(\varphi(\theta_d) - 1)$, $a_{d2} := p\varphi(\theta_d) - 1$, $a_{d3} := -p(\varphi(\theta_d) - 1)$, $a_{d4} := p\widetilde{\kappa}_{\mathrm{a}}(\theta)\varphi(\theta_d)$, *for* $d = 1, \ldots, D$, *and* $T := ((\mathbf{a}_{3^\perp}\cdot\mathbf{a}_4)(\mathbf{a}_3\cdot\mathbf{a}_3) + (\mathbf{a}_{3^\perp}\cdot\mathbf{a}_2)(\mathbf{a}_3\cdot\mathbf{a}_1) + (\mathbf{a}_{3^\perp}\cdot\mathbf{a}_1)(\mathbf{a}_3\cdot\mathbf{a}_2))^2 - 4(\mathbf{a}_{3^\perp}\cdot\mathbf{a}_1)(\mathbf{a}_{3^\perp}\cdot\mathbf{a}_2)\left((\mathbf{a}_3\cdot\mathbf{a}_1)(\mathbf{a}_3\cdot\mathbf{a}_2) + (\mathbf{a}_3\cdot\mathbf{a}_3)(\mathbf{a}_3\cdot\mathbf{a}_4)\right)$ *and where* $\mathbf{a}_{3^\perp}$ *is a vector perpendicular to* $\mathbf{a}_3$.

*Proof.* We can rewrite (18) as

$$p\widetilde{\kappa}_{\mathrm{a}}(\theta)\varphi(\theta) = p\left(\widetilde{\kappa}_{\mathrm{a}}(\theta)\varphi(\theta) - \widetilde{\kappa}_{\mathrm{a}}(\theta)\right)\alpha + \left(p\varphi(\theta) - 1\right)\kappa_\alpha - p\left(\varphi(\theta) - 1\right)\alpha\kappa_\alpha.$$

Let $x := \alpha$ and $y := \kappa_\alpha$. From the definitions of $a_{d1}$, $a_{d2}$, $a_{d3}$, and $a_{d4}$, we can write the equation above as follows

$$\left\{\begin{array}{rcl} a_{11}x_1 + a_{12}x_2 + a_{13}x_1x_2 & = & a_{14} \\ a_{21}x_1 + a_{22}x_2 + a_{23}x_1x_2 & = & a_{24} \\ \vdots & \vdots & \vdots \\ a_{N1}x_1 + a_{N2}x_2 + a_{N3}x_1x_2 & = & a_{N4}. \end{array}\right. \tag{22}$$

The proof is completed by applying the theorem 7 to (22). □

**Theorem 7.** *For* $d = 1, \ldots, D$ *and* $j = 1, 2, 3, 4$ *with* $D \in \mathbb{N}_{\geq 2}$, *the components* $a_{dj} \in \mathbb{R}$ *of the following equations system of* $x_1$ *and* $x_2$ *are given*

$$\left\{\begin{array}{rcl} a_{11}x_1 + a_{12}x_2 + a_{13}x_1x_2 & = & a_{14} \\ a_{21}x_1 + a_{22}x_2 + a_{23}x_1x_2 & = & a_{24} \\ \vdots & \vdots & \vdots \\ a_{D1}x_1 + a_{D2}x_2 + a_{D3}x_1x_2 & = & a_{D4}, \end{array}\right. \tag{23}$$

*with rank*$(\mathbf{M}) \geq 2$ *for* $\mathbf{M} := [\mathbf{a}_1\ \mathbf{a}_2\ \mathbf{a}_3]$. *Here,* $\mathbf{a}_1 := [a_{11}\ a_{21} \ldots\ a_{D1}]^\top$, $\mathbf{a}_2 := [a_{12}\ a_{22} \ldots\ a_{D2}]^\top$, *and* $\mathbf{a}_3 := [a_{13}\ a_{23} \ldots\ a_{D3}]^\top$. *If* $\|\mathbf{a}_3\| = 0$, *then the system is linear, so the solution* $\mathbf{x} := [x_1\ x_2]^\top$ *can be obtained as* $\mathbf{x} = (\mathbf{A}^\top\mathbf{A})^{-1}\mathbf{A}^\top\mathbf{a}_4$ *where* $\mathbf{A} := [\mathbf{a}_1\ \mathbf{a}_2]$ *and* $\mathbf{a}_4 := [a_{14}\ a_{24} \ldots\ a_{D4}]^\top$. *In the case when system is non-linear, i.e.* $\|\mathbf{a}_3\| \neq 0$, *then the solution* $x_1$ *and* $x_2$ *satisfy that*

*if* $\mathbf{a}_{3^\perp}\cdot\mathbf{a}_1 = 0$, $\mathbf{a}_{3^\perp}\cdot\mathbf{a}_2 \neq 0$,

$$x_1 = \frac{(\mathbf{a}_{3^\perp}\cdot\mathbf{a}_2)(\mathbf{a}_3\cdot\mathbf{a}_4) - (\mathbf{a}_{3^\perp}\cdot\mathbf{a}_4)(\mathbf{a}_3\cdot\mathbf{a}_2)}{(\mathbf{a}_{3^\perp}\cdot\mathbf{a}_2)(\mathbf{a}_3\cdot\mathbf{a}_1) + (\mathbf{a}_{3^\perp}\cdot\mathbf{a}_4)(\mathbf{a}_3\cdot\mathbf{a}_3)} \quad \textit{and} \quad x_2 = \frac{(\mathbf{a}_{3^\perp}\cdot\mathbf{a}_4)}{(\mathbf{a}_{3^\perp}\cdot\mathbf{a}_2)}. \tag{24}$$

*if* $\mathbf{a}_{3^\perp}\cdot\mathbf{a}_1 \neq 0$, $\mathbf{a}_{3^\perp}\cdot\mathbf{a}_2 = 0$,

$$x_1 = \frac{(\mathbf{a}_{3^\perp}\cdot\mathbf{a}_4)}{(\mathbf{a}_{3^\perp}\cdot\mathbf{a}_1)} \quad \textit{and} \quad x_2 = \frac{(\mathbf{a}_{3^\perp}\cdot\mathbf{a}_1)(\mathbf{a}_3\cdot\mathbf{a}_4) - (\mathbf{a}_{3^\perp}\cdot\mathbf{a}_4)(\mathbf{a}_3\cdot\mathbf{a}_1)}{(\mathbf{a}_{3^\perp}\cdot\mathbf{a}_1)(\mathbf{a}_3\cdot\mathbf{a}_2) + (\mathbf{a}_{3^\perp}\cdot\mathbf{a}_4)(\mathbf{a}_3\cdot\mathbf{a}_3)}. \tag{25}$$

*if* $\mathbf{a}_{3^\perp}\cdot\mathbf{a}_1 \neq 0$, $\mathbf{a}_{3^\perp}\cdot\mathbf{a}_2 \neq 0$,

$$x_1 \in \left\{\frac{(\mathbf{a}_{3^\perp}\cdot\mathbf{a}_4)(\mathbf{a}_3\cdot\mathbf{a}_3) - (\mathbf{a}_{3^\perp}\cdot\mathbf{a}_1)(\mathbf{a}_3\cdot\mathbf{a}_2) + (\mathbf{a}_{3^\perp}\cdot\mathbf{a}_2)(\mathbf{a}_3\cdot\mathbf{a}_1) \pm \sqrt{T}}{2(\mathbf{a}_{3^\perp}\cdot\mathbf{a}_1)(\mathbf{a}_3\cdot\mathbf{a}_3)}\right\}$$

*and*

$$x_2 \in \left\{ \frac{(\mathbf{a}_{3^\perp} \cdot \mathbf{a}_4)(\mathbf{a}_3 \cdot \mathbf{a}_3) + (\mathbf{a}_{3^\perp} \cdot \mathbf{a}_1)(\mathbf{a}_3 \cdot \mathbf{a}_2) - (\mathbf{a}_{3^\perp} \cdot \mathbf{a}_2)(\mathbf{a}_3 \cdot \mathbf{a}_1) \mp \sqrt{T}}{2(\mathbf{a}_{3^\perp} \cdot \mathbf{a}_2)(\mathbf{a}_3 \cdot \mathbf{a}_3)} \right\}, \quad (26)$$

*where* $T := ((\mathbf{a}_{3^\perp} \cdot \mathbf{a}_4)(\mathbf{a}_3 \cdot \mathbf{a}_3) + (\mathbf{a}_{3^\perp} \cdot \mathbf{a}_2)(\mathbf{a}_3 \cdot \mathbf{a}_1) + (\mathbf{a}_{3^\perp} \cdot \mathbf{a}_1)(\mathbf{a}_3 \cdot \mathbf{a}_2))^2 - 4(\mathbf{a}_{3^\perp} \cdot \mathbf{a}_1)(\mathbf{a}_{3^\perp} \cdot \mathbf{a}_2)\left((\mathbf{a}_3 \cdot \mathbf{a}_1)(\mathbf{a}_3 \cdot \mathbf{a}_2) + (\mathbf{a}_3 \cdot \mathbf{a}_3)(\mathbf{a}_3 \cdot \mathbf{a}_4)\right)$ *and where* $\mathbf{a}_{3^\perp}$ *is a vector perpendicular to* $\mathbf{a}_3$*.*

*Proof.* We can formulate (23) as a summation of two systems, namely

$$\begin{bmatrix} \mathbf{a}_3 \end{bmatrix} \begin{bmatrix} x_1 x_2 \end{bmatrix} + \begin{bmatrix} \mathbf{a}_1 & \mathbf{a}_2 \end{bmatrix} \begin{bmatrix} x_1 \\ x_2 \end{bmatrix} = \begin{bmatrix} \mathbf{a}_4 \end{bmatrix}. \quad (27)$$

Since the term $\mathbf{a}_3$ is non-zero, we can consider the orthogonal vector $\mathbf{a}_{3^\perp}$ of $\mathbf{a}_3$. Then, the multiplication of $\mathbf{a}_3$ and $\mathbf{a}_{3^\perp}$ to (27) gives

$$\begin{cases} \|\mathbf{a}_3\|^2 x_1 x_2 + [\mathbf{a}_3 \cdot \mathbf{a}_1 \quad \mathbf{a}_3 \cdot \mathbf{a}_2]\,[x_1 \quad x_2]^\top = \mathbf{a}_3 \cdot \mathbf{a}_4 \\ [\mathbf{a}_{3^\perp} \cdot \mathbf{a}_1 \quad \mathbf{a}_{3^\perp} \cdot \mathbf{a}_2]\,[x_1 \quad x_2]^\top = \mathbf{a}_{3^\perp} \cdot \mathbf{a}_4, \end{cases}$$

or

$$\|\mathbf{a}_3\|^2 x_1 x_2 + \mathbf{a}_3 \cdot \mathbf{a}_1 x_1 + \mathbf{a}_3 \cdot \mathbf{a}_2 x_2 = \mathbf{a}_3 \cdot \mathbf{a}_4 \quad (28)$$

$$\mathbf{a}_{3^\perp} \cdot \mathbf{a}_1 x_1 + \mathbf{a}_{3^\perp} \cdot \mathbf{a}_2 x_2 = \mathbf{a}_{3^\perp} \cdot \mathbf{a}_4. \quad (29)$$

If $\mathbf{a}_{3^\perp} \cdot \mathbf{a}_1 = 0$ and $\mathbf{a}_{3^\perp} \cdot \mathbf{a}_2 \neq 0$, then from (29) we have that

$$x_2 = \frac{\mathbf{a}_{3^\perp} \cdot \mathbf{a}_4}{\mathbf{a}_{3^\perp} \cdot \mathbf{a}_2}. \quad (30)$$

Inserting (30) into (28) gives us $x_1$

$$x_1 = \frac{(\mathbf{a}_{3^\perp} \cdot \mathbf{a}_2)(\mathbf{a}_3 \cdot \mathbf{a}_4) - (\mathbf{a}_3 \cdot \mathbf{a}_2)(\mathbf{a}_{3^\perp} \cdot \mathbf{a}_4)}{\|\mathbf{a}_3\|^2(\mathbf{a}_{3^\perp} \cdot \mathbf{a}_4) + (\mathbf{a}_{3^\perp} \cdot \mathbf{a}_2)(\mathbf{a}_3 \cdot \mathbf{a}_1)}.$$

On the other hand, if $\mathbf{a}_{3^\perp} \cdot \mathbf{a}_2 = 0$ and $\mathbf{a}_{3^\perp} \cdot \mathbf{a}_1 \neq 0$, then from (29) we have that

$$x_1 = \frac{\mathbf{a}_{3^\perp} \cdot \mathbf{a}_4}{\mathbf{a}_{3^\perp} \cdot \mathbf{a}_1}. \quad (31)$$

Inserting (31) into (28) gives us $x_2$

$$x_2 = \frac{(\mathbf{a}_{3_j^\perp} \cdot \mathbf{a}_1)(\mathbf{a}_3 \cdot \mathbf{a}_4) - (\mathbf{a}_3 \cdot \mathbf{a}_1)(\mathbf{a}_{3_j^\perp} \cdot \mathbf{a}_4)}{\|\mathbf{a}_3\|^2(\mathbf{a}_{3_j^\perp} \cdot \mathbf{a}_4) + (\mathbf{a}_{3_j^\perp} \cdot \mathbf{a}_1)(\mathbf{a}_3 \cdot \mathbf{a}_2)}.$$

Finally, if $\mathbf{a}_{3^\perp} \cdot \mathbf{a}_1 \neq 0$ and $\mathbf{a}_{3^\perp} \cdot \mathbf{a}_2 \neq 0$, then from (29) we have that

$$x_2 = \frac{\mathbf{a}_{3^\perp} \cdot \mathbf{a}_4 - \mathbf{a}_{3^\perp} \cdot \mathbf{a}_1 x_1}{\mathbf{a}_{3^\perp} \cdot \mathbf{a}_2}. \quad (32)$$

Inserting (32) into (28) will give us $x_1$

$$p_2 x_1^2 + p_1 x_1 + p_0 = 0,$$

where $p_2 := \|\mathbf{a}_3\|^2(\mathbf{a}_{3_j^\perp} \cdot \mathbf{a}_1)$, $p_1 := -\|\mathbf{a}_3\|^2(\mathbf{a}_{3_j^\perp} \cdot \mathbf{a}_4) - (\mathbf{a}_{3_j^\perp} \cdot \mathbf{a}_2)(\mathbf{a}_3 \cdot \mathbf{a}_1) + (\mathbf{a}_3 \cdot \mathbf{a}_2)(\mathbf{a}_{3_j^\perp} \cdot \mathbf{a}_1)$ and $p_0 := (\mathbf{a}_{3_j^\perp} \cdot \mathbf{a}_2)(\mathbf{a}_3 \cdot \mathbf{a}_4) - (\mathbf{a}_3 \cdot \mathbf{a}_2)(\mathbf{a}_{3_j^\perp} \cdot \mathbf{a}_4)$. Solving the second order polynomial above will give us $x_1$. Inserting $x_1$ into (32) will give us $x_2$. □

*Comments on Method K3*

If one applies to method K3 the same approach used in method H3, the anisotropy ratio $\alpha^2$ and the geometric mean impedivity $\kappa_\alpha$ are solution of a 5th degree polynomial. We found in this case that, on top of the approximation error of the method, both $\alpha^2$ and $\kappa_\alpha$ were largely affected by measurement noise. Instead, we solve the non-linear system after multiplying by $\mathbf{a}_3$ and $\mathbf{a}_{3\perp}$ (see Theorem in section 7 for further details).

**Part C. Multiple inter-electrodes' distances within the same needle and one measurement angle**

**Theorem 8** (Method B1)**.** *Assume that we measure apparent impedivity $\kappa_{\mathrm{a}}(p_n)$ for $n = 1, \ldots, N$ and $N = 2$ with $\theta \in [0, \pi)$ as shown in (1) and $p_1 \neq p_2 > 0$. Then, the geometric mean impedivity $\kappa_\alpha$ can be obtained as*

$$\kappa_\alpha = \frac{\kappa_{\mathrm{a}}(p_1)p_1 - \kappa_{\mathrm{a}}(p_2)p_2}{p_1 - p_2}, \tag{33}$$

*where $p_n := g/h_n$ for $n = 1, 2$. The anisotropy ratio $\alpha^2$ can be obtained as follows*

$$\alpha^2 = (\mathbf{q}^\top \mathbf{q})^{-1} \mathbf{q}^\top \mathbf{d}, \tag{34}$$

*where $\mathbf{q} := [p_1^2(\kappa_\alpha - \kappa_{\mathrm{a}}(p_1))^2 \sin^2\theta,\ p_2^2(\kappa_\alpha - \kappa_{\mathrm{a}}(p_2))^2 \sin^2\theta]^\top$ and $\mathbf{d} := [\kappa_\alpha^2 - p_1^2(\kappa_\alpha - \kappa_{\mathrm{a}}(p_1))^2 \cos^2\theta,\ \kappa_\alpha^2 - p_2^2(\kappa_\alpha - \kappa_{\mathrm{a}}(p_2))^2 \cos^2\theta]^\top$.*

*Proof.* The difference of two apparent impedivity $\kappa_{\mathrm{a},n}$ multiplied by $p_n$ for $n = 1, 2$ gives

$$\kappa_{\mathrm{a}}(p_1)p_1 - \kappa_{\mathrm{a}}(p_2)p_2 = \kappa_\alpha(p_1 - p_2).$$

Then, we can represent the geometric mean impedivity $\kappa_\alpha = \alpha\kappa_{\mathrm{T}}$ as

$$\kappa_\alpha = \frac{\kappa_{\mathrm{a}}(p_1)p_1 - \kappa_{\mathrm{a}}(p_2)p_2}{p_1 - p_2}. \tag{35}$$

To obtain $\alpha^2$, we have from (1) that

$$\frac{\kappa_{\mathrm{a}}(p_n)}{\kappa_\alpha} = 1 - \frac{1}{p_n\sqrt{\cos^2\theta + \alpha^2 \sin^2\theta}} \quad \text{for } n = 1, 2,$$

which implies that

$$g^2(\kappa_\alpha - \kappa_{\mathrm{a}}(p_n))^2 \sin^2\theta\alpha^2 = \kappa_\alpha^2 - p_n^2(\kappa_\alpha - \kappa_{\mathrm{a}}(p_n))^2 \cos^2\theta.$$

Then, we have the following linear system as function of $\alpha^2$

$$\begin{bmatrix} p_1^2(\kappa_\alpha - \kappa_{\mathrm{a}}(p_1))^2 \sin^2\theta \\ p_2^2(\kappa_\alpha - \kappa_{\mathrm{a}}(p_2))^2 \sin^2\theta \end{bmatrix} \alpha^2 = \begin{bmatrix} \kappa_\alpha^2 - p_1^2(\kappa_\alpha - \kappa_{\mathrm{a}}(p_1))^2 \cos^2\theta \\ \kappa_\alpha^2 - p_2^2(\kappa_\alpha - \kappa_{\mathrm{a}}(p_2))^2 \cos^2\theta \end{bmatrix}.$$

□

**Theorem 9** (Method B2)**.** *Assume that we measure apparent impedivity $\kappa_{\rm a}(p_n)$ for $n = 1, \ldots, N$ and $N \in \mathbb{N}_{\geq 2}$ with $\theta \in [0, \pi)$ as shown in (1). Then the following linear system holds for $x := \kappa_\alpha$ and $y := \kappa_\alpha/\sqrt{\cos^2\theta + \alpha^2 \sin^2\theta}$*

$$\begin{bmatrix} 1 & -1/p_1 \\ 1 & -1/p_2 \\ \vdots & \vdots \\ 1 & -1/p_N \end{bmatrix} \begin{bmatrix} x \\ y \end{bmatrix} = \begin{bmatrix} \kappa_{\rm a}(p_1) \\ \kappa_{\rm a}(p_2) \\ \vdots \\ \kappa_{\rm a}(p_N) \end{bmatrix}. \tag{36}$$

*where the anisotropy ratio $\alpha^2$ can be calculated as follows*

$$\alpha^2 = (x^2/y^2 - \cos^2\theta)/\sin^2\theta.$$

*Proof.* Equation (1) can be written as

$$\kappa_{\rm a}(p_n) = x - \frac{y}{p_n}.$$

Hence, for $n = 1, \ldots, N$, we have the linear system in (36). □

**Part D. Multiple inter-electrodes' distances within the same needle and multiple measurement angles**

**Theorem 10** (Method S1)**.** *Let $\theta_d \in [0,\ \pi)$ for $d = 1, \ldots, D$, $D \in \mathbb{N}_{\geq 3}$ and $p_n \in \mathbb{R}_{>0}$ for $n = 1, \ldots, N$, $N \in \mathbb{N}_{\geq 2}$. Then, the geometric mean impedivity $\kappa_\alpha$ can be calculated using the apparent impedivity $\kappa_{\rm a}(\theta, p)$ in (1) as follows*

$$\kappa_\alpha = [1\ 0\ 0 \ldots\ 0](\mathbf{S}^\top \mathbf{S})^{-1} \mathbf{S}^\top \mathbf{s},$$

*where $\mathbf{s} := [\mathbf{s}_1\ \mathbf{s}_2 \ldots \mathbf{s}_D]^\top$ and $\mathbf{s}_d := [\kappa_{\rm a}(\theta_d, p_1)\ \kappa_{\rm a}(\theta_d, p_2)\ \ldots\ \kappa_{\rm a}(\theta_d, p_N)]$ for $d = 1, \ldots, D$ and $\mathbf{S}$ is an augmented matrix of size $ND \times (D+1)$ defined as*

$$\mathbf{S} := \begin{bmatrix} \mathbf{S}_1 \\ \mathbf{S}_2 \\ \vdots \\ \mathbf{S}_D \end{bmatrix} \quad \textit{with} \quad \mathbf{S}_d := \begin{bmatrix} \mathbf{1} & \mathbf{p}_{d,1} & \mathbf{p}_{d,2} & \ldots & \mathbf{p}_{d,t} & \ldots & \mathbf{p}_{d,D} \end{bmatrix}$$

*a block matrix of size $N \times (D+1)$ made of column vectors $\mathbf{1} := [1\ 1\ \ldots\ 1]^\top$ and $\mathbf{p}_{d,t} := \delta_{d,t}[-1/p_1\ \ -1/p_2\ \ldots\ \ -1/p_N]^\top$ with $\delta_{d,t} := 1$ if $t = d$ else 0, where $^\top$ denotes the transpose operator. Then, the anisotropy ratio $\alpha^2$ and the anisotropy direction $\xi$ can be calculated from the apparent impedivity $\kappa_{\rm a}(\theta, p)$ in (1), i.e.*

$$\begin{aligned} \alpha^2 &= 1 - \varpi_1 &&= 1 - \sqrt{\varpi_2^2 + \varpi_3^2} \\ \xi &= \tfrac{1}{2}\sin^{-1}\tfrac{\varpi_2}{\varpi_1} &&= \tfrac{1}{2}\tan^{-1}\tfrac{\varpi_2}{\varpi_3} &&= \tfrac{1}{2}\cos^{-1}\tfrac{\varpi_3}{\varpi_1}, \end{aligned}$$

*where $\varpi_1$, $\varpi_2$, and $\varpi_3$ are the solution of the linear system*

$$\begin{bmatrix} 1 & -\sin 2\theta_1 & -\cos 2\theta_1 \\ 1 & -\sin 2\theta_2 & -\cos 2\theta_2 \\ \vdots & \vdots & \vdots \\ 1 & -\sin 2\theta_D & -\cos 2\theta_D \end{bmatrix} \begin{bmatrix} \varpi_1 \\ \varpi_2 \\ \varpi_3 \end{bmatrix} = \begin{bmatrix} 2-2/q_1 \\ 2-2/q_2 \\ \vdots \\ 2-2/q_D \end{bmatrix},$$

*with* $q_d := p_n^2 \left( \frac{\kappa_{\mathrm{a}}(\theta_d, p_n)}{\kappa_\alpha} - 1 \right)^2$ *for* $n = 1, \dots, N$.

*Proof.* Equation (1) can be written as

$$m_{d,n} = \kappa_\alpha - \frac{y_d}{p_n},$$

where $m_{d,n} := \kappa_{\mathrm{a}}(\theta_d, p_n)$ and $y_d := \kappa_\alpha / \sqrt{\cos^2(\theta_d - \xi) + \alpha^2 \sin^2(\theta_d - \xi)}$. If we consider multiple inter-electrodes' distances $n = 1, \dots, N$ and fixed measurement angle $d \in \{1, \dots, D\}$, we have that

$$\begin{bmatrix} 1 & -1/p_1 \\ 1 & -1/p_2 \\ \vdots & \vdots \\ 1 & -1/p_N \end{bmatrix} \begin{bmatrix} \kappa_\alpha \\ y_d \end{bmatrix} = \begin{bmatrix} m_{d,1} \\ m_{d,2} \\ \vdots \\ m_{d,N} \end{bmatrix}. \tag{37}$$

If we extend (37) to all $d = 1, \dots, D$, we have the following linear system

$$\begin{bmatrix} \mathbf{S}_1 \\ \mathbf{S}_2 \\ \vdots \\ \mathbf{S}_D \end{bmatrix} \begin{bmatrix} \kappa_\alpha \\ y_1 \\ y_2 \\ \vdots \\ y_D \end{bmatrix} = \begin{bmatrix} \mathbf{s}_1 \\ \mathbf{s}_2 \\ \vdots \\ \mathbf{s}_D \end{bmatrix},$$

where $\mathbf{s}_d = [m_{d,1}\ m_{d,2}\ \dots\ m_{d,N}]^\top$ for $d = 1, \dots, D$ and the block matrix $\mathbf{S}_d$ of size $N \times (D+1)$ is

$$\mathbf{S}_d := \begin{bmatrix} \mathbf{1} & \mathbf{p}_{d,1} & \mathbf{p}_{d,2} & \dots & \mathbf{p}_{d,t} & \dots & \mathbf{p}_{d,D} \end{bmatrix},$$

with the column vectors $\mathbf{1} := [1\ 1\ \dots\ 1]^\top$ and $\mathbf{p}_{d,t} := \delta_{d,t}[-1/p_{d,1}\ -1/p_{d,2}\ \dots\ -1/p_{d,N}]^\top$ for $\delta_{d,t} := 1$ if $t = d$ else 0. Then, $\kappa_\alpha$ is the first component of $(\mathbf{S}^\top \mathbf{S})^{-1} \mathbf{S}^\top \mathbf{s}$ for $\mathbf{s} = [\mathbf{s}_1\ \mathbf{s}_2 \dots\ \mathbf{s}_D]^\top$.

Now, consider multiple measurement angles $d = 1, \dots, D$ and fixed inter-electrodes distance $n \in \{1, \dots, N\}$. Then from (1), we have

$$\left( \frac{m_{dn}}{\kappa_\alpha} - 1 \right)^2 = \frac{1}{p_n^2 (\cos^2(\theta_d - \xi) + \alpha^2 \sin^2(\theta_d - \xi))}.$$

Note that $q_d$ does not depend on $n$. Then, by the trigonometric rule, we have

$$(1-\alpha^2) - \cos 2\theta_d (1-\alpha^2)\cos 2\xi - \sin 2\theta_d (1-\alpha^2)\sin 2\xi = 2 - 2/q_d.$$

From the equation above, we can derive the following linear system

$$\begin{bmatrix} 1 & -\sin 2\theta_1 & -\cos 2\theta_1 \\ 1 & -\sin 2\theta_2 & -\cos 2\theta_2 \\ \vdots & \vdots & \vdots \\ 1 & -\sin 2\theta_D & -\cos 2\theta_D \end{bmatrix} \begin{bmatrix} \varpi_1 \\ \varpi_2 \\ \varpi_3 \end{bmatrix} = \begin{bmatrix} 2-2/q_1 \\ 2-2/q_2 \\ \vdots \\ 2-2/q_D \end{bmatrix},$$

where $\varpi_1 := 1-\alpha^2$, $\varpi_2 := (1-\alpha^2)\sin 2\xi$, and $\varpi_3 := (1-\alpha^2)\cos 2\xi$. Finally, the anisotropy ratio $\alpha^2$ and the anisotropy direction $\xi$ satisfy

$$\begin{aligned} \alpha^2 &= 1-\varpi_1 &&= 1-\sqrt{\varpi_2^2+\varpi_3^2} \\ \xi &= \tfrac{1}{2}\sin^{-1}\tfrac{\varpi_2}{\varpi_1} &&= \tfrac{1}{2}\tan^{-1}\tfrac{\varpi_2}{\varpi_3} &&= \tfrac{1}{2}\cos^{-1}\tfrac{\varpi_3}{\varpi_1}. \end{aligned}$$

□

## Part E. Needle manufacturing

The needle devices were made of stainless steel as conductor and polyimide and medical grade epoxylite varnish as insulators. The body of the needle was formed using a 24 gauge (nominal outer diameter 0.566 mm) stainless steel tube to which a steel wire was laser weld at one end. The wire was cut to length and shaped into a conical tip with an apex angle of 30°. The body thus formed, was dipped into epoxylite varnish and baked at about 180 °C for 30 minutes to cure the epoxylite. A polyimide tubing was placed over the body of the needle as a second insulation layer. Impedance electrodes were made from 19 gauge stainless steel tubing which were 1 mm wide. Platinum–iridium wires (75 $\mu$m in diameter) were soldered on the side of the collar before the collars. The wires were then thread through the body while assembling the collar onto the body. The collars were then glued in place and spaced using precut polyimide tubes. This assembly was again dipped in epoxylite and baked at 180 °C for 30 minutes. Epoxylite filled any remaining gaps between the contacts and the polyimide tubes to create a uniform surface measuring about 19 gauge outer diameter. Needle electrode contacts were exposed by manually scraping off the epoxylite from the surface of the contacts. The distance between the edges of the inner and outer electrodes was 0.5 mm while the distance between the edges of the inner electrodes was 1 mm. The width and height of the ring-shaped electrodes around the entire needle's shaft was 1 mm and 0.0254 mm, respectively. All electrodes were tested to ensure continuity with the corresponding contact available in the connector.

## 1. References

Kwon H, Nagy J A, Taylor R, Rutkove S B & Sanchez B 2017 *Phys. Med. Biol.* **62**(22), 8616–8633.

## Part E. Dielectric data

**Supplementary table 1.** Estimated conductivity $\widehat{\sigma}$ and relative permittivity $\widehat{\varepsilon}_r$ in longitudinal (L) and transverse (T) directions measured in situ using method S1 (see section 5.1). Mean and standard deviation (std) are reported from $M = 3$ measurements on ovine medius gluteus tissue ($n = 3$). Units: frequency $f$, kHz; conductivity, S m$^{-1}$; relative permittivity, dimensionless.

| $f$ | $\widehat{\sigma}_{\mathrm{L,mean}}$ | $\widehat{\sigma}_{\mathrm{L,std}}$ | $\widehat{\varepsilon}_{r,\mathrm{L,mean}}$ | $\widehat{\varepsilon}_{r,\mathrm{L,std}}$ | $\widehat{\sigma}_{\mathrm{T,mean}}$ | $\widehat{\sigma}_{\mathrm{T,std}}$ | $\widehat{\varepsilon}_{r,\mathrm{T,mean}}$ | $\widehat{\varepsilon}_{r,\mathrm{T,std}}$ |
|---|---|---|---|---|---|---|---|---|
| 30.37 | 0.031 | 0 | 3921 | 136 | 0.029 | 0 | 4412 | 232 |
| 31.06 | 0.031 | 0 | 4361 | 182 | 0.029 | 0 | 4669 | 230 |
| 31.79 | 0.031 | 0 | 4325 | 155 | 0.029 | 0 | 4712 | 230 |
| 32.51 | 0.032 | 0 | 3860 | 103 | 0.029 | 0 | 4469 | 246 |
| 33.23 | 0.032 | 0 | 4407 | 160 | 0.030 | 0 | 4686 | 203 |
| 34.02 | 0.032 | 0 | 3870 | 80 | 0.030 | 0 | 4489 | 229 |
| 34.78 | 0.032 | 0 | 4630 | 157 | 0.030 | 0 | 4870 | 194 |
| 35.59 | 0.032 | 0 | 4015 | 107 | 0.030 | 0 | 4463 | 191 |
| 36.41 | 0.032 | 0 | 4183 | 106 | 0.030 | 0 | 4556 | 175 |
| 37.23 | 0.033 | 0 | 4470 | 131 | 0.031 | 0 | 4749 | 177 |
| 38.11 | 0.033 | 0 | 4372 | 113 | 0.031 | 0 | 4669 | 165 |
| 38.96 | 0.033 | 0 | 4481 | 112 | 0.031 | 0 | 4756 | 162 |
| 39.87 | 0.033 | 0 | 4456 | 108 | 0.031 | 0 | 4724 | 155 |
| 40.79 | 0.034 | 0 | 4488 | 101 | 0.032 | 0 | 4740 | 143 |
| 41.73 | 0.034 | 0 | 4449 | 81 | 0.032 | 0 | 4713 | 128 |
| 42.68 | 0.034 | 0 | 4456 | 79 | 0.032 | 0 | 4714 | 122 |
| 43.65 | 0.035 | 0 | 4462 | 84 | 0.033 | 0 | 4713 | 125 |
| 44.66 | 0.035 | 0 | 4390 | 88 | 0.033 | 0 | 4628 | 120 |
| 45.66 | 0.035 | 0 | 4466 | 80 | 0.033 | 0 | 4696 | 117 |
| 46.73 | 0.035 | 0 | 4489 | 75 | 0.033 | 0 | 4687 | 106 |
| 47.81 | 0.036 | 0 | 4389 | 65 | 0.034 | 0 | 4635 | 100 |
| 48.91 | 0.036 | 0 | 4576 | 70 | 0.034 | 0 | 4749 | 99 |
| 50.01 | 0.036 | 0 | 4504 | 55 | 0.035 | 0 | 4708 | 87 |
| 51.17 | 0.036 | 0 | 4476 | 52 | 0.035 | 0 | 4660 | 82 |
| 52.34 | 0.037 | 0 | 4598 | 56 | 0.035 | 0 | 4743 | 82 |
| 53.53 | 0.037 | 0 | 4526 | 46 | 0.036 | 0 | 4691 | 72 |
| 54.76 | 0.037 | 0 | 4396 | 33 | 0.036 | 0 | 4574 | 59 |
| 56.02 | 0.038 | 0 | 4390 | 29 | 0.036 | 0 | 4561 | 54 |
| 57.31 | 0.038 | 0 | 4534 | 23 | 0.036 | 0 | 4663 | 46 |
| 58.63 | 0.039 | 0 | 4417 | 14 | 0.037 | 0 | 4570 | 36 |
| 59.98 | 0.039 | 0 | 4388 | 18 | 0.037 | 0 | 4544 | 36 |
| 61.34 | 0.040 | 0 | 4510 | 15 | 0.038 | 0 | 4651 | 31 |
| 62.75 | 0.040 | 0 | 4296 | 14 | 0.038 | 0 | 4462 | 22 |
| 64.20 | 0.040 | 0 | 4300 | 25 | 0.038 | 0 | 4446 | 22 |
| 65.68 | 0.040 | 0 | 4203 | 23 | 0.038 | 0 | 4343 | 17 |
| 67.19 | 0.041 | 0 | 4160 | 24 | 0.039 | 0 | 4328 | 17 |
| 68.73 | 0.041 | 0 | 4230 | 33 | 0.040 | 0 | 4368 | 22 |
| 70.31 | 0.041 | 0 | 4223 | 41 | 0.040 | 0 | 4349 | 28 |
| 71.91 | 0.042 | 0 | 4159 | 51 | 0.040 | 0 | 4297 | 39 |
| 73.58 | 0.043 | 0 | 4178 | 60 | 0.041 | 0 | 4303 | 46 |

| $f$ | $\widehat{\sigma}_{\mathrm{L,mean}}$ | $\widehat{\sigma}_{\mathrm{L,std}}$ | $\widehat{\varepsilon}_{r,\mathrm{L,mean}}$ | $\widehat{\varepsilon}_{r,\mathrm{L,std}}$ | $\widehat{\sigma}_{\mathrm{T,mean}}$ | $\widehat{\sigma}_{\mathrm{T,std}}$ | $\widehat{\varepsilon}_{r,\mathrm{T,mean}}$ | $\widehat{\varepsilon}_{r,\mathrm{T,std}}$ |
|---|---|---|---|---|---|---|---|---|
| 75.25 | 0.043 | 0 | 4202 | 72 | 0.042 | 0 | 4320 | 56 |
| 76.98 | 0.044 | 0 | 4091 | 76 | 0.042 | 0 | 4218 | 63 |
| 78.74 | 0.044 | 0 | 4004 | 83 | 0.042 | 0 | 4142 | 72 |
| 80.57 | 0.044 | 0 | 4103 | 93 | 0.043 | 0 | 4216 | 77 |
| 82.39 | 0.045 | 0 | 4027 | 100 | 0.043 | 0 | 4150 | 86 |
| 84.28 | 0.045 | 0 | 3991 | 110 | 0.044 | 0 | 4108 | 97 |
| 86.23 | 0.046 | 0 | 3921 | 110 | 0.044 | 0 | 4052 | 100 |
| 88.21 | 0.046 | 0 | 3971 | 118 | 0.045 | 0 | 4073 | 101 |
| 90.23 | 0.047 | 0 | 3786 | 123 | 0.045 | 0 | 3913 | 115 |
| 92.31 | 0.048 | 0 | 3827 | 134 | 0.046 | 0 | 3947 | 125 |
| 94.41 | 0.048 | 0 | 3709 | 139 | 0.047 | 0 | 3835 | 134 |
| 96.59 | 0.049 | 0 | 3704 | 142 | 0.047 | 0 | 3825 | 134 |
| 98.82 | 0.049 | 0 | 3825 | 164 | 0.047 | 0 | 3918 | 146 |
| 101.09 | 0.049 | 0 | 3722 | 165 | 0.048 | 0 | 3818 | 151 |
| 103.38 | 0.050 | 0 | 3622 | 163 | 0.049 | 0 | 3730 | 154 |
| 105.78 | 0.050 | 0 | 3646 | 172 | 0.049 | 0 | 3737 | 162 |
| 108.20 | 0.051 | 0 | 3564 | 178 | 0.049 | 0 | 3657 | 170 |
| 110.68 | 0.051 | 0 | 3587 | 180 | 0.050 | 0 | 3668 | 175 |
| 113.23 | 0.052 | 0 | 3539 | 195 | 0.050 | 0 | 3618 | 192 |
| 115.81 | 0.053 | 0 | 3500 | 199 | 0.051 | 0 | 3577 | 200 |
| 118.49 | 0.054 | 0 | 3408 | 199 | 0.052 | 0 | 3487 | 207 |
| 121.20 | 0.055 | 0 | 3407 | 200 | 0.053 | 0 | 3479 | 209 |
| 123.97 | 0.051 | 0 | 3110 | 187 | 0.050 | 0 | 3163 | 195 |
| 126.83 | 0.055 | 0 | 3250 | 208 | 0.054 | 0 | 3318 | 224 |
| 129.72 | 0.057 | 0 | 3355 | 223 | 0.055 | 0 | 3414 | 238 |
| 132.71 | 0.057 | 0 | 3190 | 216 | 0.055 | 0 | 3253 | 239 |
| 135.77 | 0.057 | 0 | 3184 | 226 | 0.056 | 0 | 3238 | 247 |
| 138.88 | 0.058 | 0 | 3180 | 238 | 0.056 | 0 | 3226 | 259 |
| 142.06 | 0.058 | 0 | 3078 | 235 | 0.057 | 0 | 3125 | 260 |
| 145.33 | 0.059 | 0 | 3007 | 230 | 0.058 | 0 | 3060 | 263 |
| 148.67 | 0.060 | 0 | 3027 | 246 | 0.058 | 0 | 3064 | 273 |
| 152.07 | 0.060 | 0 | 2999 | 259 | 0.058 | 0 | 3029 | 284 |
| 155.56 | 0.061 | 0 | 2982 | 264 | 0.059 | 0 | 3006 | 287 |
| 159.12 | 0.062 | 0 | 2901 | 259 | 0.060 | 0 | 2927 | 289 |
| 162.77 | 0.063 | 0.01 | 2842 | 256 | 0.061 | 0 | 2870 | 291 |
| 166.51 | 0.063 | 0.01 | 2795 | 264 | 0.061 | 0 | 2816 | 297 |
| 170.32 | 0.064 | 0.01 | 2833 | 281 | 0.062 | 0 | 2843 | 310 |
| 174.26 | 0.065 | 0.01 | 2761 | 280 | 0.063 | 0 | 2770 | 311 |
| 178.25 | 0.065 | 0.01 | 2753 | 292 | 0.063 | 0 | 2753 | 319 |
| 182.34 | 0.066 | 0.01 | 2676 | 284 | 0.064 | 0.01 | 2683 | 318 |
| 186.53 | 0.067 | 0.01 | 2619 | 288 | 0.065 | 0.01 | 2623 | 324 |
| 190.81 | 0.068 | 0.01 | 2571 | 286 | 0.066 | 0.01 | 2573 | 321 |
| 195.19 | 0.068 | 0.01 | 2541 | 300 | 0.066 | 0.01 | 2538 | 332 |
| 199.65 | 0.069 | 0.01 | 2533 | 302 | 0.067 | 0.01 | 2522 | 331 |
| 204.25 | 0.070 | 0.01 | 2513 | 310 | 0.068 | 0.01 | 2497 | 337 |
| 208.94 | 0.071 | 0.01 | 2464 | 313 | 0.069 | 0.01 | 2447 | 342 |
| 213.72 | 0.071 | 0.01 | 2403 | 305 | 0.069 | 0.01 | 2386 | 335 |
| 218.63 | 0.072 | 0.01 | 2309 | 296 | 0.069 | 0.01 | 2296 | 329 |
| 223.67 | 0.073 | 0.01 | 2273 | 296 | 0.071 | 0.01 | 2261 | 331 |
| 228.80 | 0.074 | 0.01 | 2263 | 309 | 0.071 | 0.01 | 2243 | 340 |

| $f$ | $\widehat{\sigma}_{\mathrm{L,mean}}$ | $\widehat{\sigma}_{\mathrm{L,std}}$ | $\widehat{\varepsilon}_{r,\mathrm{L,mean}}$ | $\widehat{\varepsilon}_{r,\mathrm{L,std}}$ | $\widehat{\sigma}_{\mathrm{T,mean}}$ | $\widehat{\sigma}_{\mathrm{T,std}}$ | $\widehat{\varepsilon}_{r,\mathrm{T,mean}}$ | $\widehat{\varepsilon}_{r,\mathrm{T,std}}$ |
|---|---|---|---|---|---|---|---|---|
| 234.02 | 0.075 | 0.01 | 2233 | 313 | 0.072 | 0.01 | 2210 | 342 |
| 239.40 | 0.075 | 0.01 | 2177 | 310 | 0.073 | 0.01 | 2154 | 341 |
| 244.91 | 0.076 | 0.01 | 2166 | 316 | 0.073 | 0.01 | 2137 | 343 |
| 250.51 | 0.076 | 0.01 | 2112 | 315 | 0.074 | 0.01 | 2081 | 340 |
| 256.27 | 0.077 | 0.01 | 2058 | 315 | 0.075 | 0.01 | 2028 | 341 |
| 262.16 | 0.078 | 0.01 | 2044 | 319 | 0.076 | 0.01 | 2012 | 344 |
| 268.17 | 0.080 | 0.01 | 2019 | 324 | 0.077 | 0.01 | 1985 | 349 |
| 274.33 | 0.080 | 0.01 | 1962 | 316 | 0.077 | 0.01 | 1928 | 339 |
| 280.63 | 0.081 | 0.01 | 2046 | 347 | 0.078 | 0.01 | 1997 | 359 |
| 287.05 | 0.081 | 0.01 | 1849 | 303 | 0.078 | 0.01 | 1817 | 328 |
| 293.66 | 0.082 | 0.01 | 1854 | 318 | 0.079 | 0.01 | 1816 | 338 |
| 300.39 | 0.083 | 0.01 | 1788 | 308 | 0.080 | 0.01 | 1753 | 330 |
| 307.29 | 0.084 | 0.01 | 1800 | 318 | 0.081 | 0.01 | 1758 | 336 |
| 314.33 | 0.085 | 0.01 | 1714 | 302 | 0.082 | 0.01 | 1679 | 323 |
| 321.54 | 0.085 | 0.01 | 1701 | 307 | 0.082 | 0.01 | 1661 | 324 |
| 328.94 | 0.087 | 0.02 | 1663 | 308 | 0.083 | 0.01 | 1622 | 324 |
| 336.49 | 0.087 | 0.02 | 1613 | 300 | 0.084 | 0.01 | 1575 | 317 |
| 344.20 | 0.088 | 0.02 | 1596 | 304 | 0.084 | 0.01 | 1555 | 318 |
| 352.10 | 0.089 | 0.02 | 1562 | 302 | 0.085 | 0.01 | 1520 | 315 |
| 360.19 | 0.090 | 0.02 | 1551 | 305 | 0.086 | 0.01 | 1506 | 316 |
| 368.47 | 0.090 | 0.02 | 1524 | 308 | 0.086 | 0.01 | 1476 | 315 |
| 376.90 | 0.091 | 0.02 | 1408 | 272 | 0.087 | 0.01 | 1376 | 290 |
| 385.55 | 0.092 | 0.02 | 1441 | 294 | 0.088 | 0.02 | 1400 | 304 |
| 394.40 | 0.093 | 0.02 | 1394 | 283 | 0.089 | 0.02 | 1356 | 297 |
| 403.46 | 0.093 | 0.02 | 1370 | 285 | 0.089 | 0.02 | 1327 | 294 |
| 412.71 | 0.093 | 0.02 | 1375 | 302 | 0.089 | 0.02 | 1322 | 300 |
| 422.19 | 0.095 | 0.02 | 1268 | 264 | 0.091 | 0.02 | 1232 | 276 |
| 431.88 | 0.095 | 0.02 | 1245 | 267 | 0.091 | 0.02 | 1206 | 275 |
| 441.79 | 0.097 | 0.02 | 1240 | 269 | 0.092 | 0.02 | 1200 | 278 |
| 451.93 | 0.098 | 0.02 | 1204 | 267 | 0.093 | 0.02 | 1164 | 274 |
| 462.31 | 0.098 | 0.02 | 1191 | 268 | 0.093 | 0.02 | 1148 | 273 |
| 472.92 | 0.099 | 0.02 | 1161 | 265 | 0.094 | 0.02 | 1119 | 270 |
| 483.78 | 0.099 | 0.02 | 1125 | 259 | 0.094 | 0.02 | 1084 | 263 |
| 494.88 | 0.100 | 0.02 | 1114 | 260 | 0.095 | 0.02 | 1071 | 263 |
| 506.25 | 0.100 | 0.02 | 1093 | 261 | 0.095 | 0.02 | 1048 | 261 |
| 517.86 | 0.101 | 0.02 | 1045 | 247 | 0.096 | 0.02 | 1005 | 250 |
| 529.72 | 0.102 | 0.02 | 1030 | 249 | 0.097 | 0.02 | 988 | 250 |
| 541.90 | 0.102 | 0.02 | 1007 | 249 | 0.097 | 0.02 | 965 | 250 |
| 554.34 | 0.103 | 0.02 | 981 | 238 | 0.098 | 0.02 | 939 | 238 |
| 567.05 | 0.103 | 0.02 | 945 | 233 | 0.098 | 0.02 | 905 | 233 |
| 580.08 | 0.104 | 0.02 | 931 | 233 | 0.098 | 0.02 | 890 | 232 |
| 593.39 | 0.104 | 0.03 | 883 | 220 | 0.098 | 0.02 | 846 | 221 |
| 607.02 | 0.106 | 0.03 | 857 | 216 | 0.100 | 0.02 | 821 | 217 |
| 620.93 | 0.105 | 0.03 | 873 | 232 | 0.099 | 0.02 | 829 | 226 |
| 635.18 | 0.107 | 0.03 | 837 | 219 | 0.101 | 0.02 | 798 | 217 |
| 649.76 | 0.107 | 0.03 | 815 | 216 | 0.101 | 0.02 | 776 | 213 |
| 664.67 | 0.108 | 0.03 | 788 | 211 | 0.102 | 0.02 | 751 | 209 |
| 679.94 | 0.107 | 0.03 | 765 | 204 | 0.102 | 0.02 | 728 | 202 |
| 695.55 | 0.108 | 0.03 | 737 | 197 | 0.102 | 0.02 | 701 | 195 |

| $f$ | $\widehat{\sigma}_{\mathrm{L,mean}}$ | $\widehat{\sigma}_{\mathrm{L,std}}$ | $\widehat{\varepsilon}_{r,\mathrm{L,mean}}$ | $\widehat{\varepsilon}_{r,\mathrm{L,std}}$ | $\widehat{\sigma}_{\mathrm{T,mean}}$ | $\widehat{\sigma}_{\mathrm{T,std}}$ | $\widehat{\varepsilon}_{r,\mathrm{T,mean}}$ | $\widehat{\varepsilon}_{r,\mathrm{T,std}}$ |
|---|---|---|---|---|---|---|---|---|
| 711.50 | 0.108 | 0.03 | 715 | 192 | 0.102 | 0.02 | 681 | 189 |
| 727.84 | 0.108 | 0.03 | 678 | 180 | 0.103 | 0.03 | 647 | 180 |
| 744.55 | 0.109 | 0.03 | 689 | 189 | 0.103 | 0.03 | 654 | 185 |
| 761.64 | 0.109 | 0.03 | 653 | 182 | 0.103 | 0.03 | 620 | 179 |
| 779.10 | 0.108 | 0.03 | 617 | 170 | 0.103 | 0.03 | 588 | 167 |
| 796.98 | 0.110 | 0.03 | 604 | 167 | 0.104 | 0.03 | 575 | 166 |
| 815.26 | 0.111 | 0.03 | 547 | 147 | 0.105 | 0.03 | 523 | 149 |
| 833.99 | 0.112 | 0.03 | 568 | 162 | 0.105 | 0.03 | 540 | 160 |
| 853.12 | 0.110 | 0.03 | 551 | 158 | 0.104 | 0.03 | 523 | 155 |
| 872.70 | 0.110 | 0.03 | 526 | 152 | 0.104 | 0.03 | 499 | 150 |
| 892.75 | 0.111 | 0.03 | 524 | 156 | 0.105 | 0.03 | 496 | 152 |
| 913.23 | 0.112 | 0.03 | 482 | 140 | 0.106 | 0.03 | 458 | 140 |
| 934.19 | 0.111 | 0.03 | 480 | 145 | 0.105 | 0.03 | 455 | 142 |
| 955.63 | 0.111 | 0.03 | 475 | 146 | 0.105 | 0.03 | 449 | 142 |
| 977.56 | 0.112 | 0.03 | 445 | 136 | 0.106 | 0.03 | 421 | 134 |
| 1000.00 | 0.112 | 0.03 | 428 | 130 | 0.106 | 0.03 | 405 | 126 |
